# Structural stability of DNA origami nanostructures in organic solvents†

**DOI:** 10.1039/d4nr02185a

**Published:** 2024-06-19

**Authors:** Eeva Enlund, Sofia Julin, Veikko Linko, Mauri A. Kostiainen

**Affiliations:** a Biohybrid Materials, Department of Bioproducts and Biosystems, Aalto University 00076 Aalto Finland mauri.kostiainen@aalto.fi; b Institute of Technology, University of Tartu 50411 Tartu Estonia; c LIBER Center of Excellence, Aalto University 00076 Aalto Finland

## Abstract

DNA origami nanostructures have attracted significant attention as an innovative tool in a variety of research areas, spanning from nanophotonics to bottom-up nanofabrication. However, the use of DNA origami is often restricted by their rather limited structural stability in application-specific conditions. The structural integrity of DNA origami is known to be superstructure-dependent, and the integrity is influenced by various external factors, for example cation concentration, temperature, and presence of nucleases. Given the necessity to functionalize DNA origami also with non-water-soluble entities, it is important to acquire knowledge of the structural stability of DNA origami in various organic solvents. Therefore, we herein systematically investigate the post-folding DNA origami stability in a variety of polar, water-miscible solvents, including acetone, ethanol, DMF, and DMSO. Our results suggest that the structural integrity of DNA origami in organic solvents is both superstructure-dependent and dependent on the properties of the organic solvent. In addition, DNA origami are generally more resistant to added organic solvents in folding buffer compared to that in deionized water. DNA origami stability can be maintained in up to 25–40% DMF or DMSO and up to 70–90% acetone or ethanol, with the highest overall stability observed in acetone. By rationally selecting both the DNA origami design and the solvent, the DNA origami stability can be maintained in high concentrations of organic solvents, which paves the way for more extensive use of non-water-soluble compounds for DNA origami functionalization and complexation.

## Abstract 2

The post-folding structural stability of DNA origami was studied in water-miscible organic solvents. While DNA origami remains stable in high acetone and ethanol concentrations, it degrades more rapidly in the presence of DMF and DMSO.

## Graphical abstract

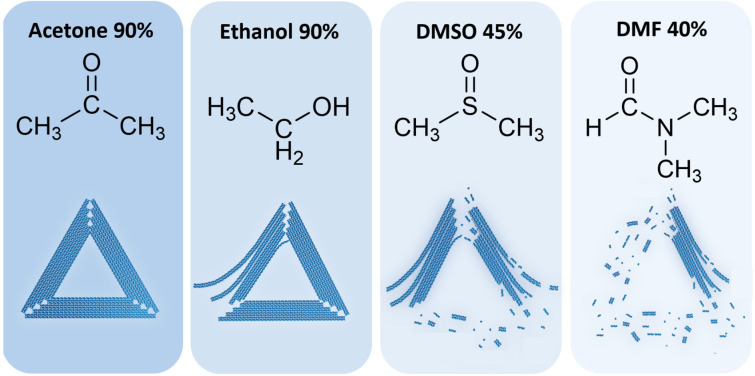

## Introduction

In the field of DNA nanotechnology, DNA is not merely used as a carrier of genetic information but instead as a nanoscale building material.^1,2^ An important step forward in this direction was taken in 2006 when DNA origami was introduced,^3^ thus significantly increasing the diversity and complexity of the DNA-based nanostructures. In the DNA origami technique, a long single-stranded scaffold is folded into a desired two- or three-dimensional (2D or 3D) shape by shorter, complementary oligonucleotides (staple strands).^3–6^ The synthesis of versatile DNA origami structures along with the ever-evolving design software^2,6,7^ has paved the way for applications in *e.g.* nanomedicine,^8,9^ nanorobotics,^10,11^ nanophotonics^12,13^ and bottom-up nanofabrication.^14–16^

The use of DNA origami in several potential applications is, however, still restricted by their limited structural stability under many application-relevant conditions. DNA origami structures are known to have limited stability for example in low-magnesium buffers,^17–19^ at elevated temperatures,^20–22^ in nuclease-rich environments,^23,24^ and in the presence of chaotropic salts.^25,26^ Research efforts have in recent years been undertaken to not only understand the factors affecting the DNA origami stability under different environmental conditions,^27,28^ but also to develop strategies for improving the DNA origami stability in relevant settings.^29–31^ The structural integrity has been revealed to be superstructure-dependent, and the design-specific parameters, such as lattice type, cross-over density, and staple strand lengths have been shown to affect both the structural and mechanical properties.^18,23,29^

**Fig. 1:**
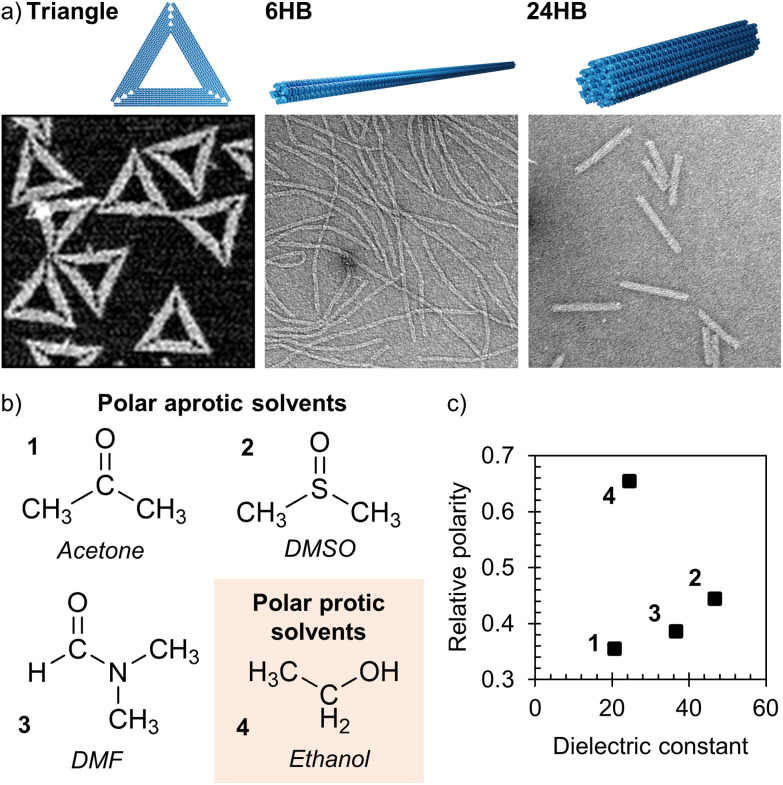
The DNA origami structures and organic solvents used in the study. (a) Atomic force microscopy (AFM) image of the triangles and transmission electron microscopy (TEM) images of the 6-helix bundles (6HB) and 24-helix bundles (24HB). The size of each microscopy image is 400 nm × 400 nm, and the TEM samples are negatively stained with 2% (w/v) uranyl formate. (b) Chemical structures of the chosen water-miscible polar organic solvents; acetone (1), dimethyl sulfoxide (DMSO, 2), dimethylformamide (DMF, 3) and ethanol (4). (c) The relative polarities and the dielectric constants for the numbered organic solvents used in the study.

As a template, DNA origami allows direct and precise positioning of oligonucleotide-functionalized molecular moieties onto it,^32,33^ while its negative (surface) charge facilitates electrostatic co-assembly with cationic compounds.^34–36^ However, many chemical compounds are poorly soluble in aqueous solutions, and therefore it would be preferable to perform the complexation in an organic solvent or in a solvent/water mixture. So far, the structural stability of DNA origami in organic solvents has been studied only to a limited extent,^37,38^ and there are no conclusive studies aiming to systematically investigate the DNA origami stability in such solvents. Thus, to address this knowledge gap, we study here the structural post-folding integrity of three different DNA origami structures in a selection of water-miscible organic solvents (Fig. 1). To evaluate the intactness of the DNA origami after solvent exposure, the structures were characterized with agarose gel electrophoresis (AGE) and transmission electron microscopy (TEM) or atomic force microscopy (AFM).

## Results and discussion

For investigating the structural post-folding stability of DNA origami, we used three different DNA origami structures; a triangle,^3^ a 6-helix bundle (6HB),^39^ and a 24-helix bundle (24HB)^40^ (Fig. 1a, ESI, Fig. S1–S3†). Furthermore, four water-miscible solvents, commonly employed in organic synthesis, were chosen as model solvents for this study; acetone, dimethyl sulfoxide (DMSO), dimethylformamide (DMF) and ethanol (Fig. 1b). These solvents have distinct physical properties (Fig. 1c), and they were selected to represent both polar protic and polar aprotic solvents (Fig. 1b).

**Fig. 2:**
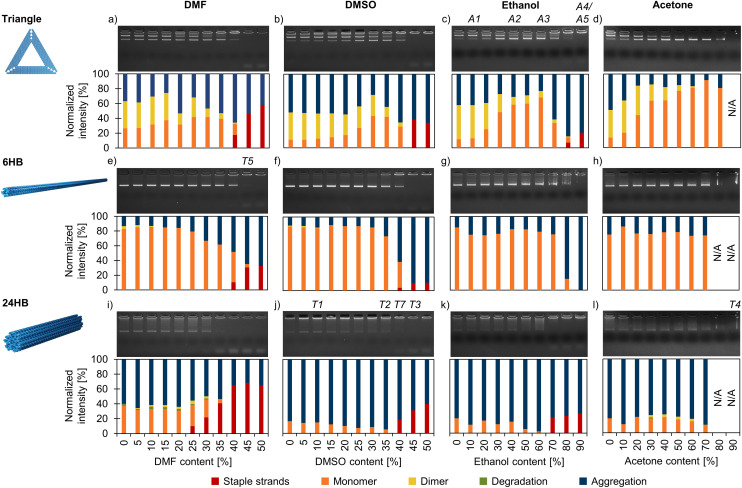
Agarose gel electrophoresis (AGE) – based analysis of the structural stability of DNA origami in the folding buffer (FOB) with varied amounts of added organic solvents. The triangles (a–d), 6HBs (e–h), and 24HBs (i–l) were exposed to DMF (a, e and i), DMSO (b, f and j), ethanol (c, g and k), and acetone (d, h and l). The top panel displays the agarose gel, whereas the bottom panel shows the distribution of staple strands, monomers, dimers, degradation, and aggregation for each sample determined by analyzing the ethidium bromide intensity of the discreted bands. AFM and TEM images of the samples marked A1–A4 and T1–T4, respectively, are displayed in Fig. 3, while TEM images of samples T5 and T7 are shown in Fig. 4.

The structural stability of the DNA origami structures in the organic solvents was studied by incubating them for 24 h with varying volume percentages of the solvents in folding buffer (FOB, 1× TAE, 12.5 mM MgCl_2_ for the triangle and the 6HB, 1× TAE, 17.5 mM MgCl_2_ for the 24HB). The structural integrity of the DNA origami samples after the solvent incubation was initially analyzed by AGE (Fig. 2, ESI, Fig. S4–S15†). Typically, intact DNA origami exhibit different electrophoretic mobility in the gel compared to degraded structures and staple strands, and therefore AGE can be readily used to evaluate the intactness of the DNA origami structure after solvent exposure. Similarly, also DNA origami monomers have a higher electrophoretic mobility than DNA origami dimers (and oligomers), while heavily aggregated structures are usually completely retained in the gel loading well. Therefore, by monitoring the ethidium bromide fluorescence intensity of the discreted gel bands, we quantitatively estimated the composition of each of the DNA origami samples.

Regardless of the DNA origami design, origami structures remained more intact in ethanol and acetone than in DMSO and DMF. As the dielectric constant of the solvent is known to play a role in the structural stability of nucleic acids,^41^ the low stability could be attributed to high dielectric constants (see Fig. 1c). In general, interactions between the phosphate groups of the DNA backbone and the Mg^2+^ ions in the folding buffer allow the adjacent helices of DNA origami to pack closely, and these interactions have therefore an important role in maintaining the overall DNA origami stability.^27^ In solutions with low dielectric constants, the interactions between the phosphate groups and cations are typically stronger,^41,42^ suggesting enhanced stability. However, as we still observed DNA origami degradation in solvent/buffer mixtures with lower dielectric constants than that of the folding buffer, the DNA origami stability in such solutions has to be affected also by other factors, such as polarity and pH of the solvent and the ability of the solvent to form hydrogen bonds.

From the solvents tested, DNA origami showed the lowest stability in DMF. Some staple strands started to detach from the 24HBs already in 25% (v/v) DMF (Fig. 2i), while the triangles and 6HBs degraded in 40% DMF (Fig. 2a and e). In DMSO, all structures remained stable until a solvent content of ∼40%, but the rigid 24HBs degraded slightly faster than the more flexible triangles and 6HBs. These observations are in agreement with the previous studies that have demonstrated that flexible quasi-1D and 2D structures are more stable in non-conventional DNA origami buffers than 3D structures.^17,43,44^ Moreover, the used 6HB design is particularly flexible due to its low staple crossover density, which enables the structure to respond to changing environmental conditions by structural adjustments rather than disintegrating.^18^ In ethanol, all DNA origami designs maintained their structural integrity up to 60% solvent content (Fig. 2c, g and k). The triangles and the 24HBs started to lose staple strands at 80% and 70% ethanol, respectively, but the 6HBs did not show any degradation even at 90% ethanol. However, when the ethanol content exceeded 70%, all DNA origami designs appeared heavily aggregated. Ethanol precipitation is widely used for concentrating DNA solutions, and similarly it has been used to purify and precipitate DNA origami.^45^ In acetone, no staple strand disintegration from the structures was observed, which suggests that all three DNA origami designs remained intact at least up to 70–80% (Fig. 2d, h and l). At 80–90% acetone, evaporation and surface tension gradient formation started to deform the gel bands and thus made them inaccessible for detailed analysis. Furthermore, as reported earlier,^46^ the triangles are prone to stack vertex-to-vertex and form dimers (Fig. 2a–d). Surprisingly, these stacking interactions can be attenuated in organic solvents, as there were almost no dimers observed in high ethanol and acetone containing solutions (Fig. 2c and d).

**Fig. 3:**
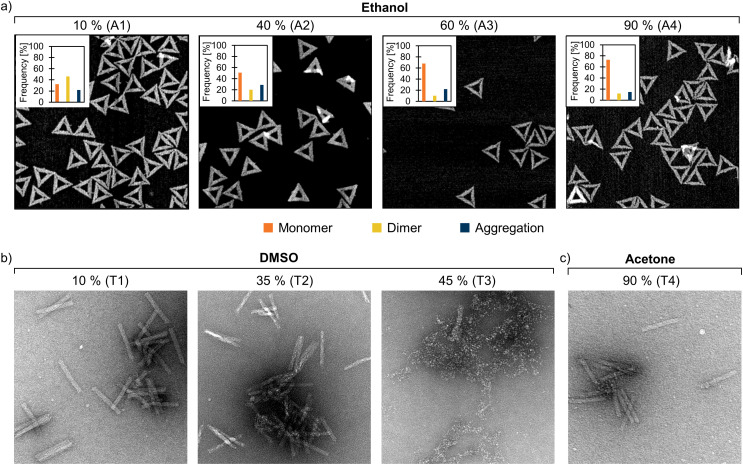
TEM and AFM images of DNA origami in FOB with varied amounts of added organic solvents. (a) The triangles in 10–90% (v/v) ethanol. The distribution of monomers, dimers, and aggregates (determined from the AFM images) are indicated in the upper left inset of each AFM image. The number of individual structures analyzed for each sample was *n* = 100. (b) The 24HBs in 10–45% (v/v) DMSO. (c) The 24HBs in 90% (v/v) acetone. The size of the AFM images is 1 μm × 1 μm, while the TEM images are 400 nm × 400 nm. The TEM samples are negatively stained with 2% (w/v) uranyl formate. For clarity, the AFM samples (A1–A4) and TEM samples (T1–T4) are also marked in the agarose gels in Fig. 2.

To further support the obtained AGE results and to visualize the DNA origami structures after solvent exposure, we additionally imaged the samples by AFM (triangles, Fig. 3a, ESI, Fig. S16–S26†) and TEM (6HB and 24HB, Fig. 3b and c, ESI, Fig. S27–S54†). The triangle samples with different amounts of ethanol were deposited on a mica substrate and imaged using AFM directly after the 24 h solvent incubation. The trend of decreasing number of dimers with increasing solvent concentration was also evident in the AFM images (Fig. 3a). As the ethanol content increased from 10% to 90%, the proportion of the dimer-forming triangles in the sample consistently decreased from ∼45% to ∼10%. Previously, DNA origami have been demonstrated to remain intact and even fold at 10% ethanol.^47^ Here, the triangles appeared mostly intact up to 90% ethanol, although the AGE indicates that some staple strands might get detached from the triangles at such high ethanol concentrations.

Particularly at high solvent concentrations, the organic solvent may deteriorate the Formvar TEM grid and cause drying artifacts.^38^ Therefore, to allow visual inspection of the samples (apart from the AFM samples discussed above), the organic solvent was removed by poly(ethylene glycol) (PEG) precipitation^48^ prior to the TEM/AFM sample preparation. After the PEG purification, the samples were resuspended in 1× FOB. AGE further confirmed that the intact DNA origami can be recovered after the PEG precipitation, but for interpreting the results it is worth noting that the purification process effectively removes both unfolded scaffold and staple strands (ESI, Fig. S4–S15†). Therefore, it is possible that PEG purification could have removed also degraded DNA origami structures from the sample. Moreover, it is noteworthy to mention that partially degraded DNA origami can also be healed by the addition of Mg^2+^ ions.^19^ The structural stability of the samples was studied with AGE both prior and after the PEG purification (ESI, Fig. S4–S15†). Although the addition of Mg^2+^ ions could potentially repair some degraded structures, the AGE results showed that the possible healing effect was not remarkable. In agreement with the AGE results, the TEM images suggest that the 24HB remain intact until a DMSO concentration of 40% (Fig. 3b). However, as the DMSO content exceeded 40%, the 24HBs started to degrade. The deterioration of 24HBs can be seen as a fragmentation starting from the ends of the structure and a gradual unfolding of the structure (ESI, Fig. S41–S48†). This is also in line with the previous studies that report DNA origami being stable in 10% (ref. 49) and 32% DMSO,^50^ but significantly degraded in 40% DMSO.^51^ For the triangles and the 6HBs, the degradation mechanisms were different. When the solvent content increased, the triangles started to deteriorate by swelling, after which the structure folded inwards and ultimately shrank (ESI, Fig. S16–S18†). The long and flexible 6HB, on the other hand, first wrapped into “ball of yarn”-like aggregates, which was followed by fragmentation and complete degradation of the structure (ESI, Fig. S27–S34†). DNA origami is known to maintain structural stability in high acetone concentrations,^38^ which was also confirmed by both TEM and AFM imaging (ESI, Fig. S24–S26, S38–S40 and S52–S54†). Even the 24HB that readily disintegrated in most of the tested organic solvents showed no degradation in 90% acetone (Fig. 3c). This suggests that DNA origami can be pelleted by organic solvents and further recovered from the aggregates by removing the solvent (with *e.g.* PEG precipitation).

It is also important to investigate the possible limitations of DNA origami at lower salt concentrations, as high salt concentrations are often undesirable for many technological applications.^17,28,52^ As already discussed, Mg^2+^ ions or other cations are typically added to the DNA origami folding buffer to screen the electrostatic repulsion between the DNA backbones.^27^ However, it has been demonstrated that after folding, DNA origami can be transferred to deionized water or other low ionic strength buffers (μM range Mg^2+^ concentration) without losing their structural integrity.^17,18^ Plausibly, this is due to the residual Mg^2+^ ions that remain bound to the phosphate groups and thus stabilize the DNA origami. Here, we hypothesized that these residual Mg^2+^ ions might be affected by organic solvents, and that addition of organic solvents to DNA origami in deionized water could therefore have a destabilizing effect. To confirm this hypothesis, we repeated the same experiments conducted in folding buffer also in deionized water (μM range Mg^2+^ concentration).

**Fig. 4:**
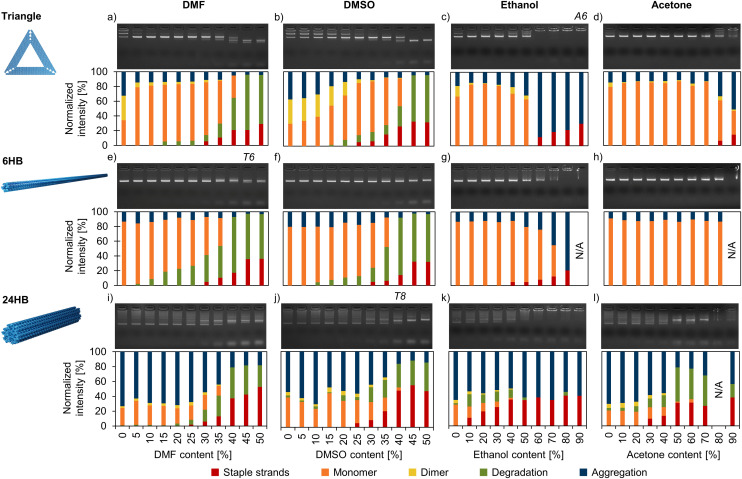
Agarose gel electrophoresis (AGE) – based analysis of the structural stability of DNA origami in deionized water with varied amounts of added organic solvents. The triangles (a–d), 6HBs (e–h), and 24HBs (i–l) were exposed to DMF (a, e and i), DMSO (b, f and j), ethanol (c, g and k), and acetone (d, h and l). The top panel displays the agarose gel, whereas the bottom panel shows the distribution of staple strands, monomers, dimers, degradation, and aggregation for each sample determined by analyzing the ethidium bromide intensity of the discreted bands. AFM and TEM images of the samples marked A6 and T6 and T8, respectively, are shown in Fig. 5.

As expected, the DNA origami stability in organic solvents is remarkably lower in the absence of stabilizing Mg^2+^ ions, and typically the staple strands start to detach from the structures at 10–15%-points lower solvent content than in FOB-based mixtures (Fig. 4, ESI, Fig. S55–S66†). As an example, in 40% DMF in deionized water, the proportion of intact structures of the triangle population was only 30%, whereas in 40% DMF in FOB, the fraction was ∼80% (Fig. 4a). For DMF and DMSO, the AGE also suggests that the solvent-induced DNA origami degradation mechanisms are different in FOB and deionized water. When the solvent content was so high that the staple strands started to detach from the origami structures in FOB-based mixtures, the structures appeared also heavily aggregated (Fig. 2a, b, e and f–j). However, in deionized water, the structures disintegrated without noticeable aggregation (Fig. 4a, b, e and f–j). Similarly as for FOB, the 24HB was found to be the least stable structure, and in deionized water it degraded rapidly also in both ethanol and acetone (Fig. 4i–l). In line with the earlier results,^35^ the triangles and 6HBs remained structurally stable at low ethanol concentrations in deionized water, but at high ethanol concentrations the degradation appeared more pronounced than in FOB (Fig. 4c, g *vs.* 2c, g).

**Fig. 5:**
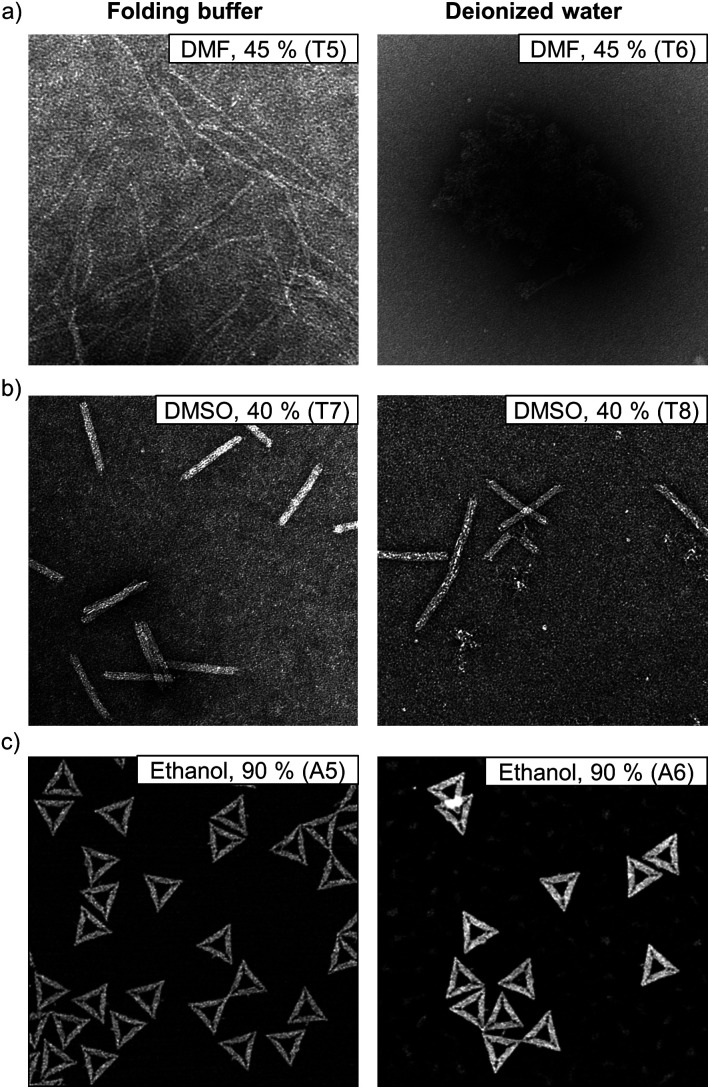
Comparison of DNA origami structures when exposed to organic solvents in folding buffer and in deionized water. (a) TEM images of 6HBs in FOB and in deionized water with 45% (v/v) of DMF. (b) TEM images of 24HBs in FOB and in deionized water with 40% (v/v) of DMSO. (c) AFM images of the triangles in FOB and in deionized water with 90% (v/v) of ethanol. The size of the AFM images is 1 μm × 1 μm, while the TEM images are 400 nm × 400 nm. The TEM samples are negatively stained with 2% (w/v) uranyl formate. For clarity, the TEM samples T5 and T7 and the AFM sample A5 are also marked in Fig. 2. The TEM samples T6 and T8 and the AFM sample A6 are marked in Fig. 4.

In addition to AGE, we again used AFM (ESI, Fig. S67–S77†) and TEM (ESI, Fig. S78–S105†) to characterize the structures in deionized water after the solvent exposure. Similarly to the samples in 1× FOB, the samples were PEG purified before the imaging in order to remove the organic solvent. The samples were resuspended in 1× FOB, and since the Mg^2+^ ion concentration increases significantly after the PEG purification it should be noted that the added Mg^2+^ ions might heal some of the degraded structures.^19^ Nevertheless, AGE, conducted before and after the PEG purification, verify that the structure healing does not remarkably alter our results (ESI, Fig. S55–S66†). The AFM and TEM images supported the AGE results and clearly indicated that the DNA origami degraded at lower organic solvent concentrations in deionized water than in FOB (Fig. 5). For the 6HB in 45% DMF in FOB, AGE suggested that ∼60% of the structures maintained their integrity (Fig. 2e), while only ∼10% of the 6HBs were intact in 45% DMF in deionized water (Fig. 4e). The same trend was also observed in TEM, where the 6HBs were still intact in 45% DMF, but completely deteriorated in deionized water (Fig. 5a). Similarly, for 24HBs in 40% DMSO in FOB, the fraction of intact structures was ∼80% (Fig. 2j), while in deionized water the fraction was reduced to ∼20% (Fig. 4j). As seen from the TEM images, the 24HBs in 40% DMSO in FOB appeared unaffected, while in 40% DMSO in deionized water the 24HB had already started to degrade (Fig. 5b). In both FOB and deionized water – based mixtures, the triangles remained mostly intact even at 90% ethanol (Fig. 5c). However, the triangles in deionized water were more aggregated than in FOB and they had also started to disintegrate. This is in line with the AGE results, which indicated that ∼80% and∼70% of the triangles were intact in 90% ethanol in FOB and 90% ethanol in deionized water, respectively (Fig. 2c and 4c).

## Conclusions

Herein, we have demonstrated the effects various water-miscible and polar organic solvents have on the structural post-folding stability of DNA origami while in FOB (mM range Mg^2+^ concentration) and in deionized water (μM range Mg^2+^ concentration). The results indicate that the DNA origami is more resistant to organic solvents in FOB compared to deionized water, suggesting that the Mg^2+^ ions provide a stabilizing effect in organic solvents. The structural stability of the DNA origami inorganic solvents is highly superstructure-dependent, and as observed earlier also in low-magnesium buffers,^17^ the flexible 6HB is the most stable structure while the rigid 24HB is the least stable. In addition, the structural stability correlates with the dielectric constant of the organic solvent – generally DNA origami appears to be more stable in organic solvents with low dielectric constants, such as acetone.

Our results demonstrate that by carefully selecting the solvent components and the DNA origami design, the DNA origami integrity can be maintained even at very high organic solvent concentrations. Therefore, we believe that our study enables a more extensive use of water-insoluble compounds for functionalization and complexation with DNA origami. Furthermore, the work could also pave the way for more sophisticated *in situ* reactions taking place on top of the DNA origami, such as *e.g. in situ* polymerization.^53^

## Materials and methods

### Materials

Unless otherwise specified, all chemicals were purchased from commercial suppliers and used as received. Deionized water (Milli-Q grade) was utilized in all experiments.

For the preparation of the DNA origami structures, the scaffolds were purchased from Tilibit Nanosystems (*c* = 100 nM), while the single-stranded staple strands were purchased from Integrated DNA Technologies. The 50× TAE buffer (2 M tris(hydroxymethyl)aminomethane (Tris), 1 M acetic acid, 50 mM ethylenediaminetetraacetic acid (EDTA), pH 8.4) was purchased from Thermo Fischer Scientific. Sodium chloride, magnesium chloride, and poly(ethylene glycol) were purchased from Sigma-Aldrich. DMF and DMSO were purchased from Sigma-Aldrich, ethanol from Altia, and acetone from Honeywell. For the AGE, the agarose was purchased from Meridian Bioscience and the ethidium bromide from Sigma-Aldrich. The gel loading dye solution (0.25% bromophenol blue, 0.25% xylene cyanol, 40% sucrose), which was purchased from Sigma-Aldrich, was diluted 1 : 30 in 40% (w/v) sucrose or 75% glycerol prior to use. For the TEM sample staining, uranyl formate was purchased from Electron Microscopy Sciences.

### Folding and purification of DNA origami

The design and staple strands for the triangle,^3^ the 6HB,^39^ and the 24HB^40^ DNA origami can be found from their original sources.

The DNA origami structures were folded in one-pot reactions using thermal annealing. The triangle and the 6HB structures were folded using the circular p7249 scaffold (final concentration of 20 nM) and 10× excess of staple strands in FOB containing1× TAE and 12.5 mM MgCl_2_. The triangle and 6HB structures were thermally annealed using a ProFlex PCR system and the following thermal ramp: (1) cooling from 90 to 70 °C at a rate of −0.2 °C/8 s; (2) cooling from 70 to 60 °C at a rate of −0.1 °C/8 s; (3) cooling from 60 to 27 °C at a rate of −0.1 °C/2 min; (4) cooling down to 20 °C and stored until the program was manually stopped.

The 24HB structures were folded using the circular p7560 scaffold (final concentration of 20 nM) and 10× excess of staple strands in FOB containing 1× TAE and 17.5 mM MgCl_2_. The 24HB structures were thermally annealed using a ProFlex PCR system and the following thermal ramp: (1) cooling from 65 to 59 °C at a rate of 1 °C/15 min; (2) cooling down from 59 to 40 °C at a rate of 0.25 °C/45 min; (3) cooling down to 20 °C and stored until the program was manually stopped.

After the folding, the excess staple strands were removed using PEG precipitation.^48^ The DNA origami solution was diluted 4-fold with 1× FOB and subsequently mixed 1 : 1 with PEG precipitation buffer (15% PEG 8000, 1× TAE, 505 mM NaCl). The mixture was centrifuged at 14 000*g* for 30 min at room temperature using an Eppendorf 5424R microcentrifuge. The supernatant was removed, and the pelleted DNA origami resuspended in 1× FOB to 0.25–1× of the initial reaction volume. To ensure that the pellet dissolved completely, the samples were incubated overnight at 30 °C under continuous shaking at 600 rpm using an Eppendorf Thermomixer C.

If needed, the DNA origami solutions were upconcentrated using 100 kDa MWCO centrifugal filters (0.5 mL, Amicon Ultra, Merck). Before use, the centrifugal filter was washed with 500 μL of 1× FOB by centrifuging at 14 000*g* for 5 min at room temperature using an Eppendorf 5424R microcentrifuge. 200–400 μL of the PEG-purified DNA origami solution (*c* = 20–80 nM) was added to the centrifugal filter and the centrifugal filter was centrifuged for 20 min at 6000*g*. Subsequently, the DNA origami solution was collected into a new tube by inverting the filter unit and centrifuging at 2000*g* for 2.5 min.

For the experiments in deionized water, the PEG-purified DNA origami solutions were transferred to deionized water using spin-filtration with 100 kDa molecular weight cut off (MWCO) spin-filters (0.5 mL, Amicon Ultra, Merck).^35^ First, the filter was washed with 500 μL of deionized water by centrifuging the device for 5 min at 14 000*g* using an Eppendorf 5424R microcentrifuge. 120 μL of PEG-purified origami solution was added to the centrifugal filter together with 120 μL of deionized water. The filter was centrifuged at 6000*g* for 10 min at room temperature. The flow-through was discarded. 251 μL of deionized water was added to the filter after which the centrifugal filter was centrifuged at 6000*g* for 10 min at room temperature. The flow-through was saved for later use. The DNA origami solution was collected into a new tube by inverting the filter unit and centrifuging at 2000*g* for 2.5 min. Before further use, the collected DNA origami solution was diluted with the flow-through from step 2 to a final concentration of 100 nM.

The DNA origami concentration was estimated from the absorbance at 260 nm using Beer–Lambert law. The absorbance was measured using a BioTek Eon microplate spectrophotometer, a Take3 microvolume plate, and a sample volume of 2 μL. The concentration was estimated as the average of three separate measurements. The molar extinction coefficient was estimated based on the number of non-hybridized and hybridized nucleotides in the DNA origami unit.^54^ The extinction coefficient was estimated as 0.97 × 10^8^ M^−1^ cm^−1^ for the triangle, 0.98 × 10^8^ M^−1^ cm^−1^ for the 6HB, and 1.05 × 10^8^ M^−1^ cm^−1^ for the 24HB.

### Incubation of DNA origami in organic solvents

The DNA origami structures (final concentration of 5 nM) were incubated in either 1× FOB or deionized water with varying amounts of the organic solvents at room temperature for 24 h. For the samples originally in 1× FOB, the final concentration after the solvent addition was also adjusted to 1× FOB (by adding 20× FOB). If not stated otherwise, the samples were analysed as such (with the organic solvent still present) by agarose gel electrophoresis, but for the TEM and AFM, the organic solvent was removed by PEG precipitation. The DNA origami solution containing organic solvent was mixed 1 : 1 with 15% (w/v) PEG8000, 1× TAE, 505 mM NaCl (for the samples in 1× FOB) or 15% (w/v) PEG8000, 2× TAE, 505 mM NaCl (for the samples in deionized water), after which the mixtures was centrifuged for 30 min at 14 000*g* using an Eppendorf 5424R microcentrifuge. The supernatant was removed, and the DNA origami pellet was resupended in 1× FOB. The samples were incubated overnight at room temperature before the TEM and AFM sample preparation.

### Agarose gel electrophoresis

A 2% (w/v) agarose gel was prepared in 1× TAE supplemented with 11 mM MgCl_2_ and 0.46 μg mL^−1^ ethidium bromide (EtBr). Before loading the samples into the gel pockets, gel loading dye solution was added to the samples (volume of 20 μL, *c*_origami_ = 5 nM). For the PEG-purified samples and the non-PEG-purified samples containing DMF and DMSO, 4 μL of 0.0083% bromophenol blue, 0.0083% xylene cyanol, 40% sucrose was added as loading dye solution while 18 μL of 0.0083% bromophenol blue, 0.0083% xylene cyanol, 1.3% sucrose, 75% glycerol was added as loading dye solution for the non-PEG-purified samples containing ethanol and acetone. The gel was run for 45 min using a BioRad Wide Mini-Sub Cell GT System and a BioRad PowerPac Basic power supply and a constant voltage of 90 V. The gel was run in a buffer containing 1× TAE and 11 mM MgCl_2_ while kept on an ice bath. After the run, the gel was visualized by ultraviolet light using a BioRad Gel Doc XR + documentation system.

For the non-PEG purified samples, the composition of each band in the agarose gel was quantitatively estimated using the gel analysis tool in ImageJ. The areas of the obtained gel peaks was determined using OriginLab. Initially, the baseline was subtracted by the PeakAnalyzer tool, after which the areas of the gel peaks were estimated using the integrate gadget.

### Transmission electron microscopy

The TEM samples were prepared on Formvar carbon-coated copper grids (FCF400-Cu, Electron Microscopy Science) mainly following the protocol by Castro *et al.*^55^ Prior to sample deposition, the grids were glow-charged with 15 s oxygen plasma flash (Gatan Solarus). 3 μL of the sample solution (*c*_origami_ ∼ 5 nM) was deposited onto the TEM grid and incubated for 3 min before blotted away against filter paper. Subsequently, the samples were negatively stained with 2% (w/v) aqueous uranyl formate solution containing 25 mM NaOH. First, the TEM grid was immersed into a 5 μL droplet of stain solution and the stain was immediately blotted away using filter paper. Following this, the sample was immersed into a 20 μL droplet of the stain solution and incubated for 45 s before excess stain was blotted away with filter paper. The samples were left to dry under ambient conditions for at least 15–20 min prior to imaging. The TEM images were obtained using FEI Tecnai 12 Bio-Twin microscope operated at an acceleration voltage of 120 V. The images were processed using ImageJ.

### Atomic force microscopy

20 μL of the sample solution (*c*_origami_ ∼ 1–2 nM) was deposited directly onto a freshly cleaved mica surface (15 mm × 15 mm, grade V1, Electron Microscopy Science) and incubated for 1 min at room temperature. Subsequently, the mica was washed three times with 100 μL of deionized water, after which the mica was dried thoroughly using direct nitrogen flow. The AFM images were recorded with a Dimension Icon AFM (Bruker) using ScanAsyst in Air Mode and ScanAsyst-Air probes (Bruker). The AFM images were recorded with a resolution of 512 × 512 pxl, a scan rate of 0.5 Hz and a scan size of 2 μm × 2 μm or 3 μm × 3 μm. The images were processed (row alignment and height scale adjustment) using Gwyddion open source software (v. 2.65).^56^

## Conflicts of interest

The authors declare no conflicts of interest.

## Supplementary Material

NR-016-D4NR02185A-s001

## Data Availability

The data supporting this article have been included as part of the ESI.†
