# Supplementary material for: Structural stability of DNA origami nanostructures in organic solvents
Source: Nanoscale. 2024 Jun 19;16(28):13407–15. doi: 10.1039/d4nr02185a (PMC11256221; doi:10.1039/d4nr02185a)
Supplement: NR-016-D4NR02185A-s001 [file NR-016-D4NR02185A-s001.pdf]

Electronic Supplementary Material (ESI) for Nanoscale.
This journal is © The Royal Society of Chemistry 2024

Supporting Information

Structural stability of DNA origami nanostructures in organic solvents

Eeva Enlund‡[a], Sofia Julin‡[a], Veikko Linko[a,b] & Mauri A. Kostiainen[a,c*]

[a] Biohybrid Materials, Department of Bioproducts and Biosystems, Aalto University, P.O. Box 16100, 00076 Aalto, Finland

[b] Institute of Technology, University of Tartu, Nooruse 1, 50411 Tartu, Estonia

[c] LIBER Center of Excellence, Aalto University, P.O. Box 16100, 00076 Aalto, Finland

[*]Correspondence and requests for materials should be addressed to mauri.kostiainen@aalto.fi

‡ These authors contributed equally to this work

Contents

		Page
**1**	**Characterization of DNA origami structures**	**S3**
**2**	**Additional agarose gels in folding buffer**	**S6**
2.1	DNA origami triangle in folding buffer	S6
2.2	6HB in folding buffer	S10
2.3	24HB in folding buffer	S14
**3**	**Additional AFM images in folding buffer**	**S18**
3.1	DNA origami triangle in folding buffer exposed to DMF	S18
3.2	DNA origami triangle in folding buffer exposed to DMSO	S21
3.3	DNA origami triangle in folding buffer exposed to ethanol	S23
3.4	DNA origami triangle in folding buffer exposed to acetone	S26
**4**	**Additional TEM images in folding buffer**	**S29**
4.1	6HB in folding buffer exposed to DMF	S29
4.2	6HB in folding buffer exposed to DMSO	S33
4.3	6HB in folding buffer exposed to ethanol	S37
4.4	6HB in folding buffer exposed to acetone	S40
4.5	24HB in folding buffer exposed to DMF	S43
4.6	24HB in folding buffer exposed to DMSO	S47
4.7	24HB in folding buffer exposed to ethanol	S51
4.8	24HB in folding buffer exposed to acetone	S54
**5**	**Additional agarose gels in deionized water**	**S57**
5.1	DNA origami triangle in deionized water	S57
5.2	6HB in deionized water	S61
5.3	24HB in deionized water	S65
**6**	**Additional AFM images in deionized water**	**S69**
6.1	DNA origami triangle in deionized water exposed to DMF	S69
6.2	DNA origami triangle in deionized water exposed to DMSO	S72
6.3	DNA origami triangle in deionized water exposed to ethanol	S75
6.4	DNA origami triangle in deionized water exposed to acetone	S78
**7**	**Additional TEM images in deionized water**	**S80**
7.1	6HB in deionized water exposed to DMF	S80
7.2	6HB in deionized water exposed to DMSO	S84
7.3	6HB in deionized water exposed to ethanol	S88
7.4	6HB in deionized water exposed to acetone	S91
7.5	24HB in deionized water exposed to DMF	S94
7.6	24HB in deionized water exposed to DMSO	S98
7.7	24HB in deionized water exposed to ethanol	S102
7.8	24HB in deionized water exposed to acetone	S105

1. Characterization of DNA origami structures

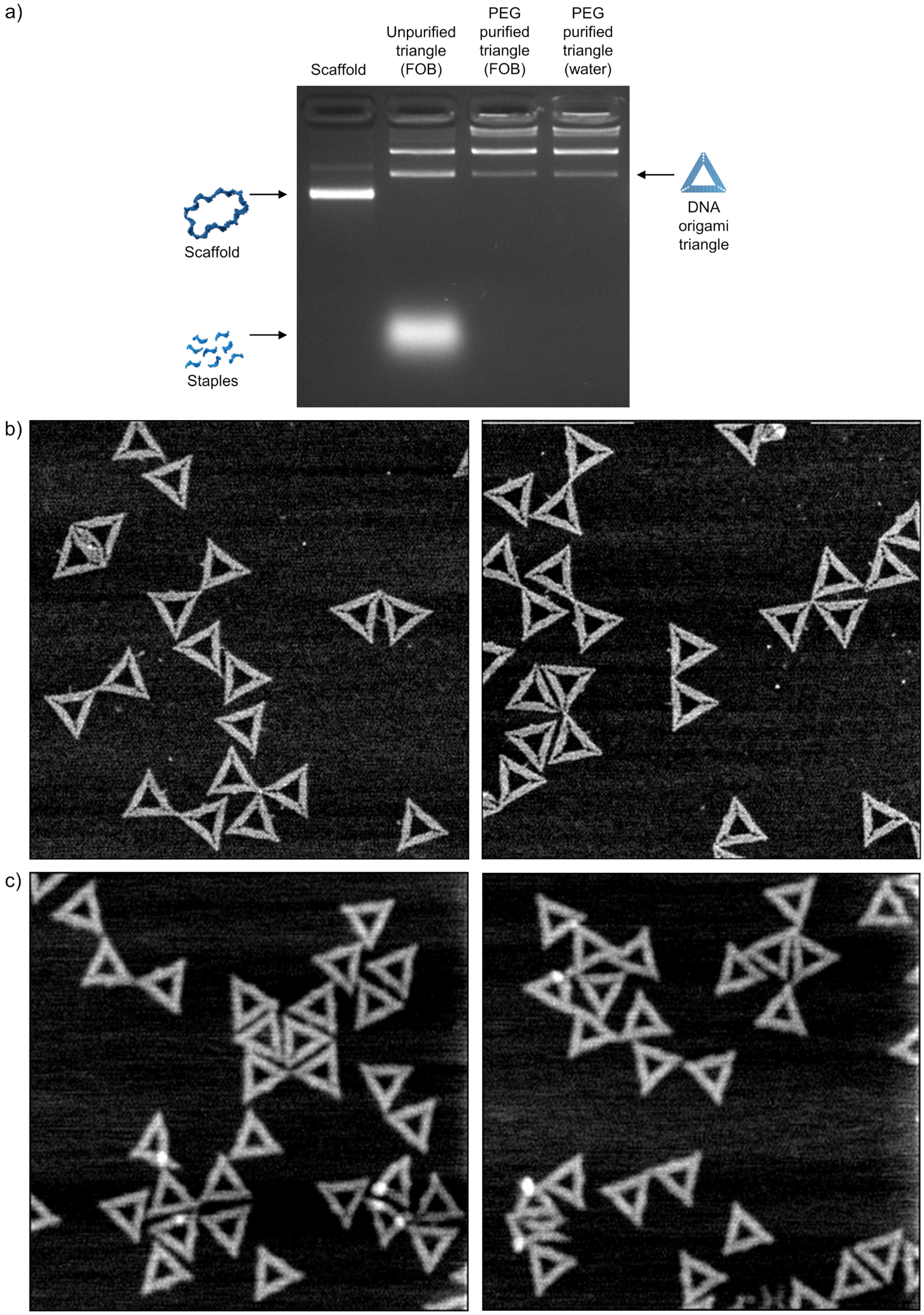

Figure S1. a) Characterization of the folding, poly(ethylene glycol) (PEG) purification and buffer exchange of DNA origami triangle by agarose gel electrophoresis (AGE). The p7249 scaffold concentration is 30 nM and the DNA origami triangle concentration is 15 nM in the gel. Atomic force microscopy (AFM) images of the DNA origami triangle in b) $1\times$ folding buffer (FOB, $1\times$ TAE, 12.5 mM $MgCl_2$), and c) deionized water. The size of the AFM images is 1 μm $1\times$ 1 μm.

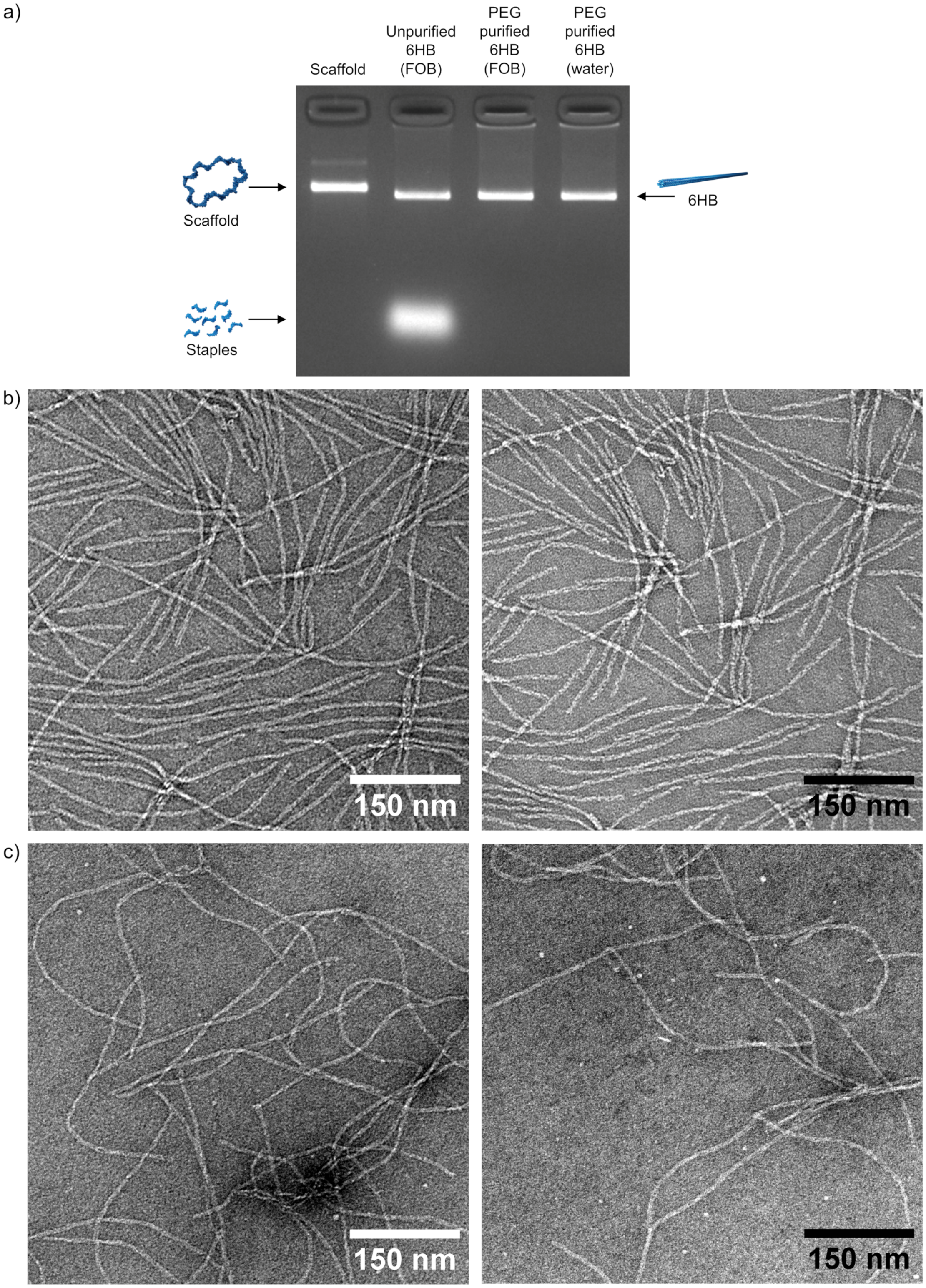

Figure S2. a) Characterization of the folding, PEG purification and buffer exchange of the 6-helix bundle (6HB) DNA origami by AGE. The p7249 scaffold concentration is 30 nM and the 6HB concentration is 15 nM in the gel. Transmission electron microscopy (TEM) images of the 6HB in b) 1× FOB (1× TAE, 12.5 mM $MgCl_2$), and c) deionized water. The TEM samples are negatively stained with uranyl formate (2% (w/v)).

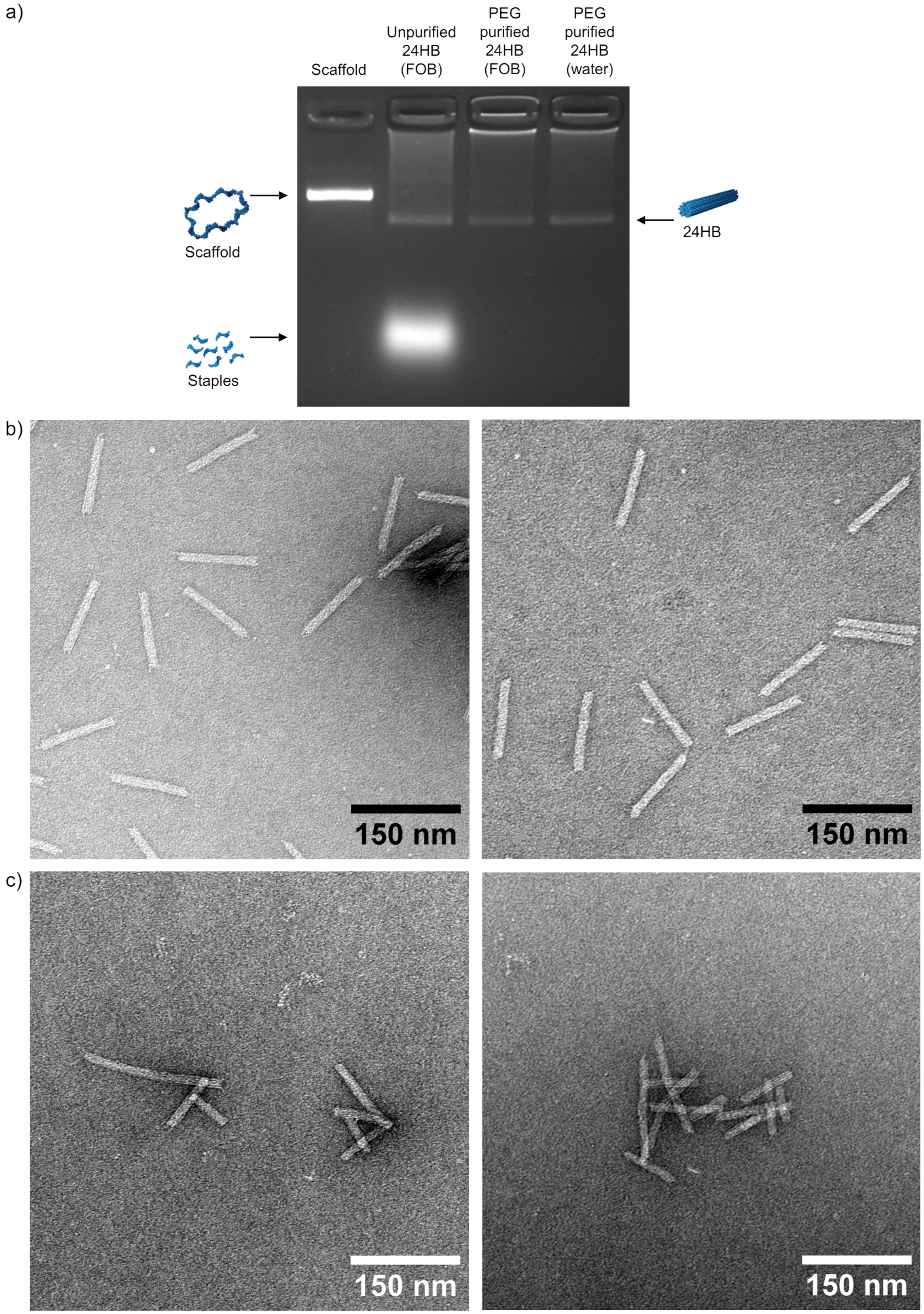

Figure S3. a) Characterization of the folding, PEG purification and buffer exchange of the 24-helix bundle (24HB) DNA origami by AGE. The p7560 scaffold concentration is 30 nM and the 24HB concentration is 15 nM in the gel. TEM images of the 24HB in b) $1\times$ FOB ($1\times$ TAE, 17.5 mM $MgCl_2$), and c) deionized water. The TEM samples are negatively stained with uranyl formate (2% (w/v)).

2. Additional agarose gels in folding buffer

2.1. DNA origami triangle in folding buffer

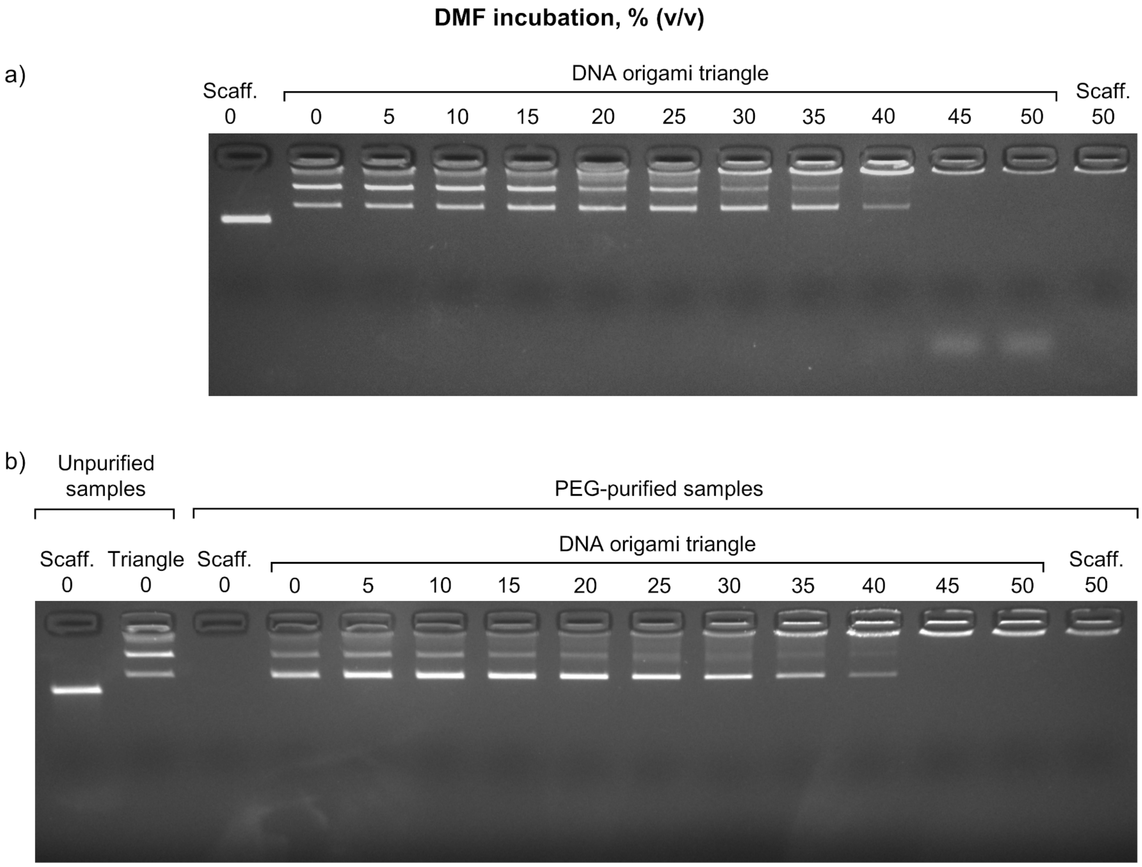

Figure S4. AGE of DNA origami triangles exposed to dimethylformamide (DMF) for 24 h at room temperature a) before and b) after removing DMF by PEG precipitation. The p7249 scaffold concentration is 10 nM and the DNA origami concentration is 5 nM in the gel.

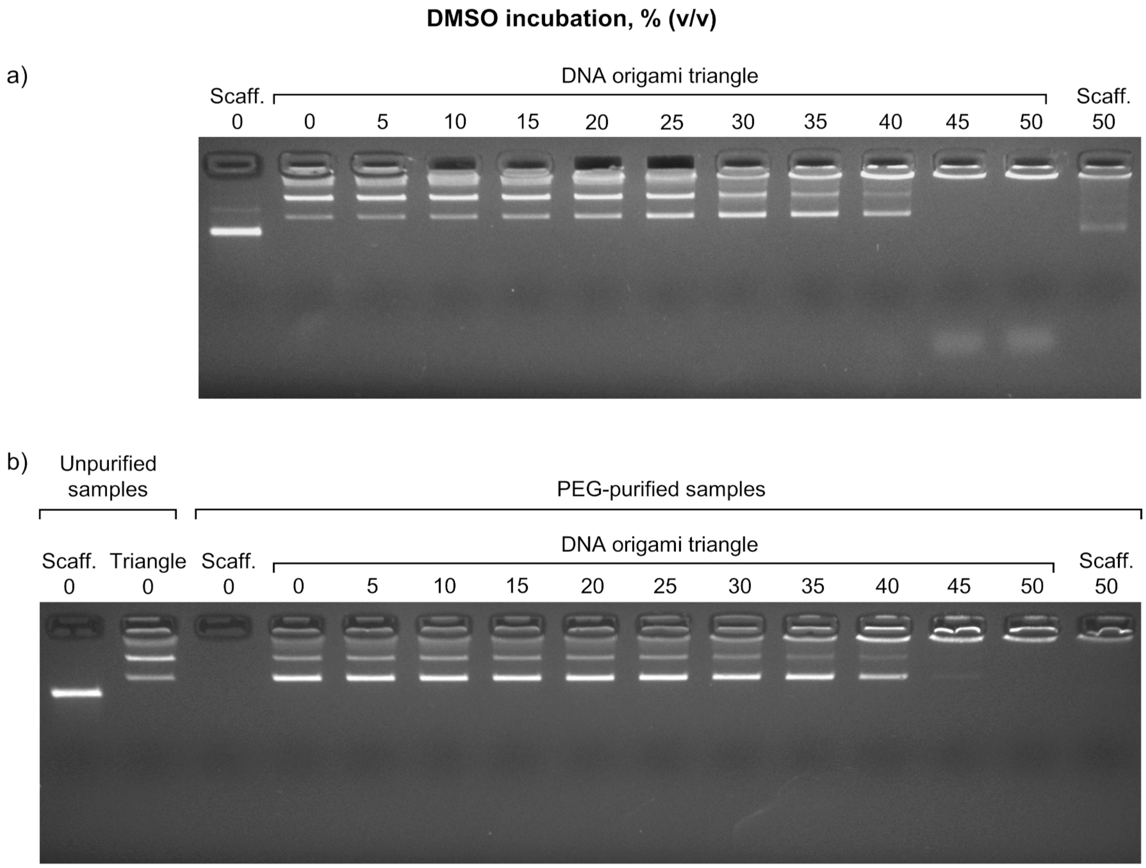

Figure S5. AGE of DNA origami triangles exposed to dimethyl sulfoxide (DMSO) for 24 h at room temperature a) before and b) after removing DMSO by PEG precipitation. The p7249 scaffold concentration is 10 nM and the DNA origami concentration is 5 nM in the gel.

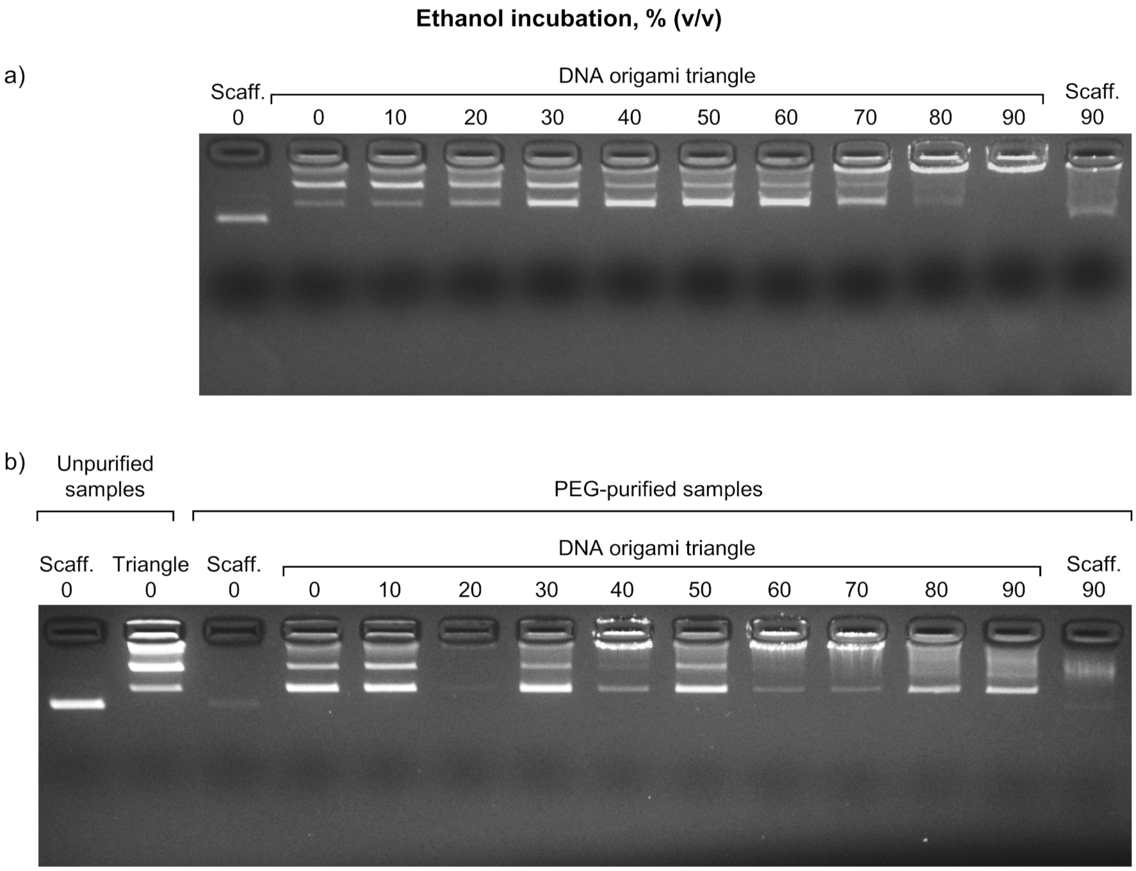

Figure S6. AGE of DNA origami triangles exposed to ethanol for 24 h at room temperature a) before and b) after removing ethanol by PEG precipitation. The p7249 scaffold concentration is 10 nM and the DNA origami concentration is 5 nM in the gel.

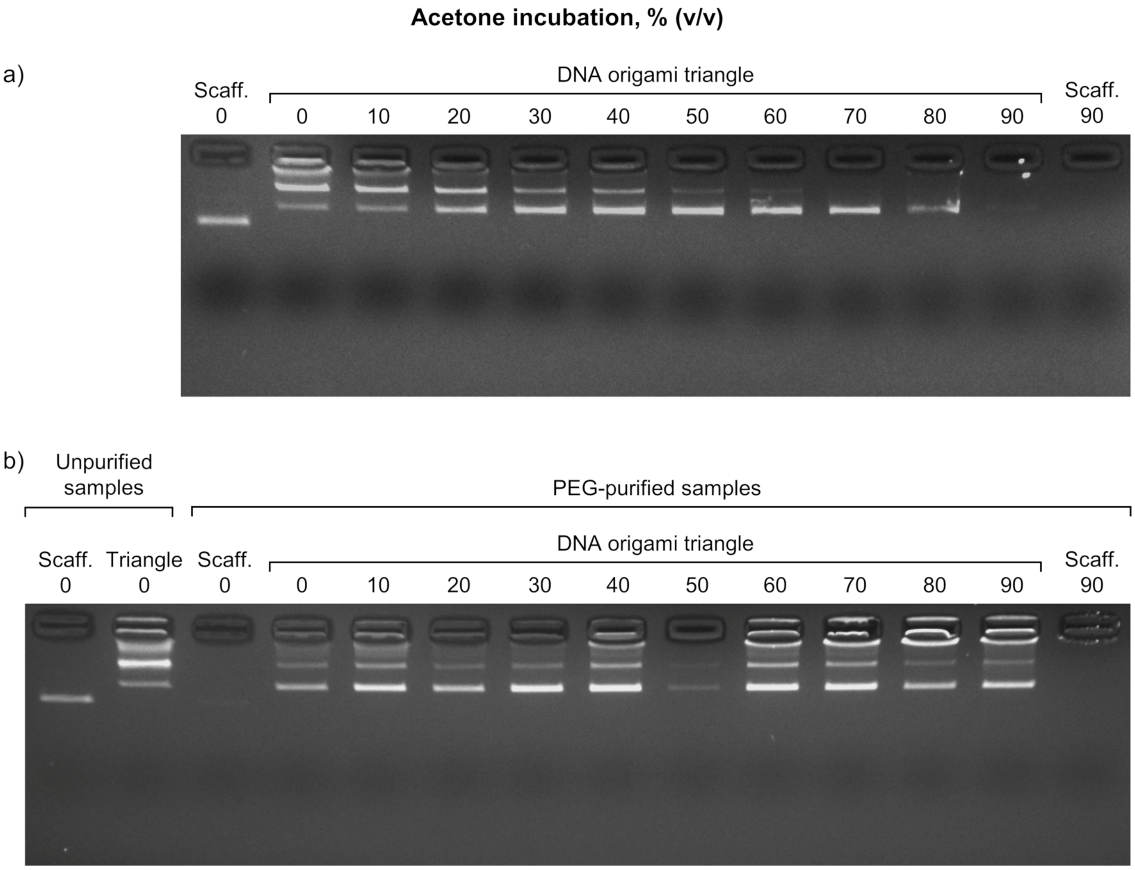

Figure S7. AGE of DNA origami triangles exposed to acetone for 24 h at room temperature a) before and b) after removing acetone by PEG precipitation. The p7249 scaffold concentration is 10 nM and the DNA origami concentration is 5 nM in the gel.

2.2. 6HB in folding buffer

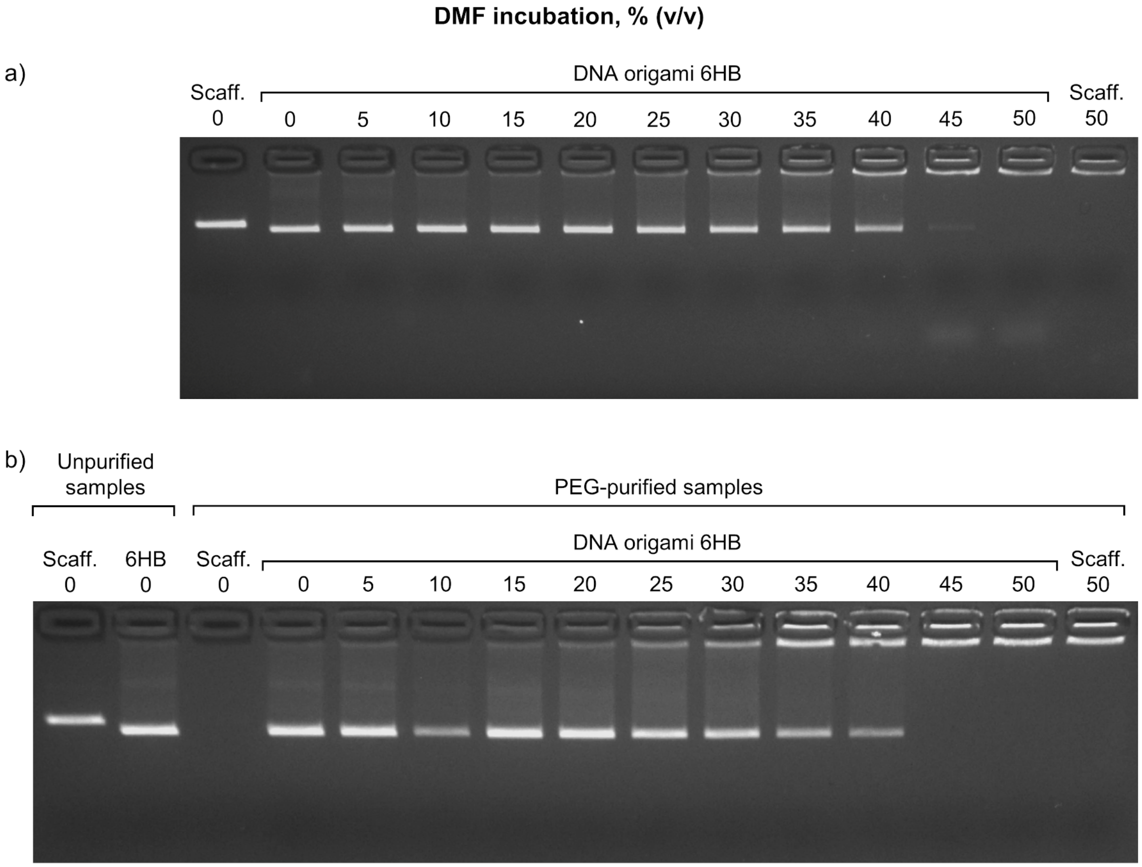

Figure S8. AGE of 6HB exposed to DMF for 24 h at room temperature a) before and b) after removing DMF by PEG precipitation. The p7249 scaffold concentration is 10 nM and the DNA origami concentration is 5 nM in the gel.

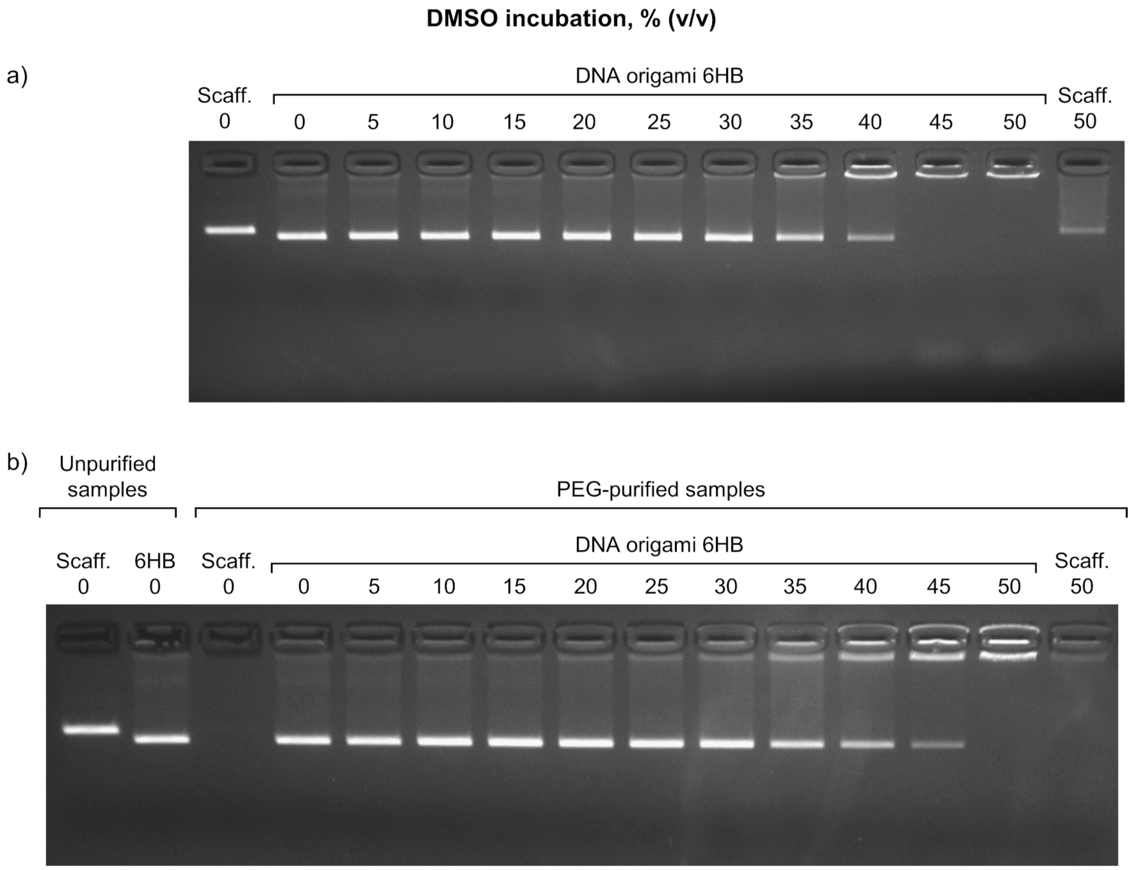

Figure S9. AGE of 6HB exposed to DMSO for 24 h at room temperature a) before and b) after removing DMSO by PEG precipitation. The p7249 scaffold concentration is 10 nM and the DNA origami concentration is 5 nM in the gel.

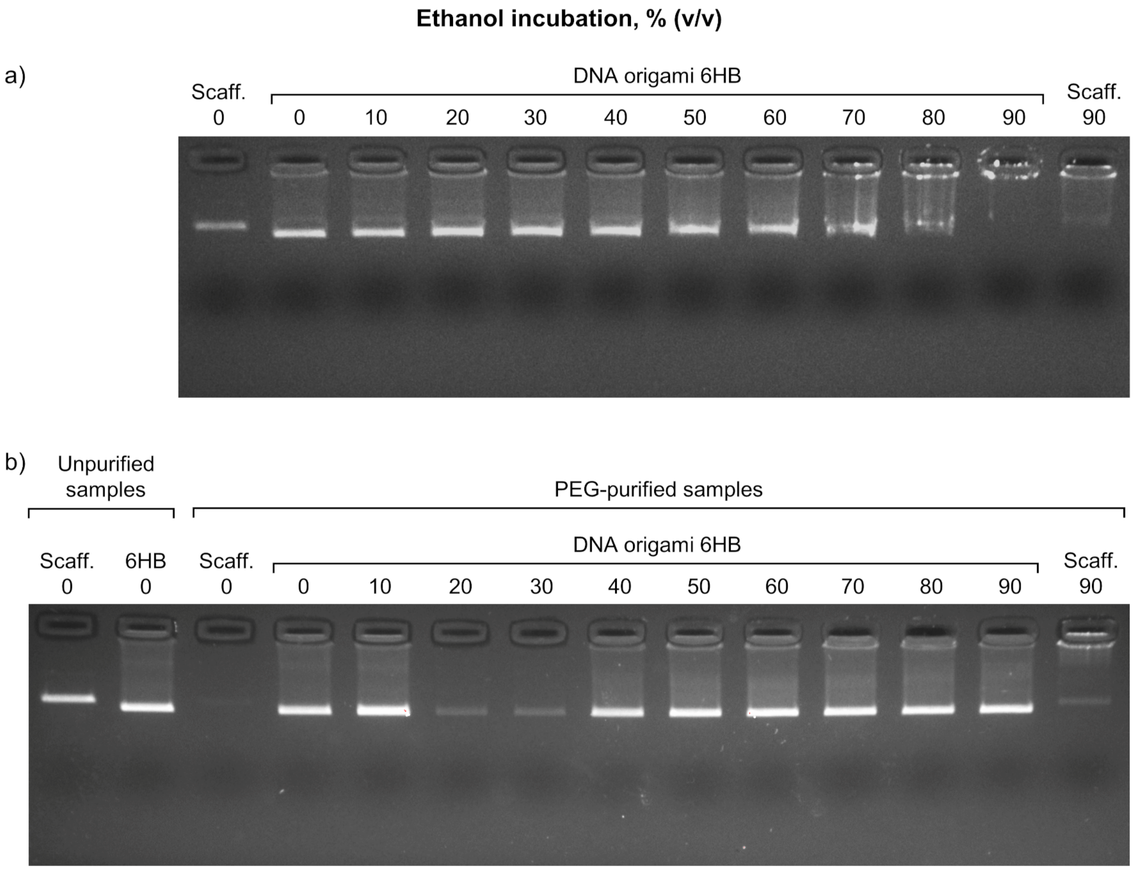

Figure S10. AGE of 6HB exposed to ethanol for 24 h at room temperature a) before and b) after removing ethanol by PEG precipitation. The p7249 scaffold concentration is 10 nM and the DNA origami concentration is 5 nM in the gel.

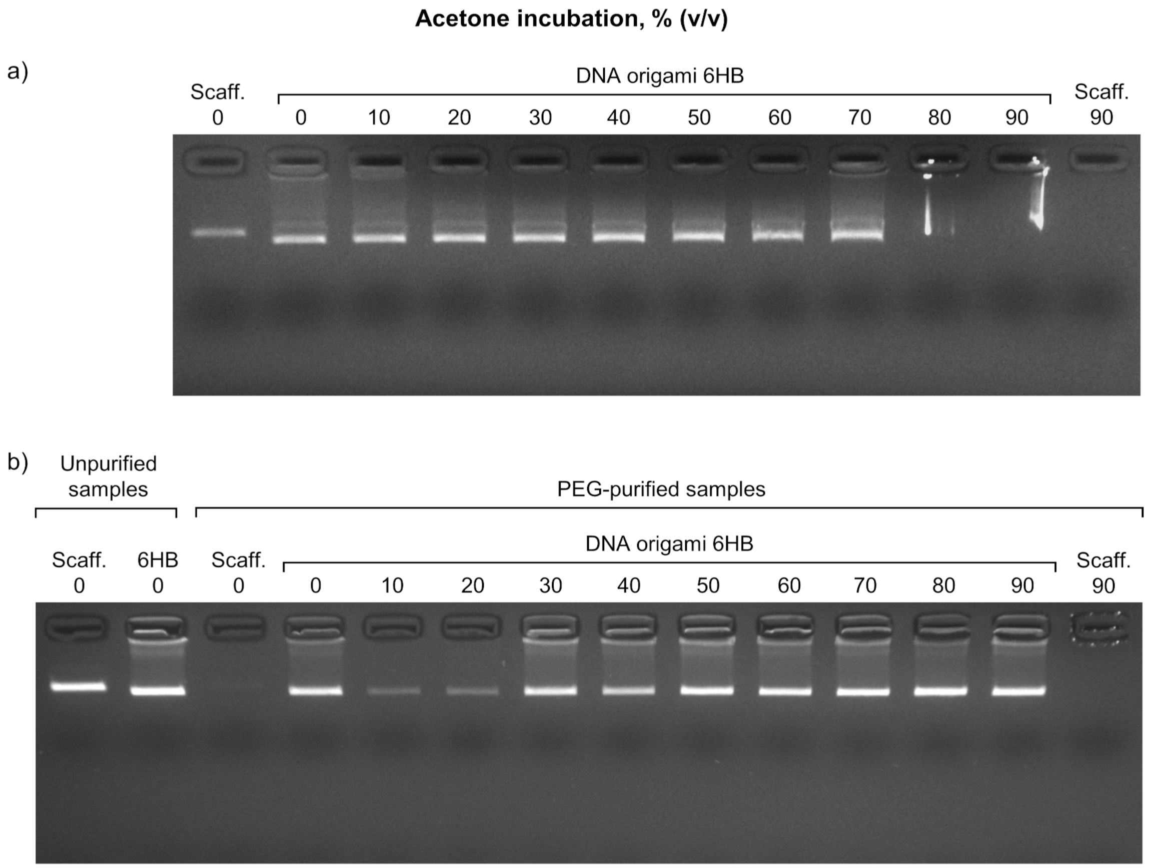

Figure S11. AGE of 6HB exposed to acetone for 24 h at room temperature a) before and b) after removing acetone by PEG precipitation. The p7249 scaffold concentration is 10 nM and the DNA origami concentration is 5 nM in the gel.

2.3. 24HB in folding buffer

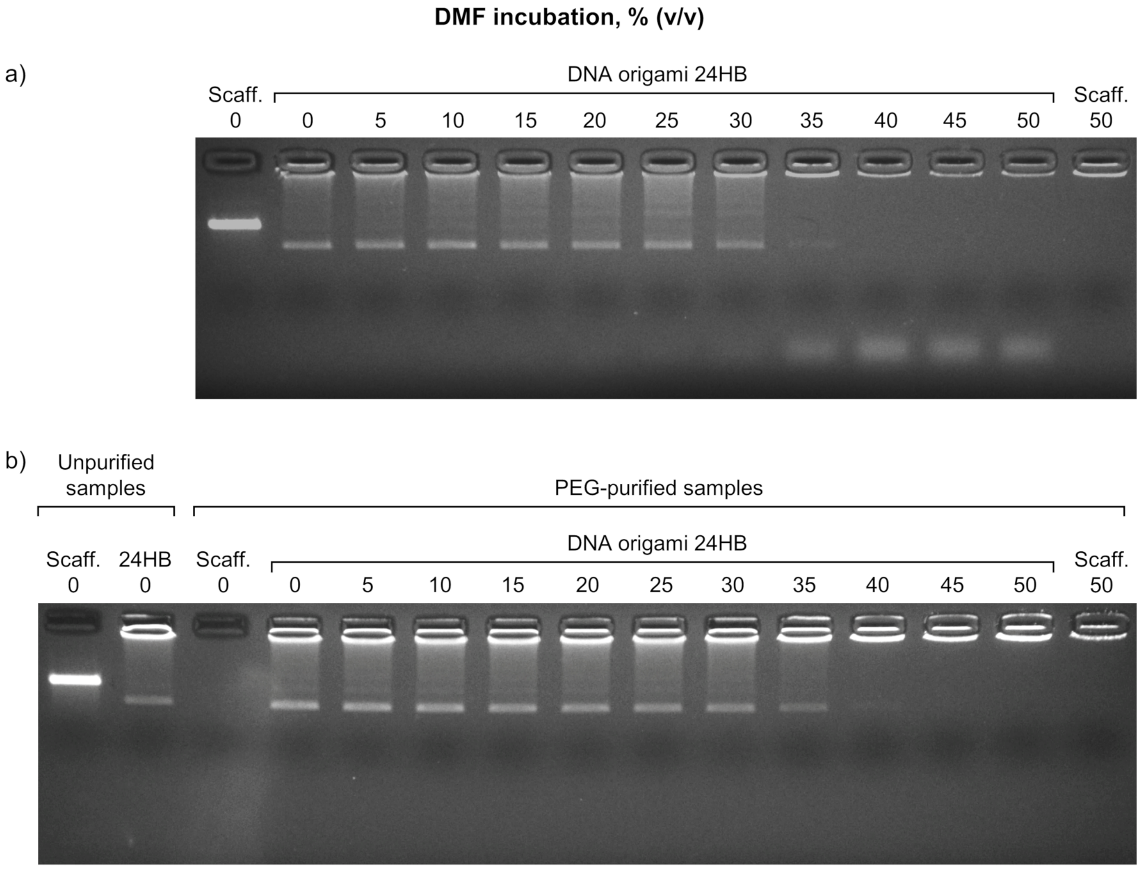

Figure S12. AGE of 24HB exposed to DMF for 24 h at room temperature a) before and b) after removing DMF by PEG precipitation. The p7560 scaffold concentration is 10 nM and the DNA origami concentration is 5 nM in the gel.

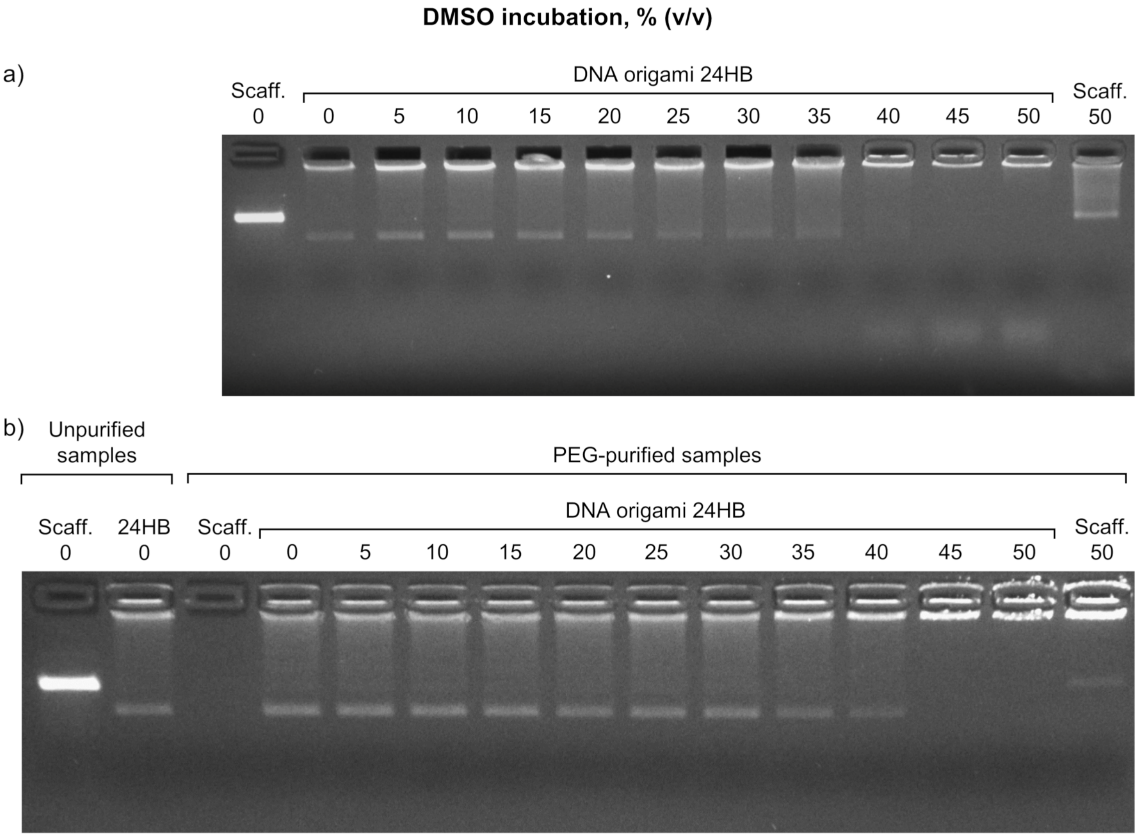

Figure S13. AGE of 24HB exposed to DMSO for 24 h at room temperature a) before and b) after removing DMSO by PEG precipitation. The p7560 scaffold concentration is 10 nM and the DNA origami concentration is 5 nM in the gel.

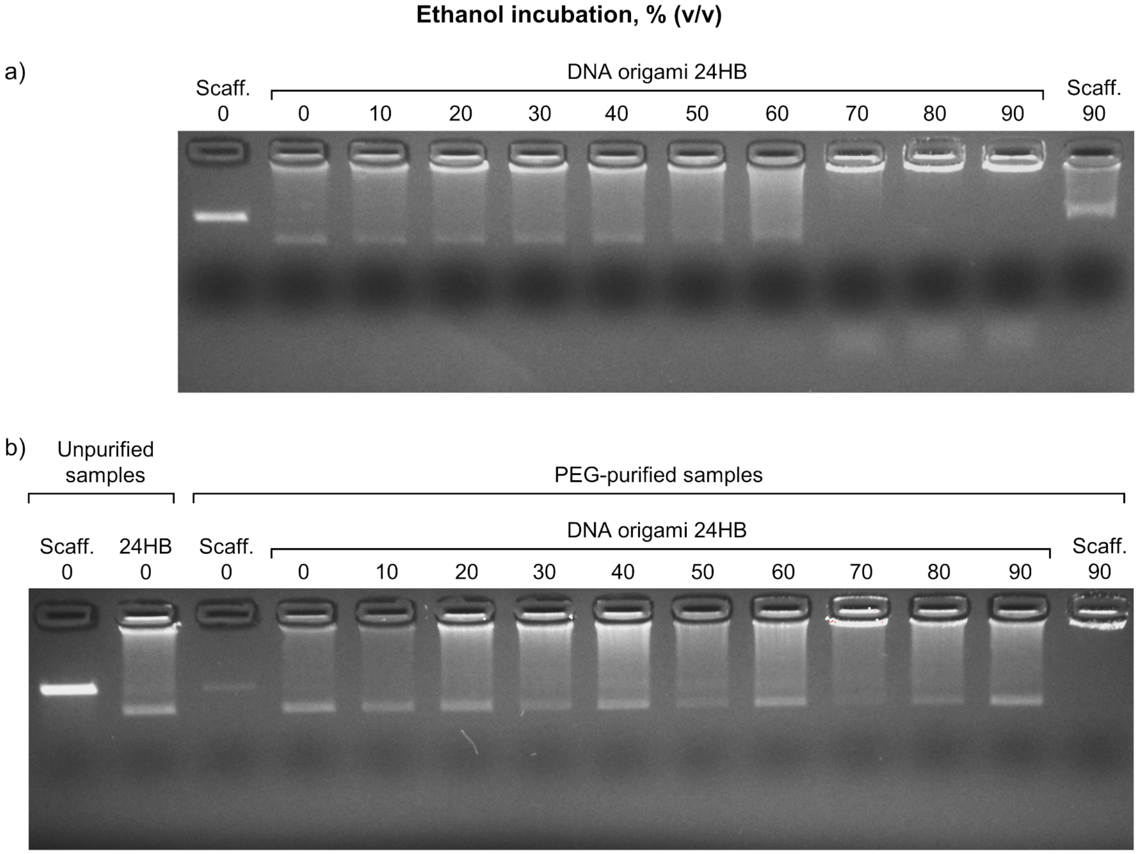

Figure S14. AGE of 24HB exposed to ethanol for 24 h at room temperature a) before and b) after removing ethanol by PEG precipitation. The p7560 scaffold concentration is 10 nM and the DNA origami concentration is 5 nM in the gel.

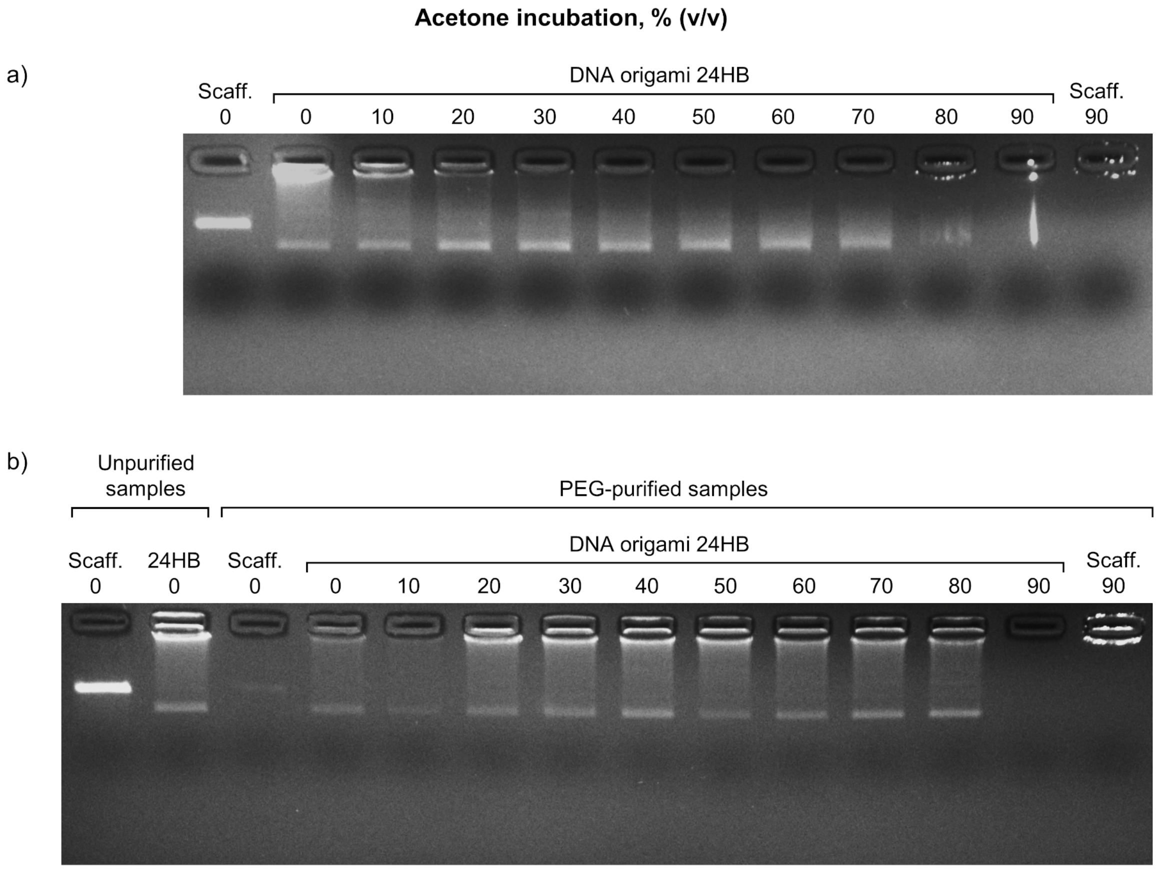

Figure S15. AGE of 24HB exposed to acetone for 24 h at room temperature a) before and b) after removing acetone by PEG precipitation. The p7560 scaffold concentration is 10 nM and the DNA origami concentration is 5 nM in the gel.

3. Additional AFM images in folding buffer

3.1. DNA origami triangle in folding buffer exposed to DMF

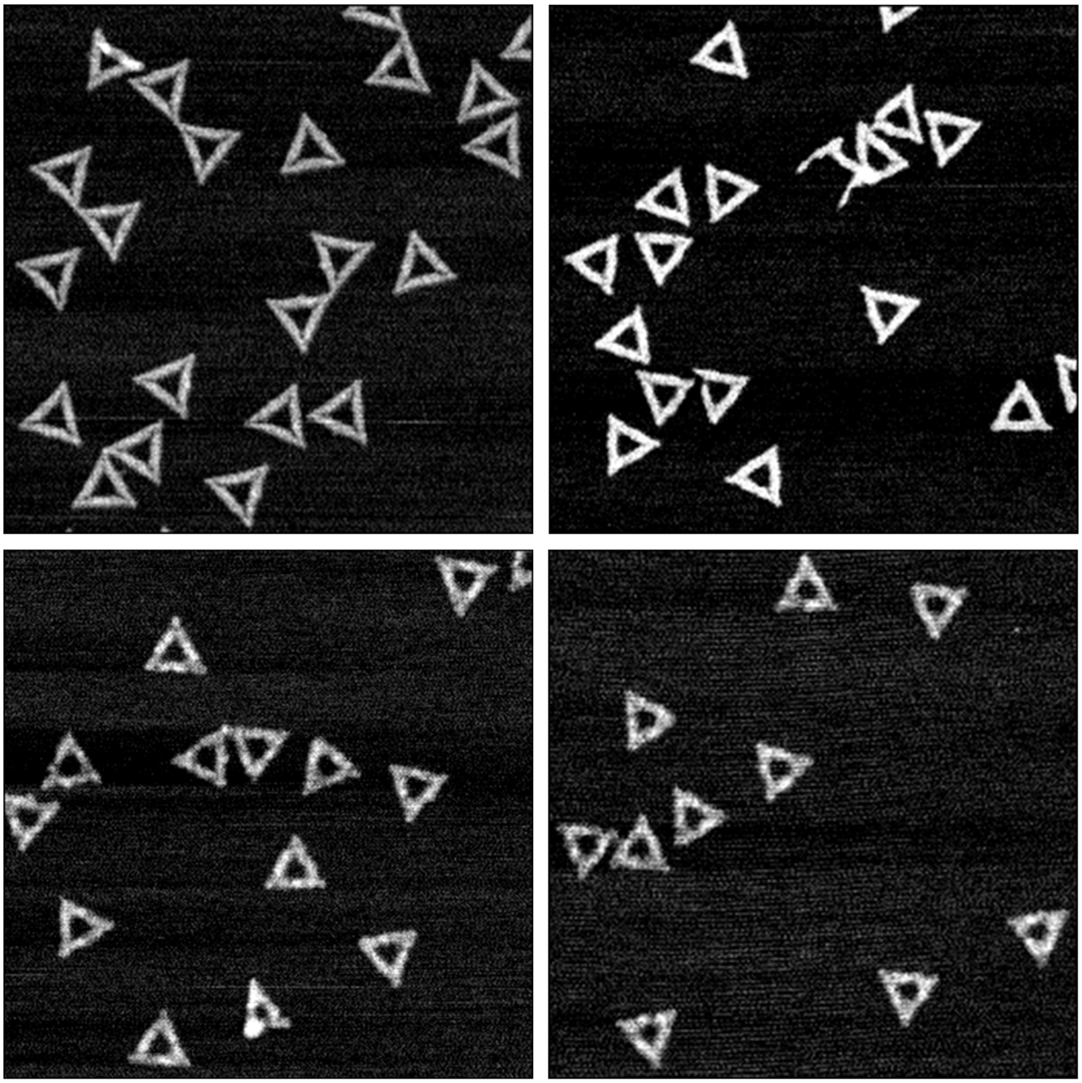

Figure S16. AFM images (1 μm × 1 μm) of DNA origami triangles in FOB exposed to 35% (v/v) DMF for 24 h at room temperature. DMF was removed by PEG precipitation before the AFM sample preparation.

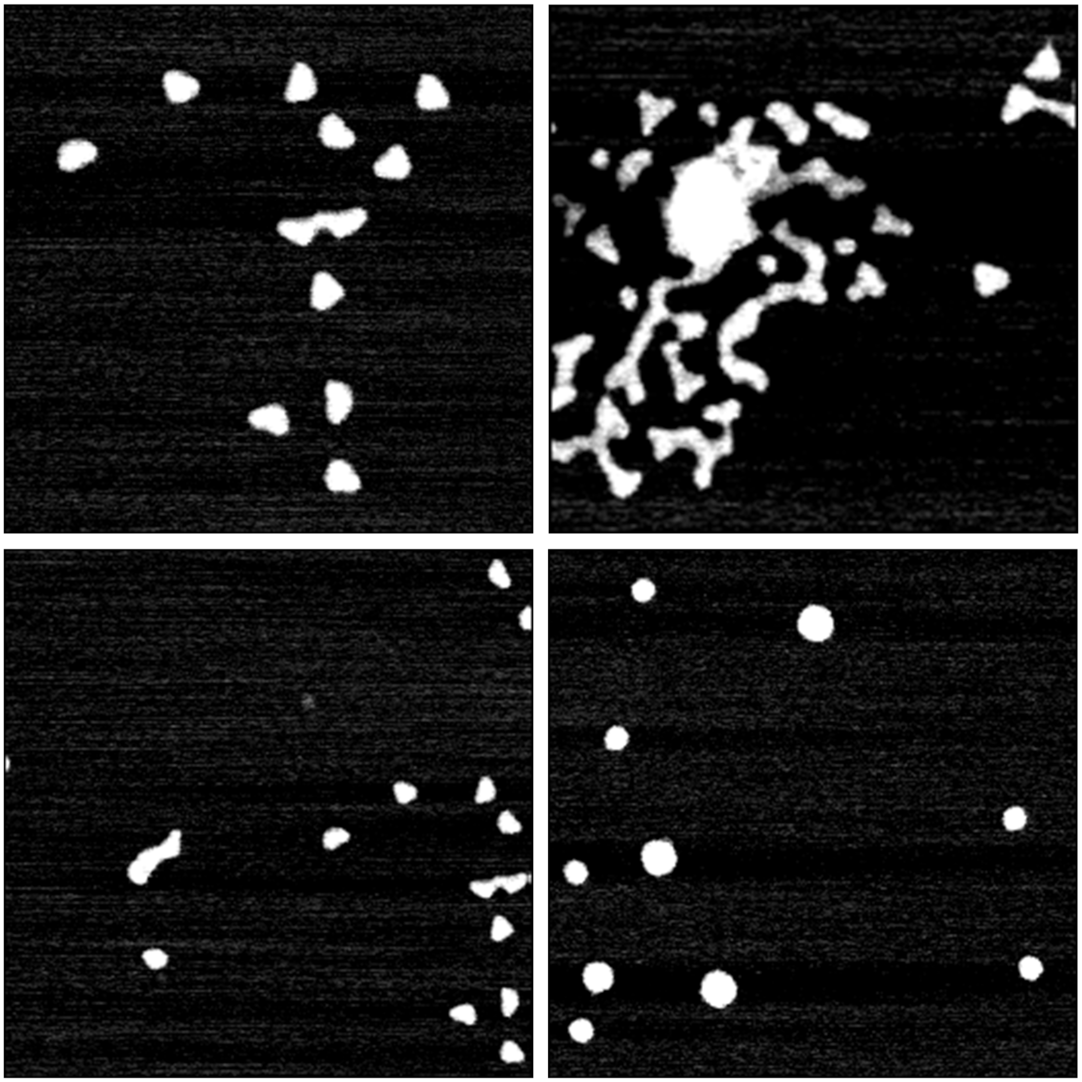

Figure S17. AFM images (1 μm × 1 μm) of DNA origami triangles in FOB exposed to 40% (v/v) DMF for 24 h at room temperature. DMF was removed by PEG precipitation before the AFM sample preparation.

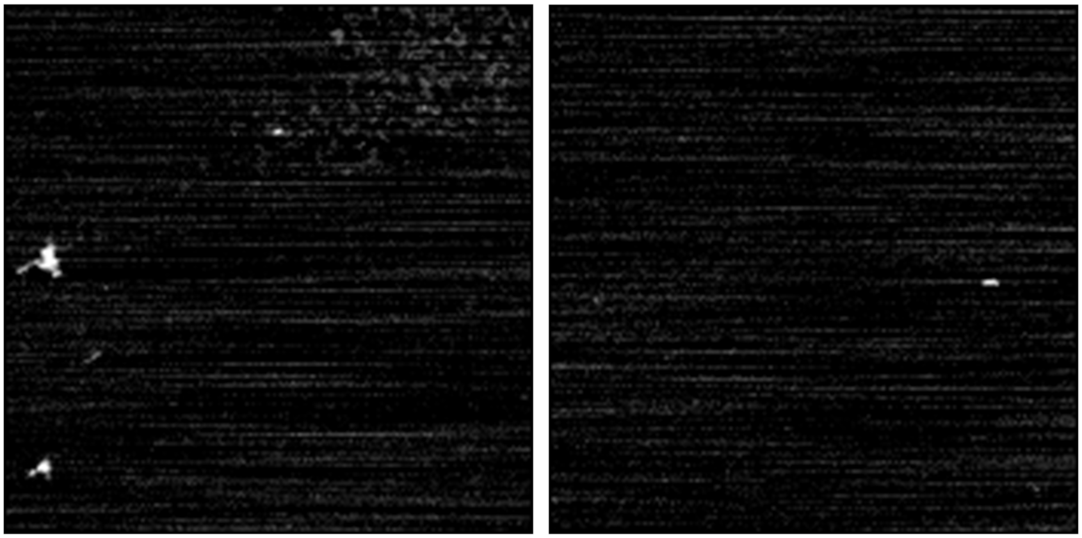

Figure S18. AFM images (1 μm × 1 μm) of DNA origami triangles in in FOB exposed to 45% (v/v) DMF for 24 h at room temperature. DMF was removed by PEG precipitation before the AFM sample preparation.

3.2. DNA origami triangle in folding buffer exposed to DMSO

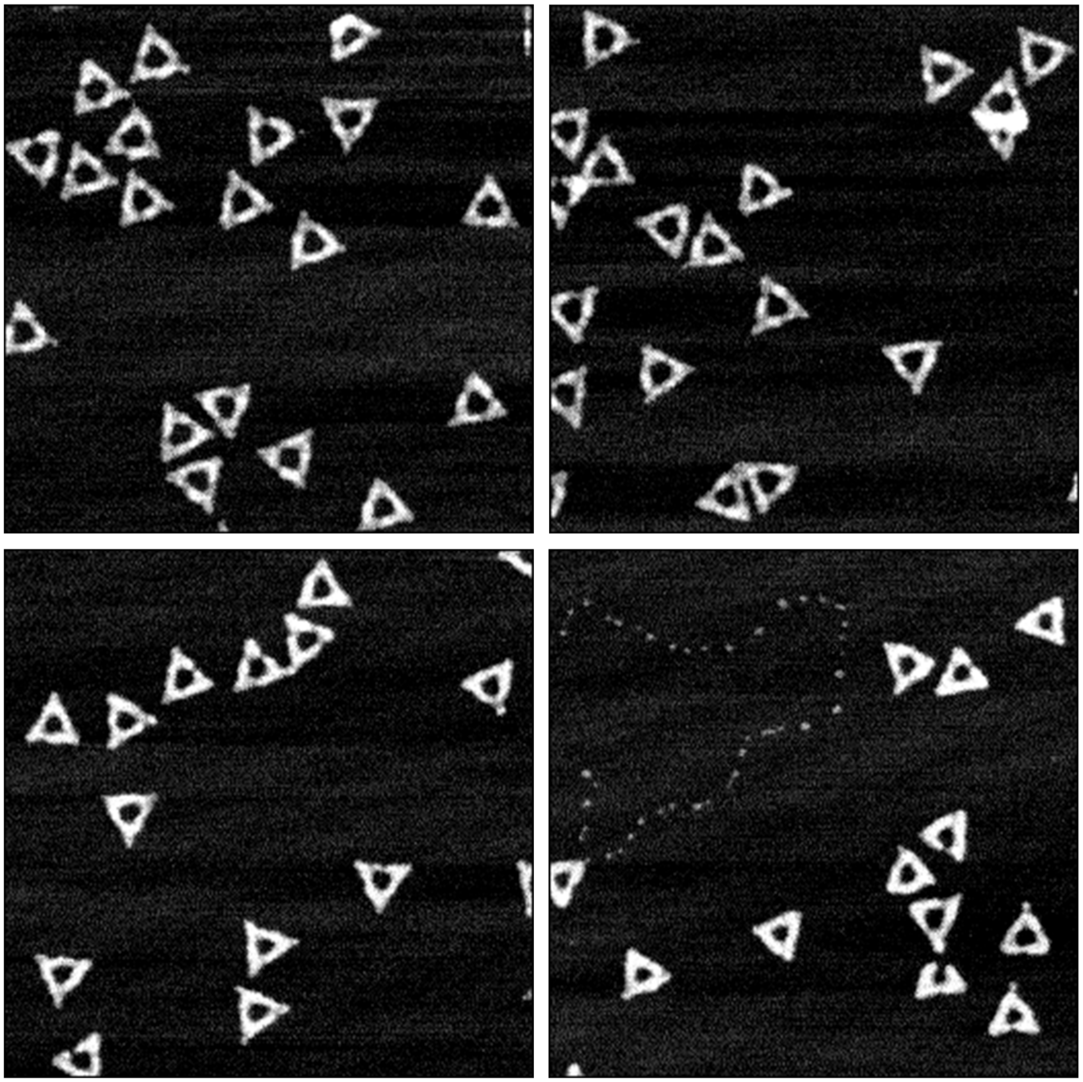

Figure S19. AFM images (1 μm $\times$ 1 μm) of DNA origami triangles in FOB exposed to 35% (v/v) DMSO for 24 h at room temperature. DMF was removed by PEG precipitation before the AFM sample preparation.

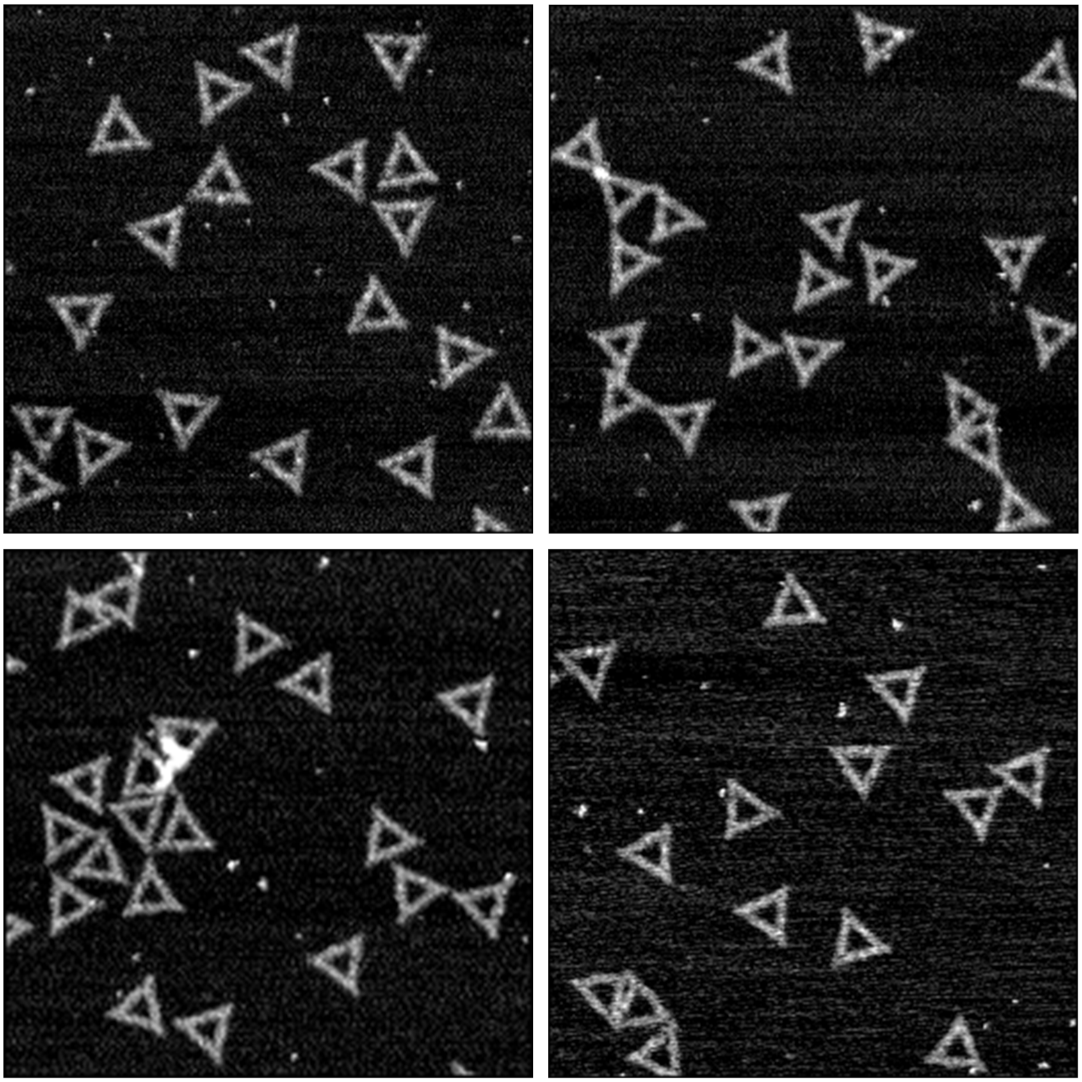

Figure S20. AFM images (1 μm × 1 μm) of DNA origami triangles in FOB exposed to 40% (v/v) DMSO for 24 h at room temperature. DMF was removed by PEG precipitation before the AFM sample preparation.

3.3. DNA origami triangle in folding buffer exposed to ethanol

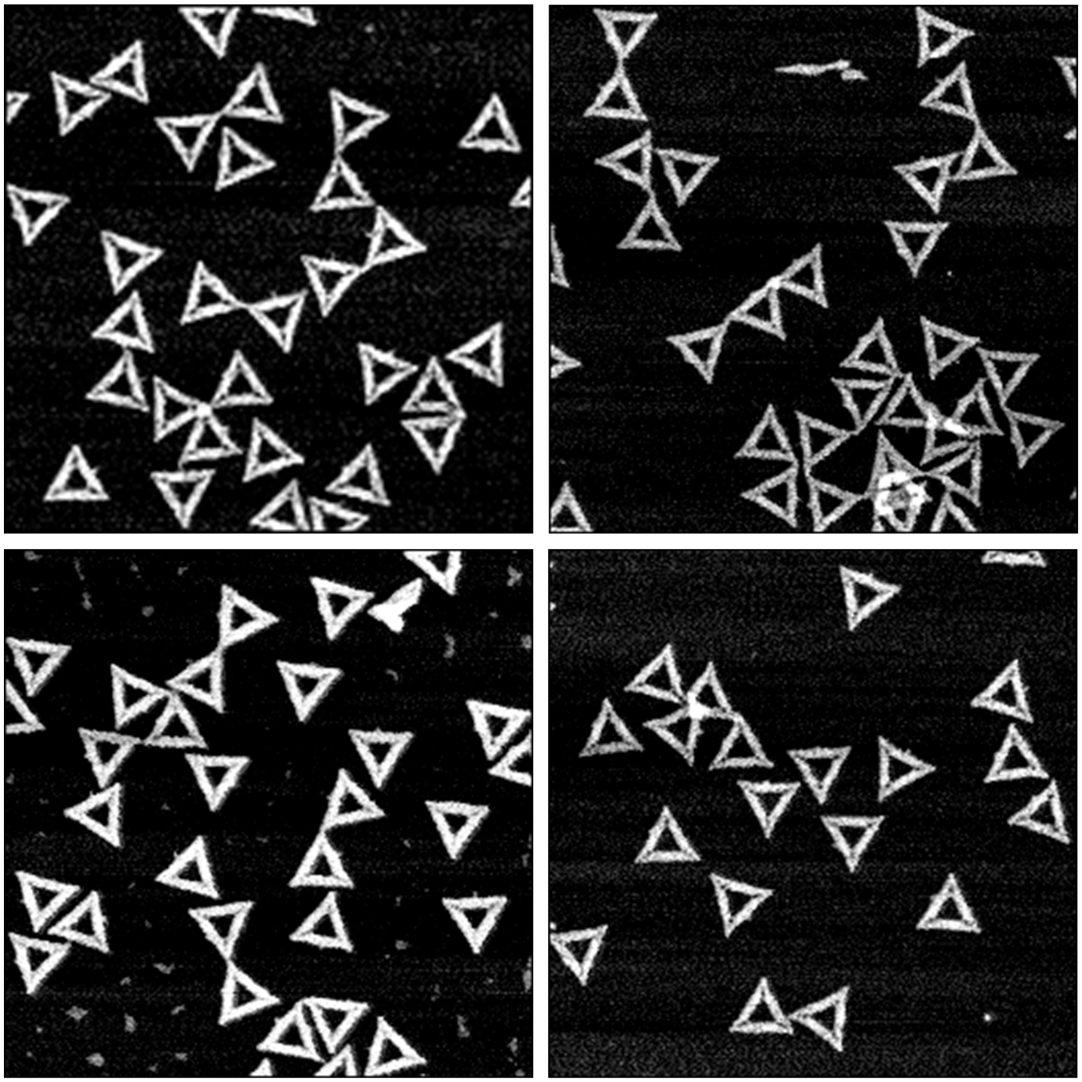

Figure S21. AFM images (1 μm × 1 μm) of DNA origami triangles in FOB exposed to 10% (v/v) ethanol for 24 h at room temperature. Ethanol was removed by PEG precipitation before the AFM sample preparation.

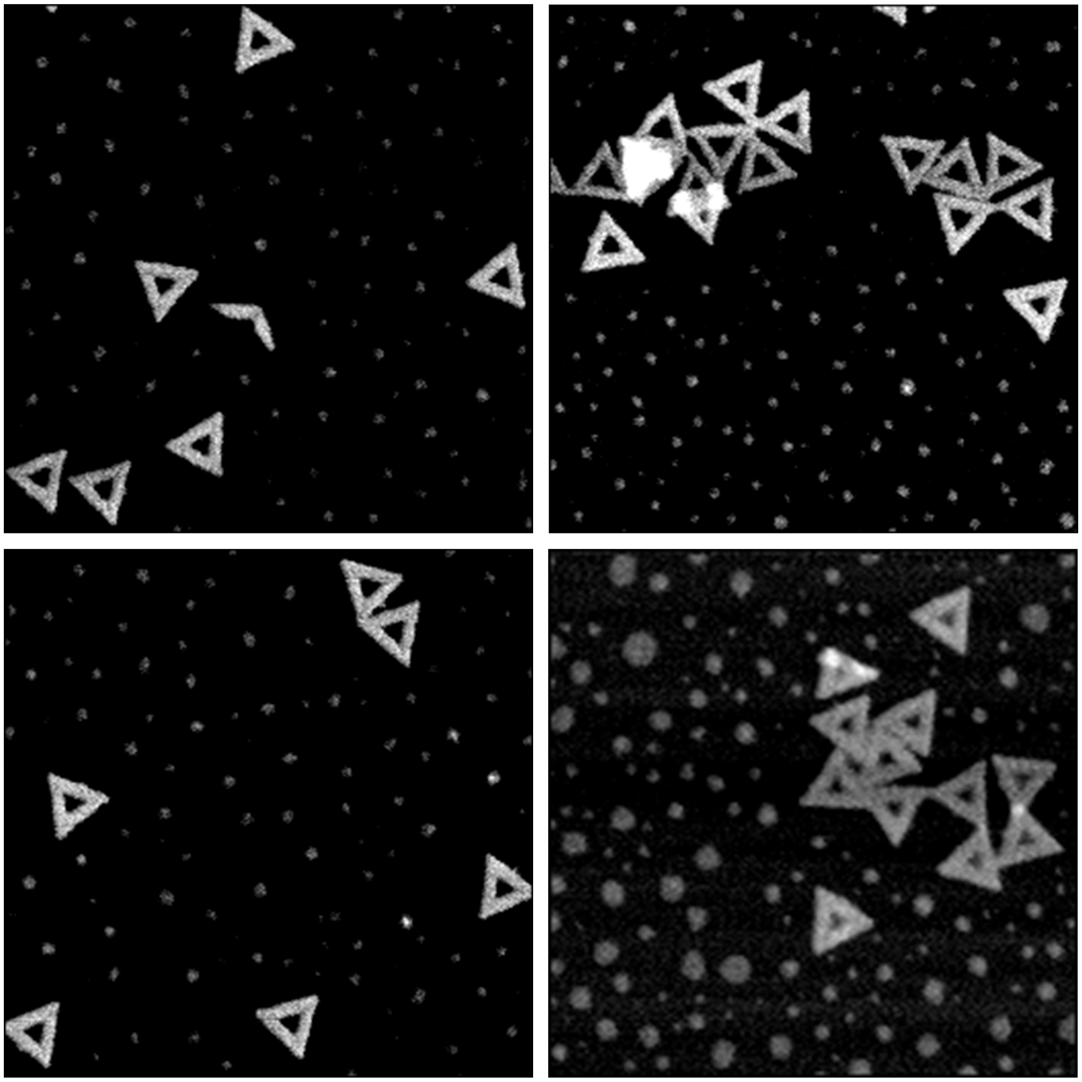

Figure S22. AFM images (1 μm × 1 μm) of DNA origami triangles in FOB exposed to 40% (v/v) ethanol for 24 h at room temperature. Ethanol was removed by PEG precipitation before the AFM sample preparation.

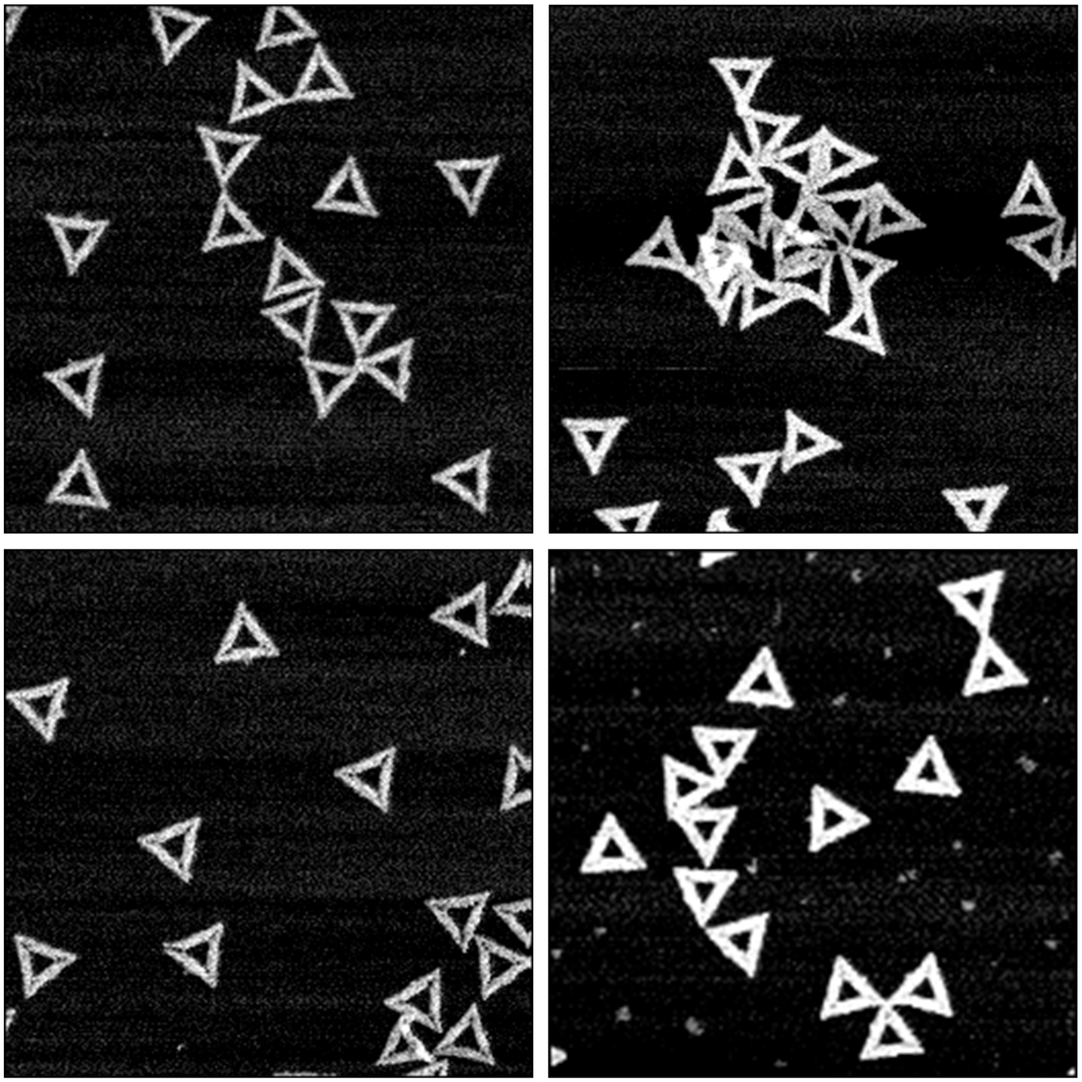

Figure S23. AFM images (1 μm × 1 μm) of DNA origami triangles in FOB exposed to 90% (v/v) ethanol for 24 h at room temperature. Ethanol was removed by PEG precipitation before the AFM sample preparation.

3.4. DNA origami triangle in folding buffer exposed to acetone

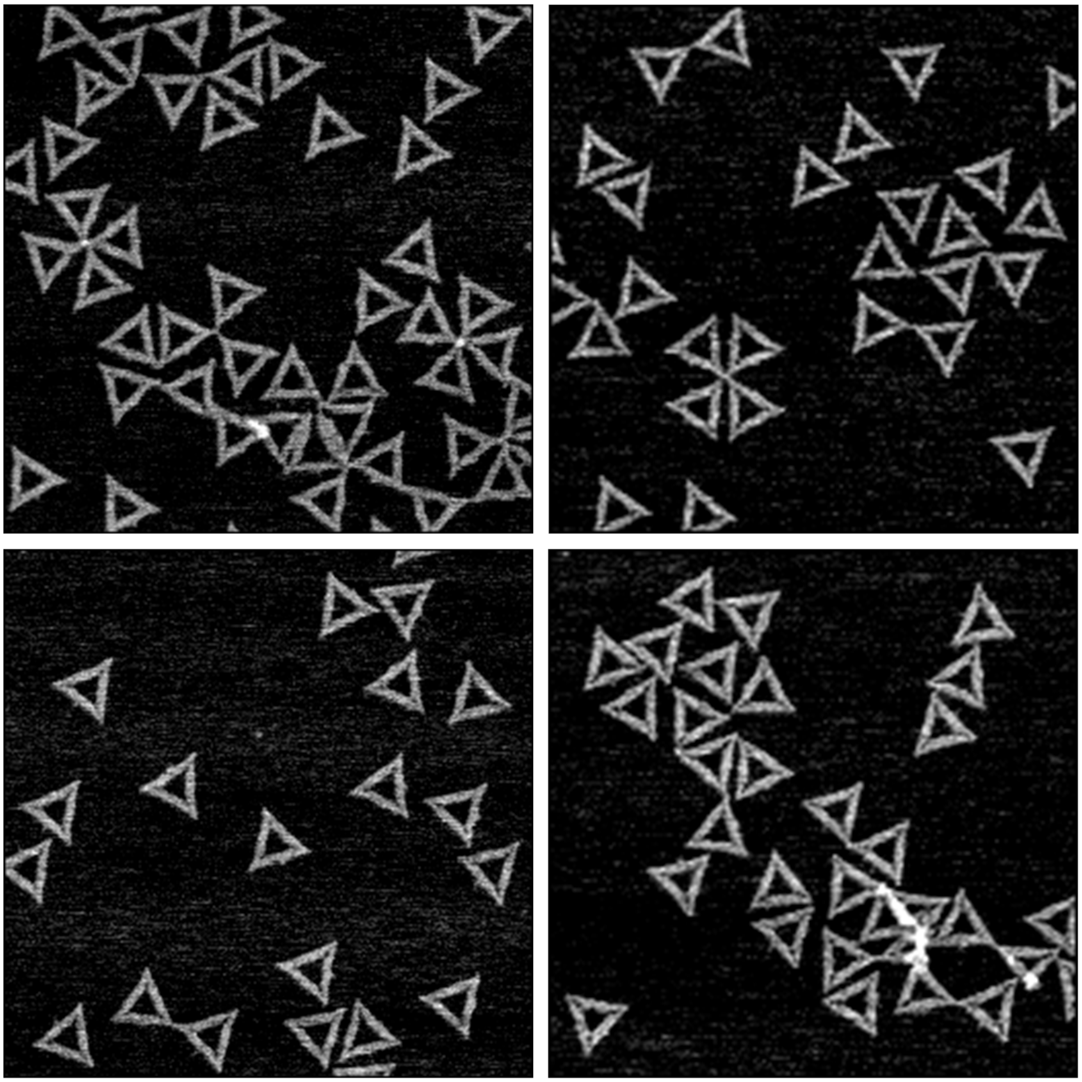

Figure S24. AFM images (1 μm × 1 μm) of DNA origami triangles in FOB exposed to 10% (v/v) acetone for 24 h at room temperature. Acetone was removed by PEG precipitation before the AFM sample preparation.

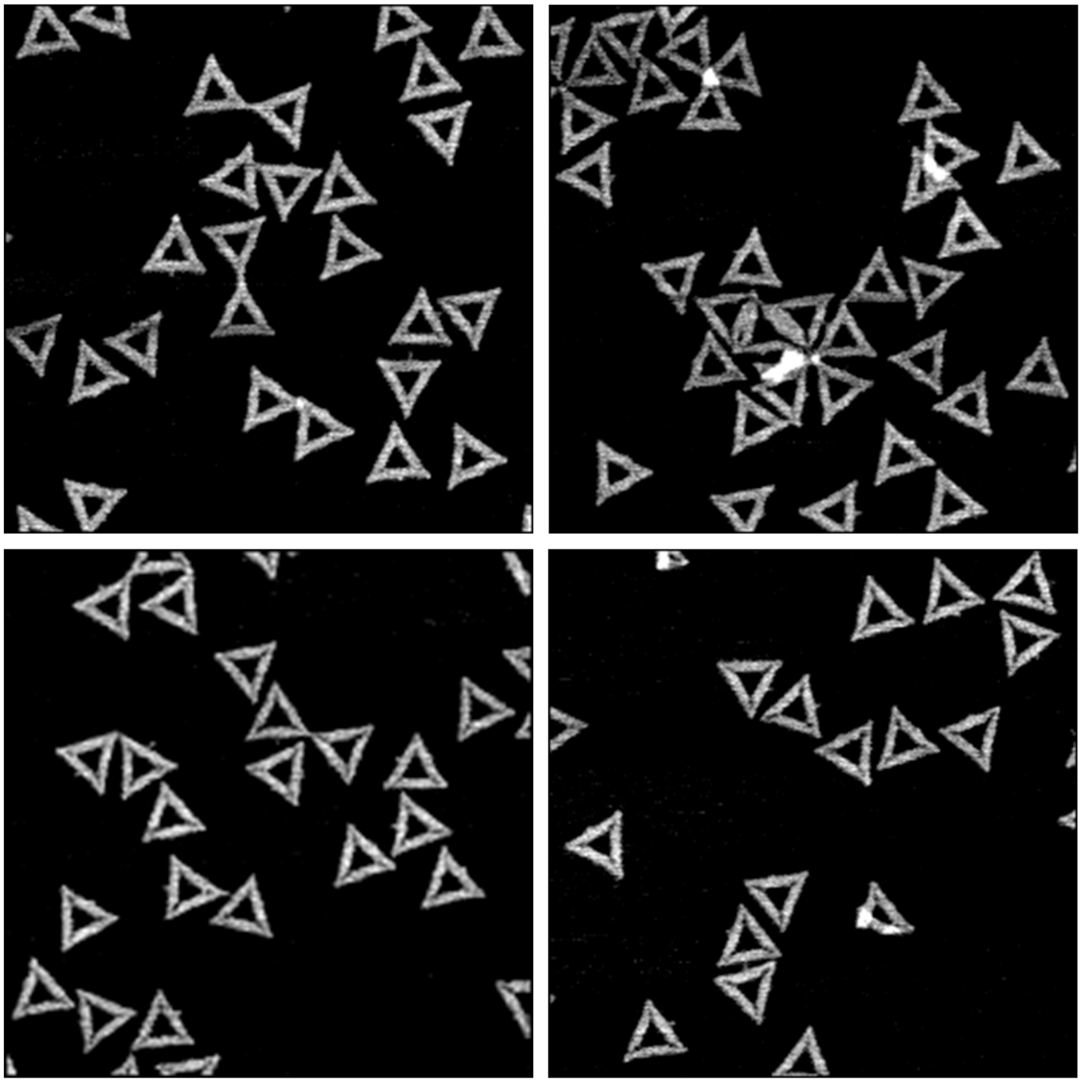

Figure S25. AFM images (1 μm × 1 μm) of DNA origami triangles in FOB exposed to 40% (v/v) acetone for 24 h at room temperature. Acetone was removed by PEG precipitation before the AFM sample preparation.

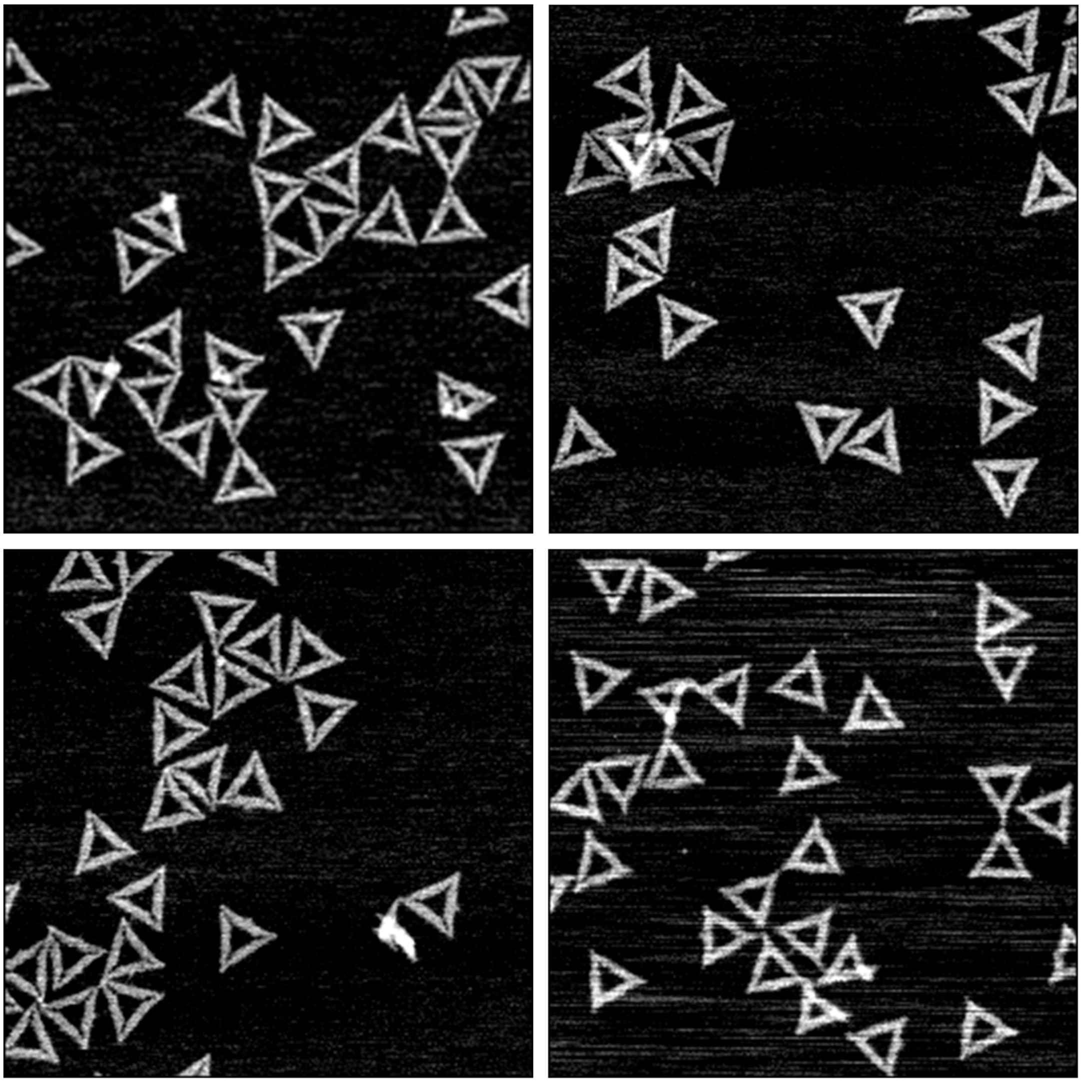

Figure S26. AFM images (1 μm × 1 μm) of DNA origami triangles in FOB exposed to 90% (v/v) acetone for 24 h at room temperature. Acetone was removed by PEG precipitation before the AFM sample preparation.

4. Additional TEM images in folding buffer

4.1. 6HB in folding buffer exposed to DMF

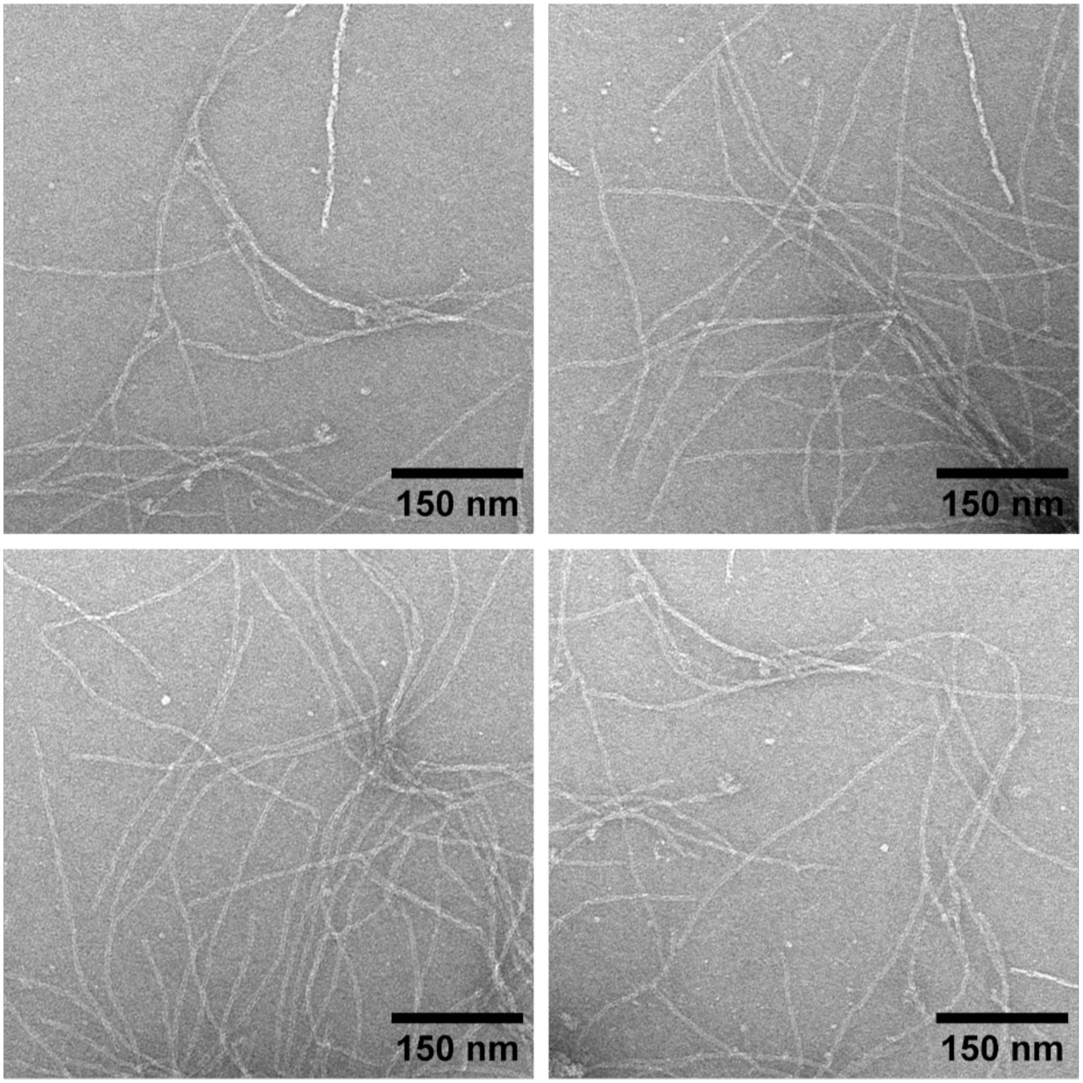

Figure S27. TEM images of 6HB in FOB exposed to 10% (v/v) DMF for 24 h at room temperature. The TEM samples are negatively stained with uranyl formate (2% (w/v)). DMF was removed by PEG precipitation before the TEM sample preparation.

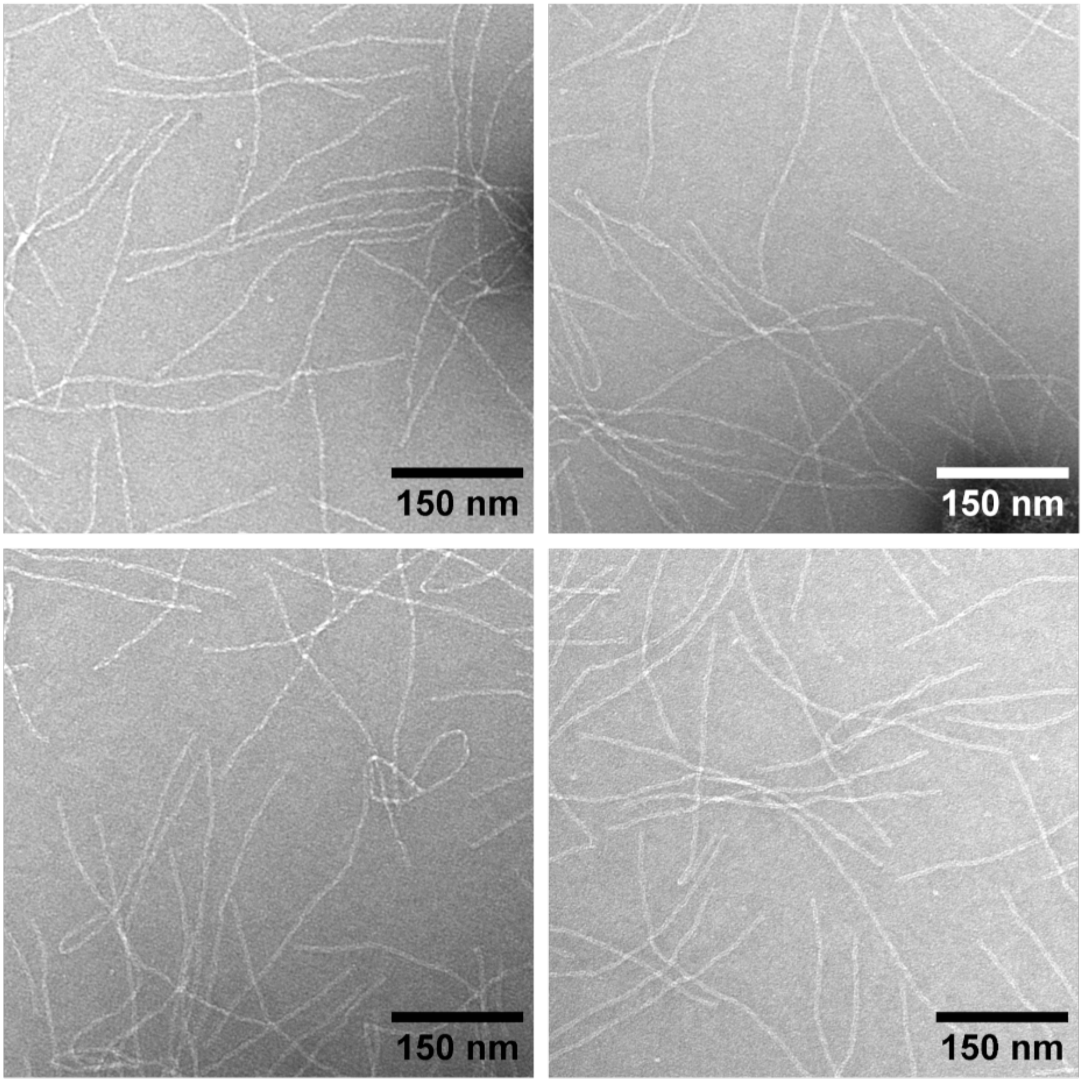

Figure S28. TEM images of 6HB in FOB exposed to 35% (v/v) DMF for 24 h at room temperature. The TEM samples are negatively stained with uranyl formate (2% (w/v)). DMF was removed by PEG precipitation before the TEM sample preparation.

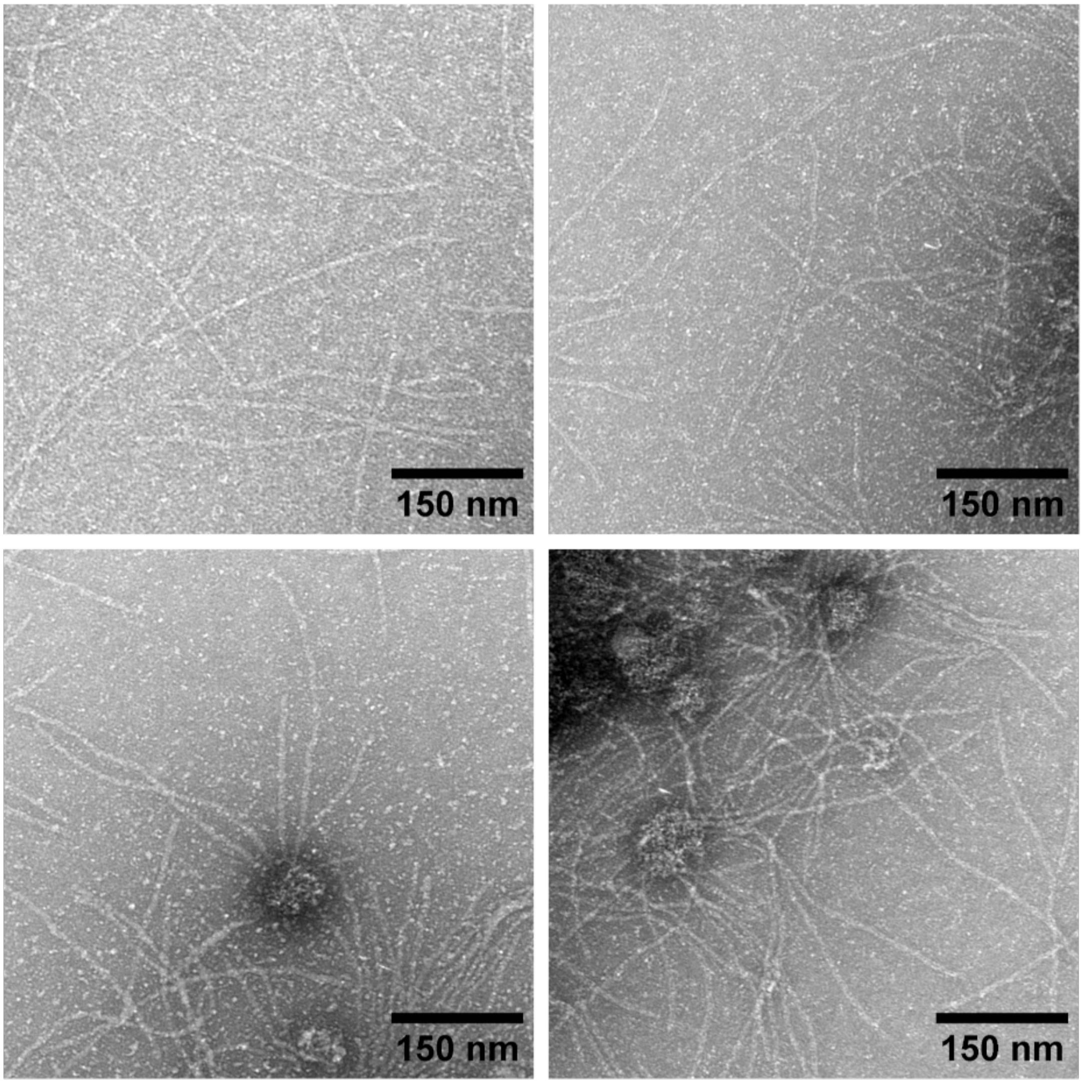

Figure S29. TEM images of 6HB in FOB exposed to 40% (v/v) DMF for 24 h at room temperature. The TEM samples are negatively stained with uranyl formate (2% (w/v)). DMF was removed by PEG precipitation before the TEM sample preparation.

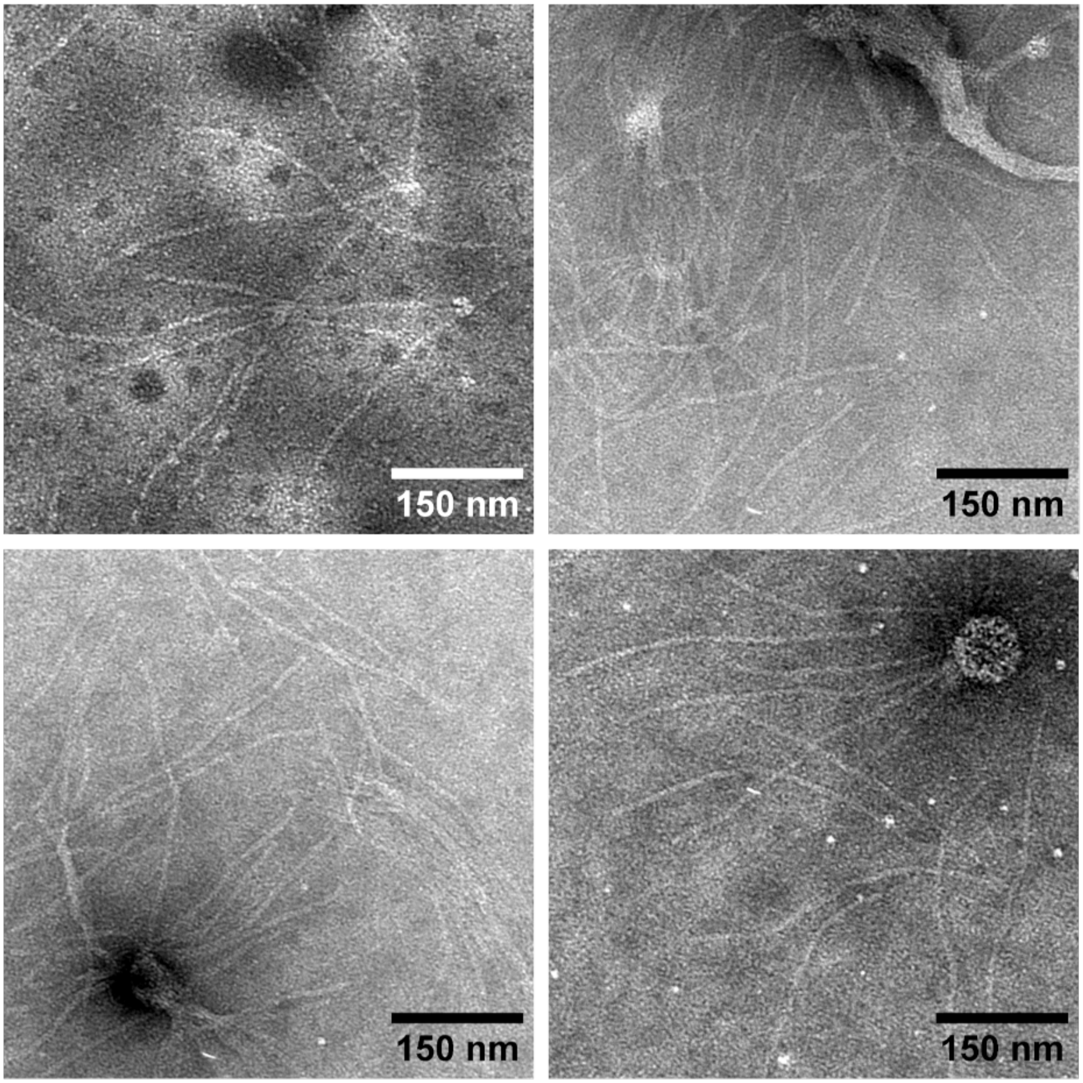

Figure S30. TEM images of 6HB in FOB exposed to 45% (v/v) DMF for 24 h at room temperature. The TEM samples are negatively stained with uranyl formate (2% (w/v)). DMF was removed by PEG precipitation before the TEM sample preparation.

4.2. 6HB in folding buffer exposed to DMSO

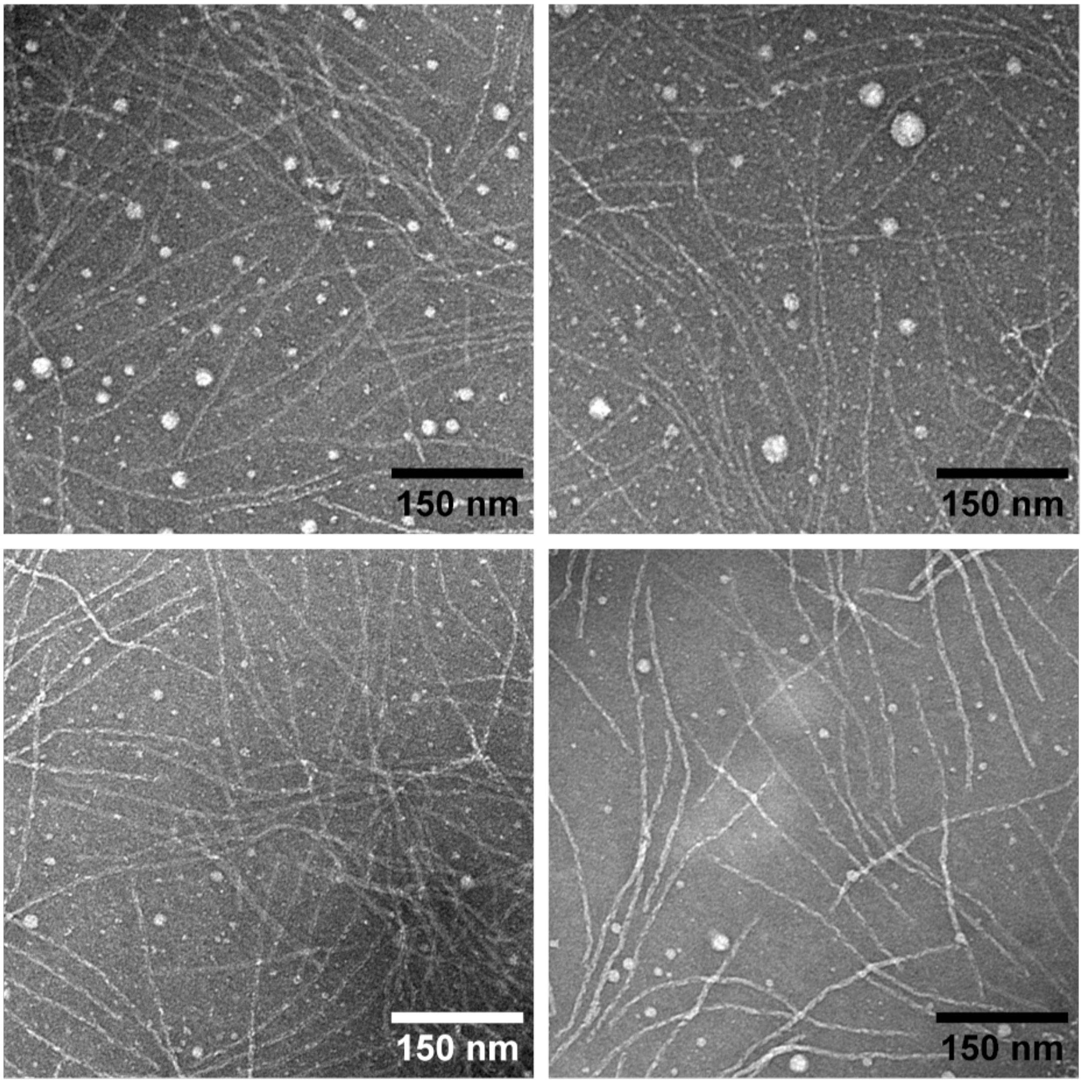

Figure S31. TEM images of 6HB in FOB exposed to 10% (v/v) DMSO for 24 h at room temperature. The TEM samples are negatively stained with uranyl formate (2% (w/v)). DMSO was removed by PEG precipitation before the TEM sample preparation.

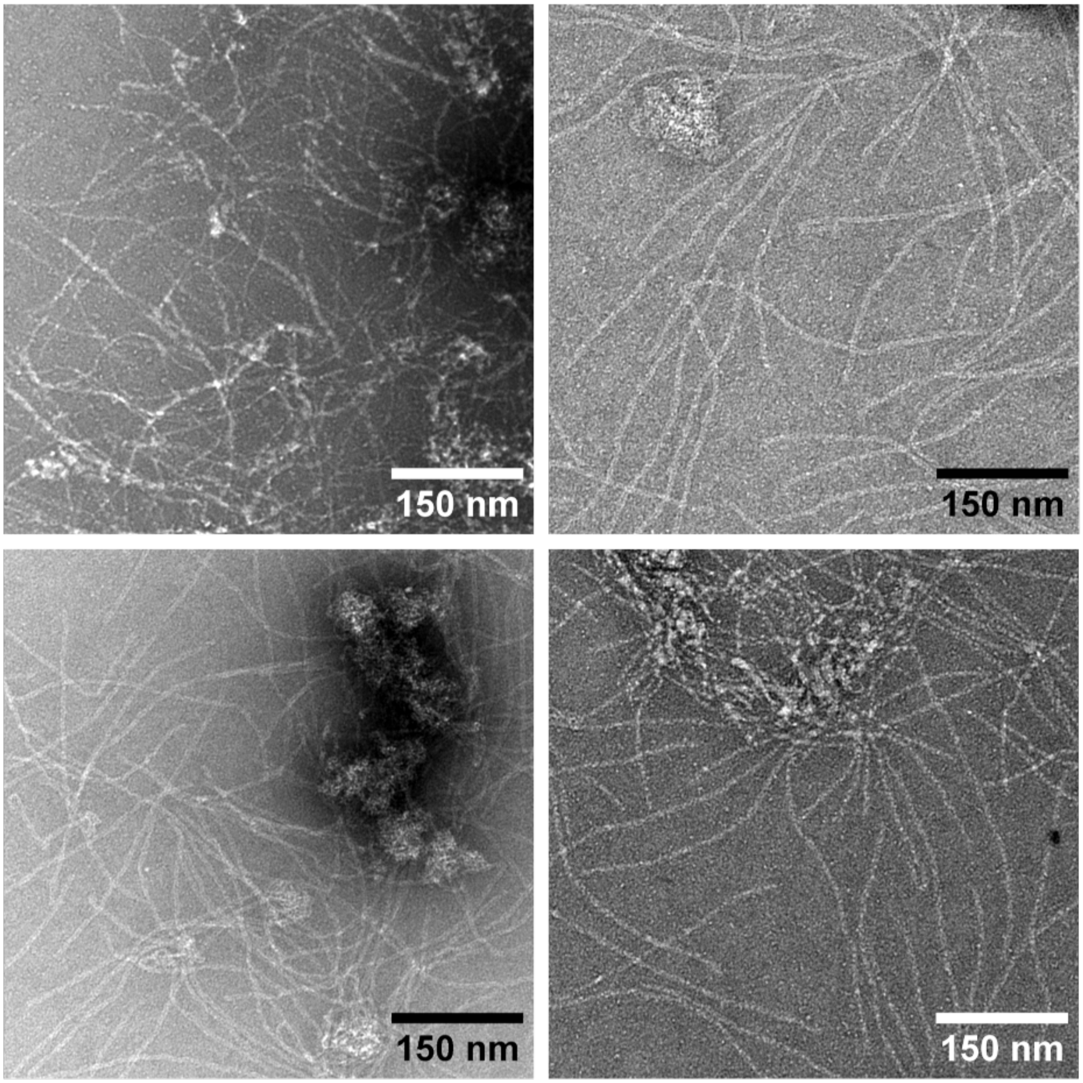

Figure S32. TEM images of 6HB in FOB exposed to 35% (v/v) DMSO for 24 h at room temperature. The TEM samples are negatively stained with uranyl formate (2% (w/v)). DMSO was removed by PEG precipitation before the TEM sample preparation.

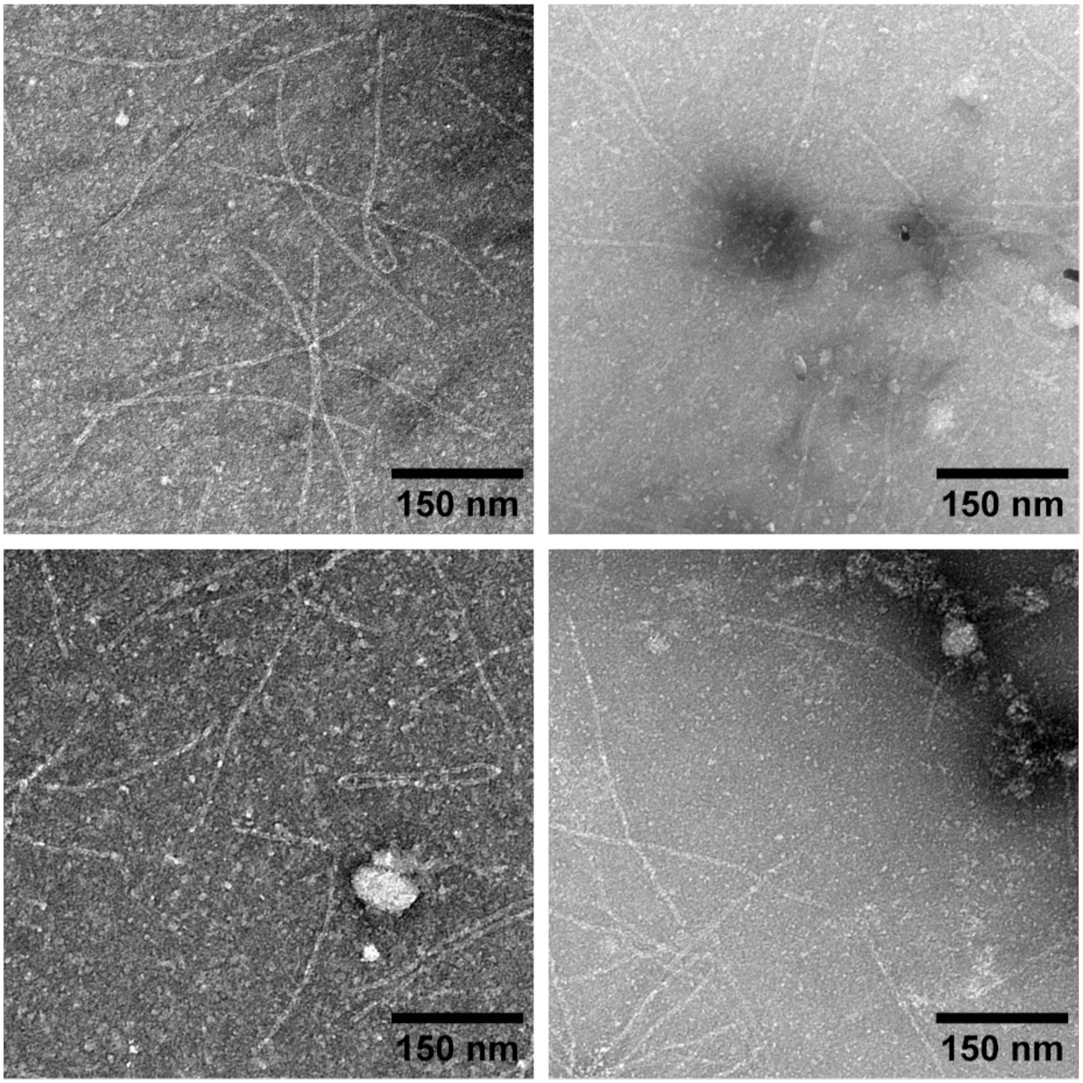

Figure S33. TEM images of 6HB in FOB exposed to 45% (v/v) DMSO for 24 h at room temperature. The TEM samples are negatively stained with uranyl formate (2% (w/v)). DMSO was removed by PEG precipitation before the TEM sample preparation.

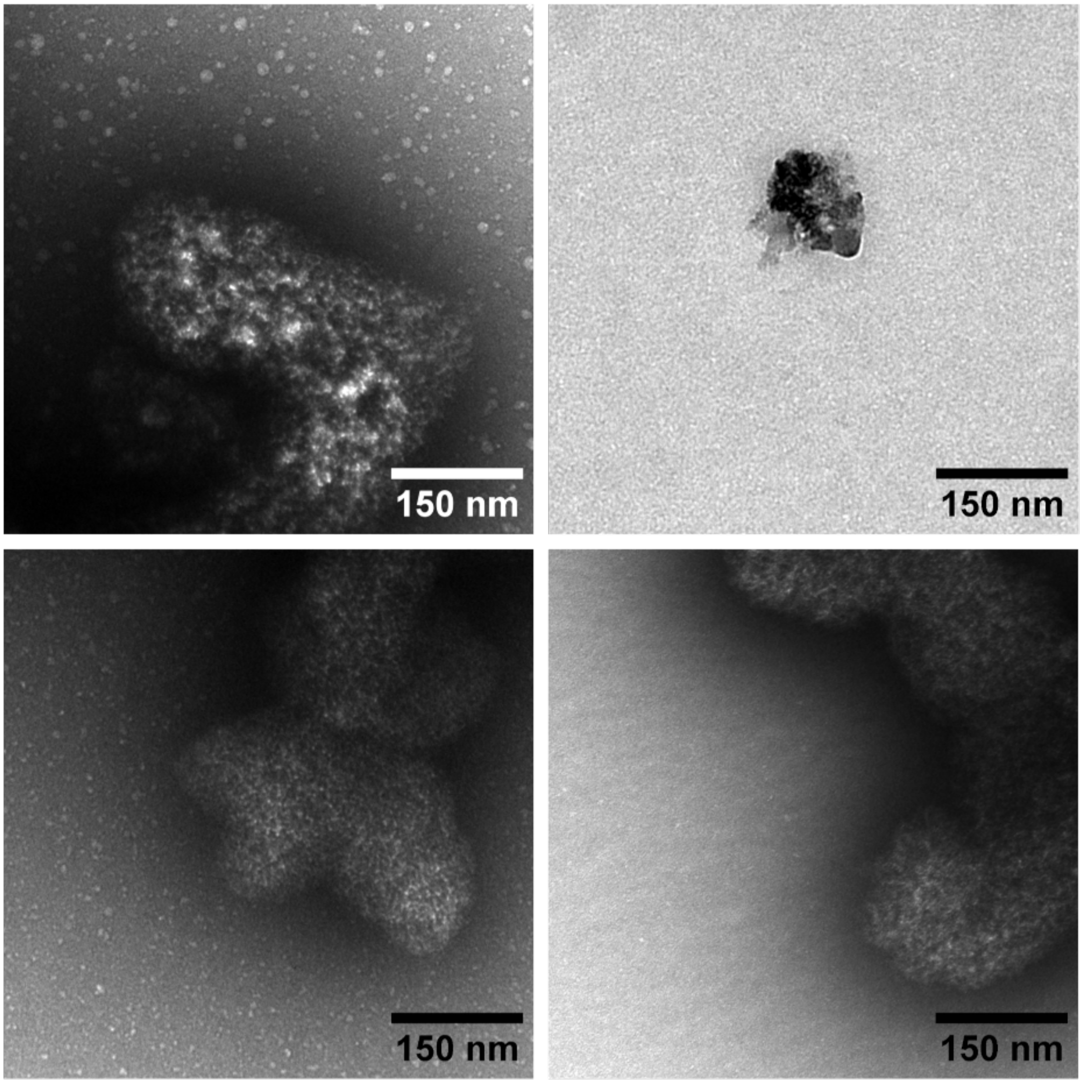

Figure S34. TEM images of 6HB exposed to 50% (v/v) DMSO for 24 h at room temperature. The TEM samples are negatively stained with uranyl formate (2% (w/v)). DMSO was removed by PEG precipitation before the TEM sample preparation.

4.3. 6HB in folding buffer exposed to ethanol

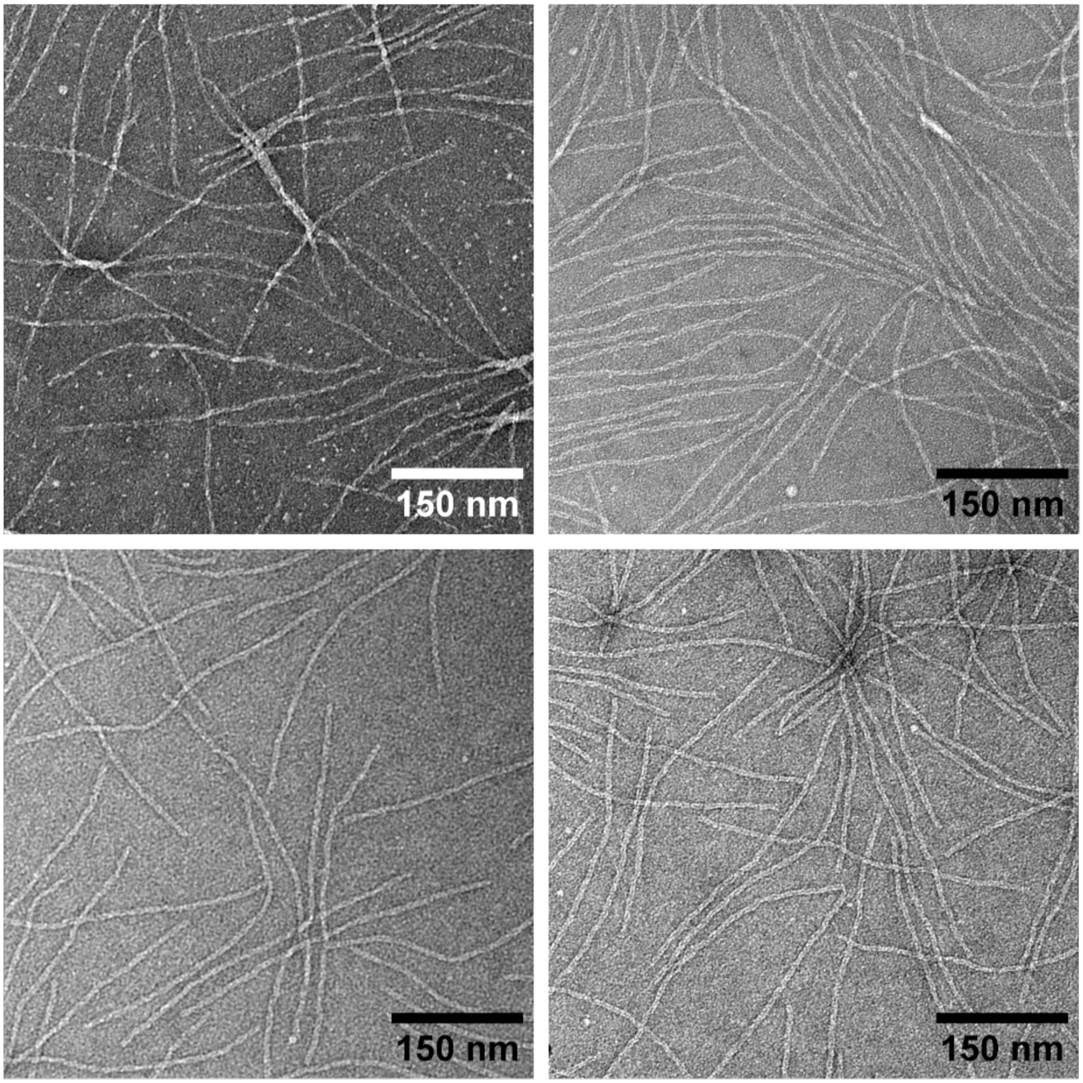

Figure S35. TEM images of 6HB in FOB exposed to 10% (v/v) ethanol for 24 h at room temperature. The TEM samples are negatively stained with uranyl formate (2% (w/v)). Ethanol was removed by PEG precipitation before the TEM sample preparation.

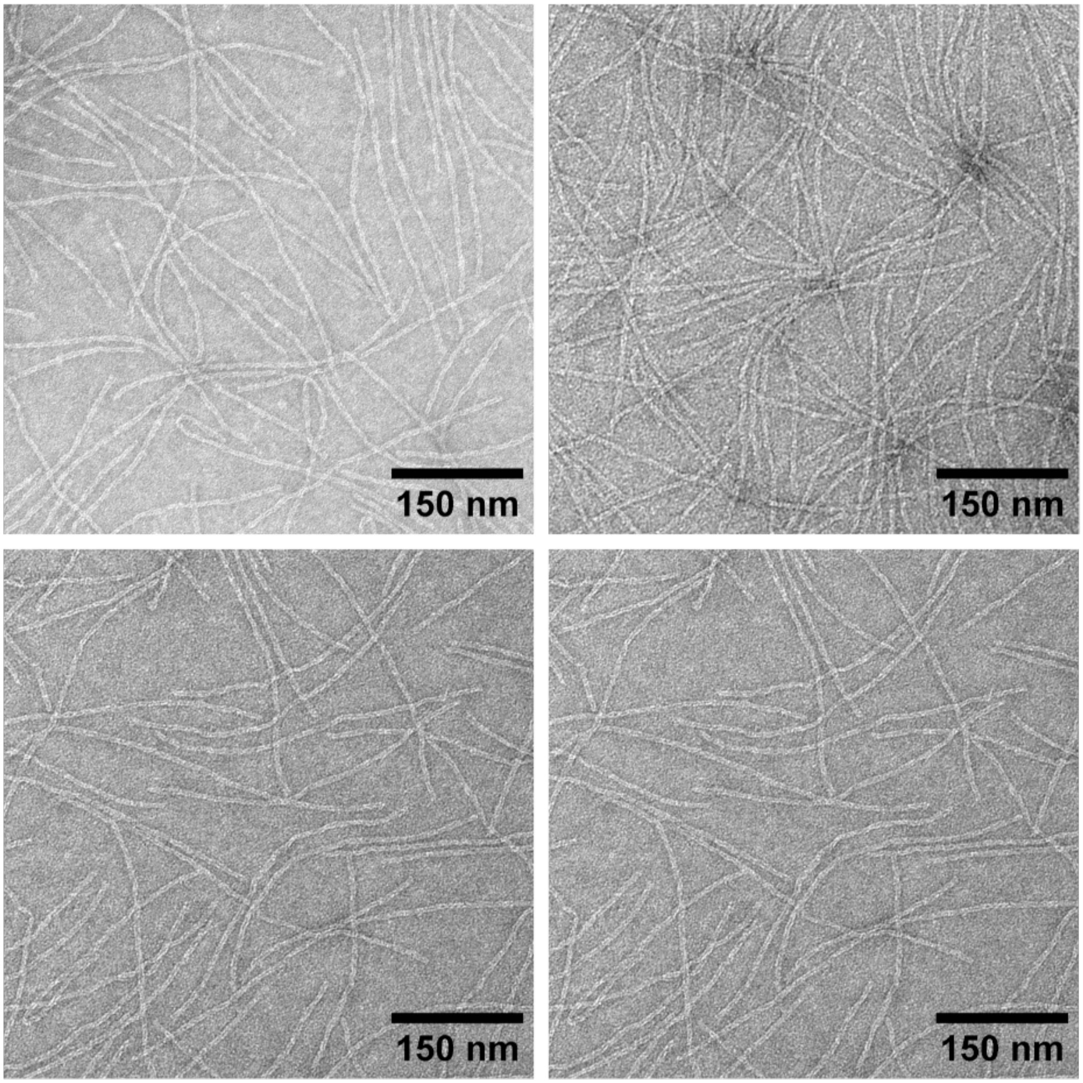

Figure S36. TEM images of 6HB in FOB exposed to 40% (v/v) ethanol for 24 h at room temperature. The TEM samples are negatively stained with uranyl formate (2% (w/v)). Ethanol was removed by PEG precipitation before the TEM sample preparation.

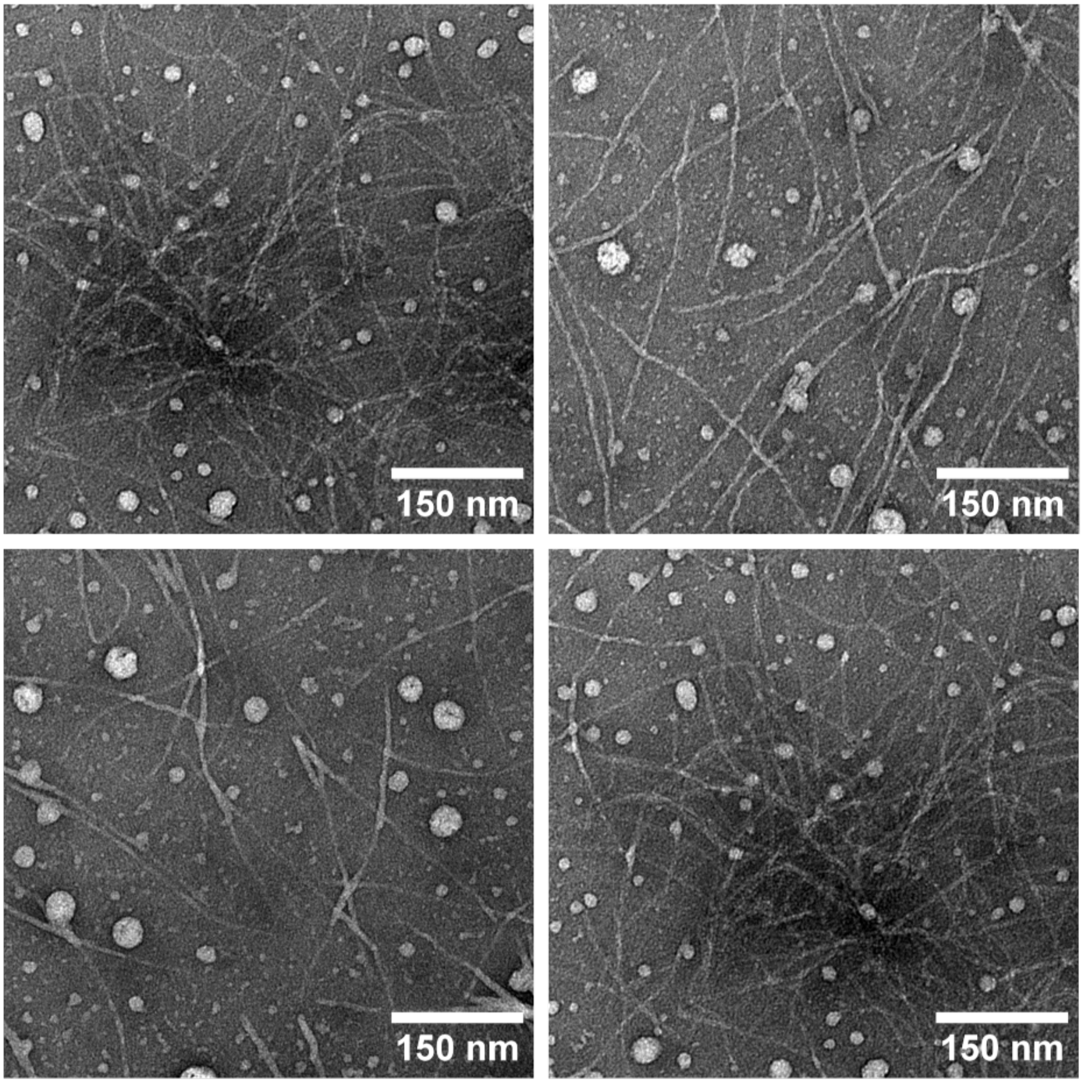

Figure S37. TEM images of 6HB in FOB exposed to 90% (v/v) ethanol for 24 h at room temperature. The TEM samples are negatively stained with uranyl formate (2% (w/v)). Ethanol was removed by PEG precipitation before the TEM sample preparation.

4.4. 6HB in folding buffer exposed to acetone

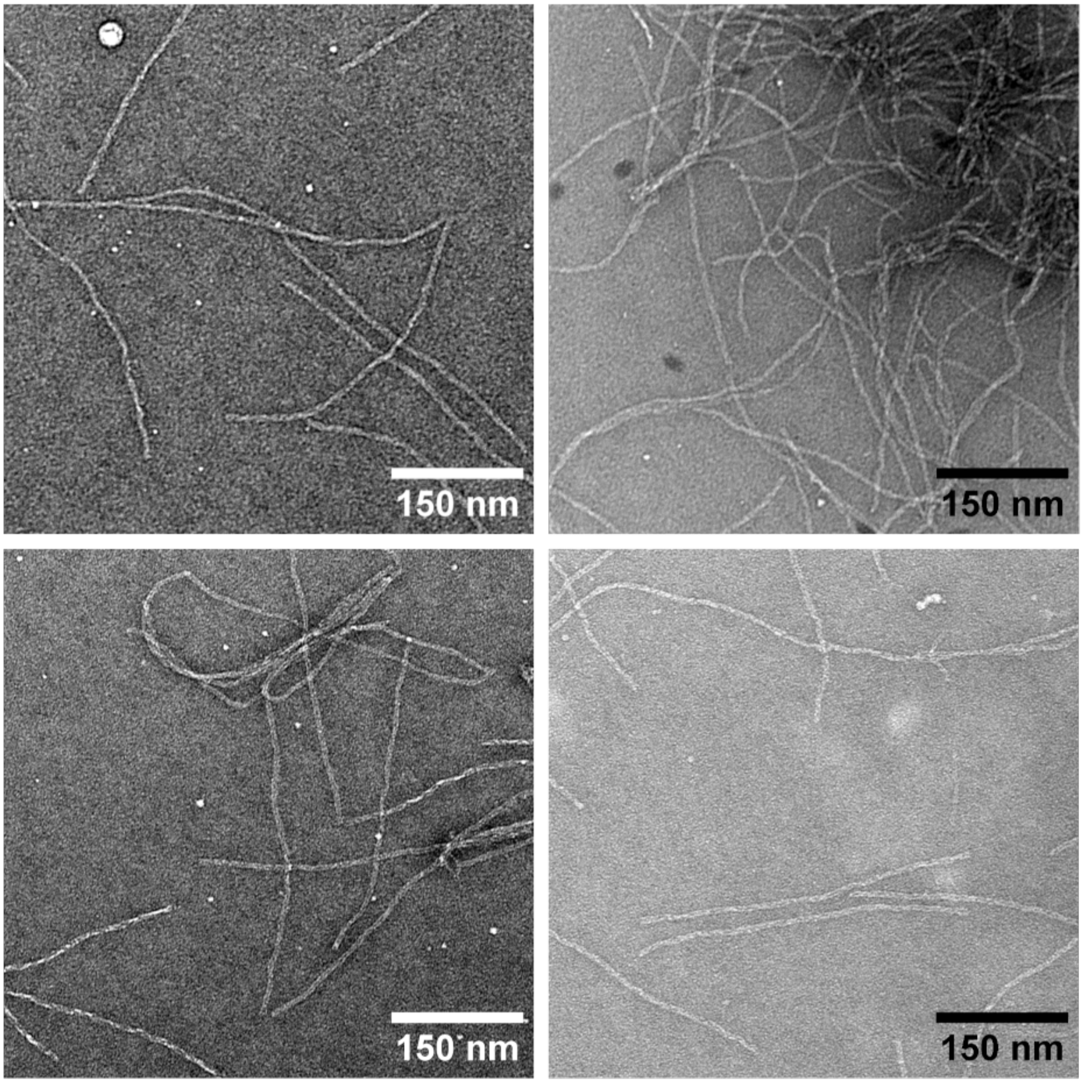

Figure S38. TEM images of 6HB in FOB exposed to 10% (v/v) acetone for 24 h at room temperature. The TEM samples are negatively stained with uranyl formate (2% (w/v)). Acetone was removed by PEG precipitation before the TEM sample preparation.

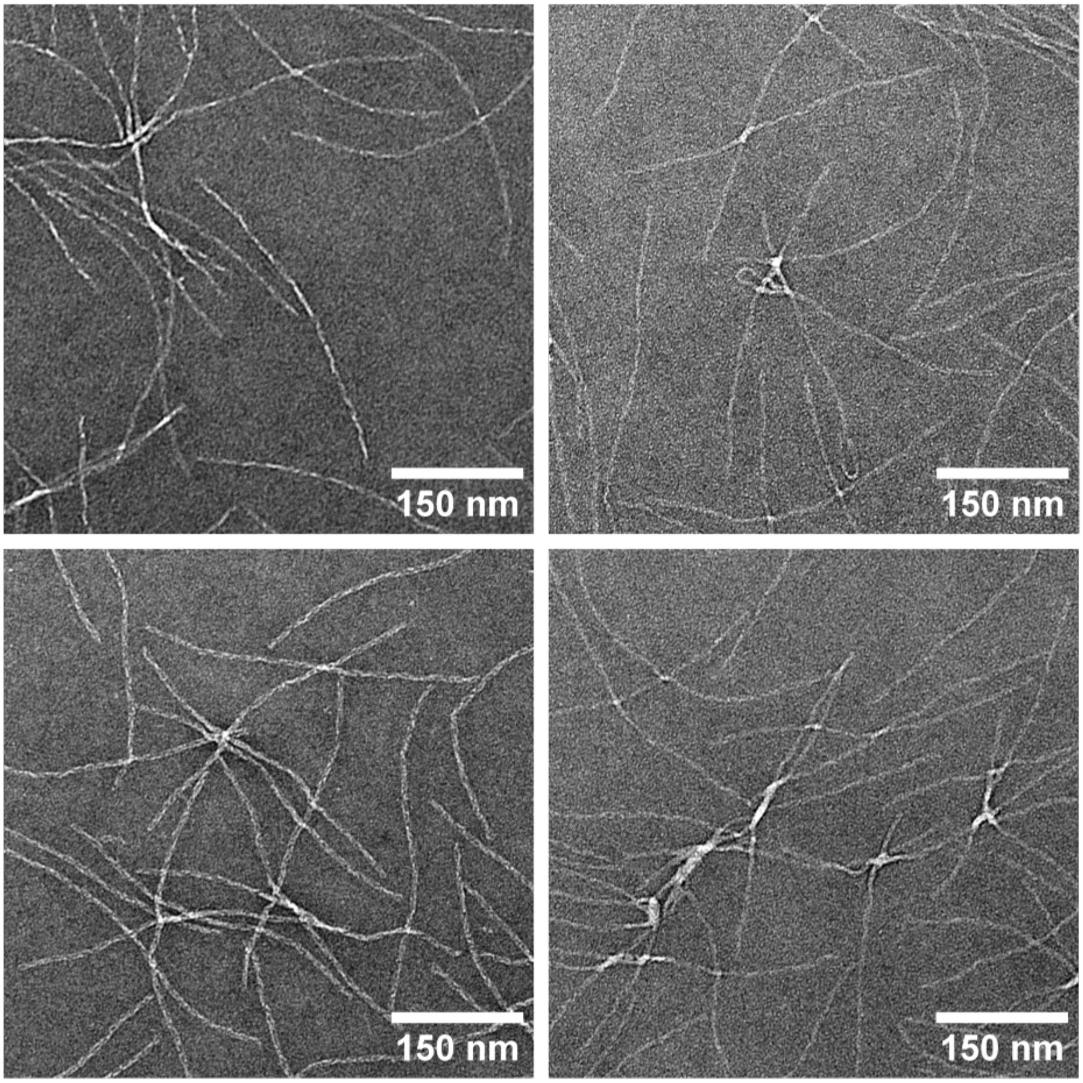

Figure S39. TEM images of 6HB in FOB exposed to 40% (v/v) acetone for 24 h at room temperature. The TEM samples are negatively stained with uranyl formate (2% (w/v)). Acetone was removed by PEG precipitation before the TEM sample preparation.

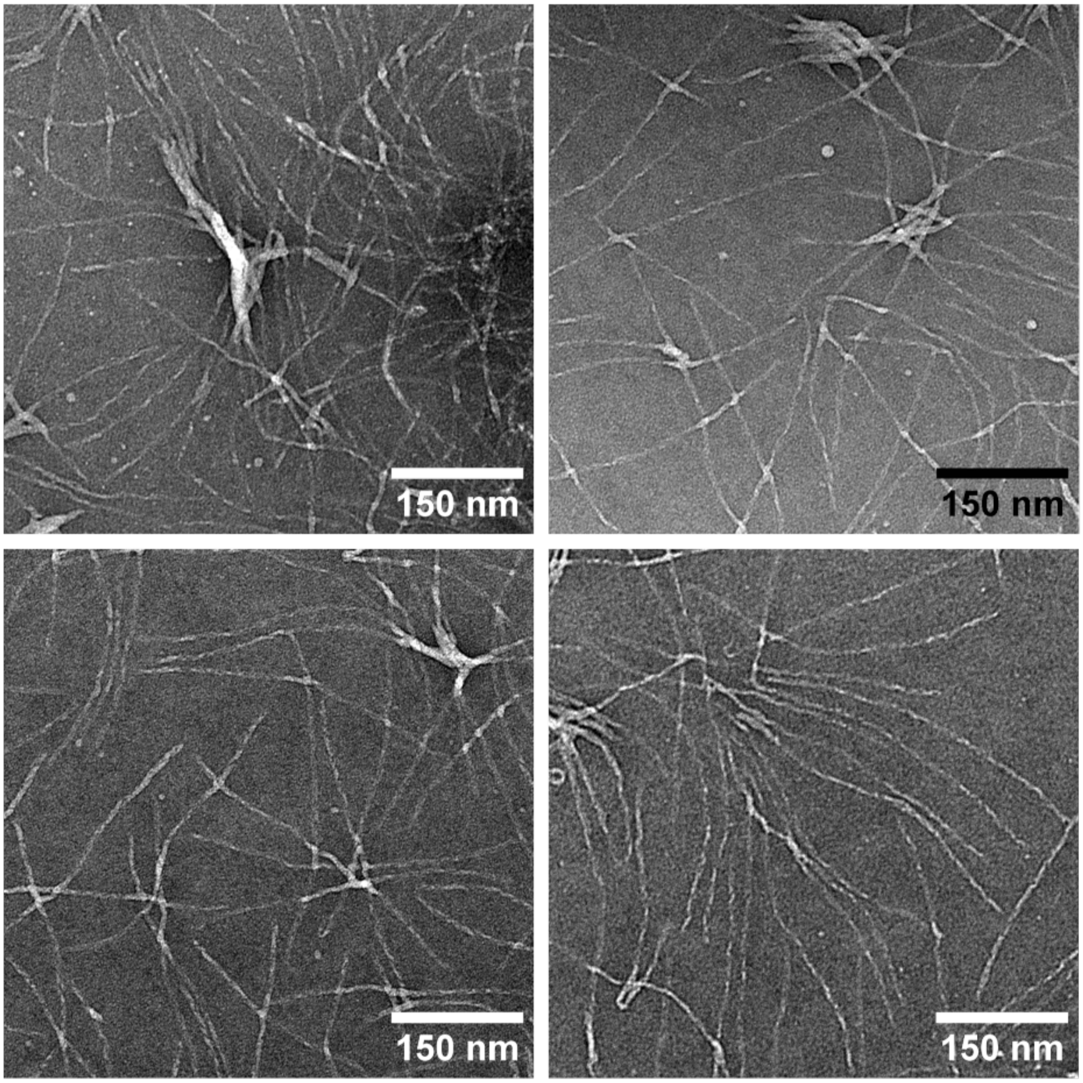

Figure S40. TEM images of 6HB in FOB exposed to 90% (v/v) acetone for 24 h at room temperature. The TEM samples are negatively stained with uranyl formate (2% (w/v)). Acetone was removed by PEG precipitation before the TEM sample preparation.

4.5. 24HB in folding buffer exposed to DMF

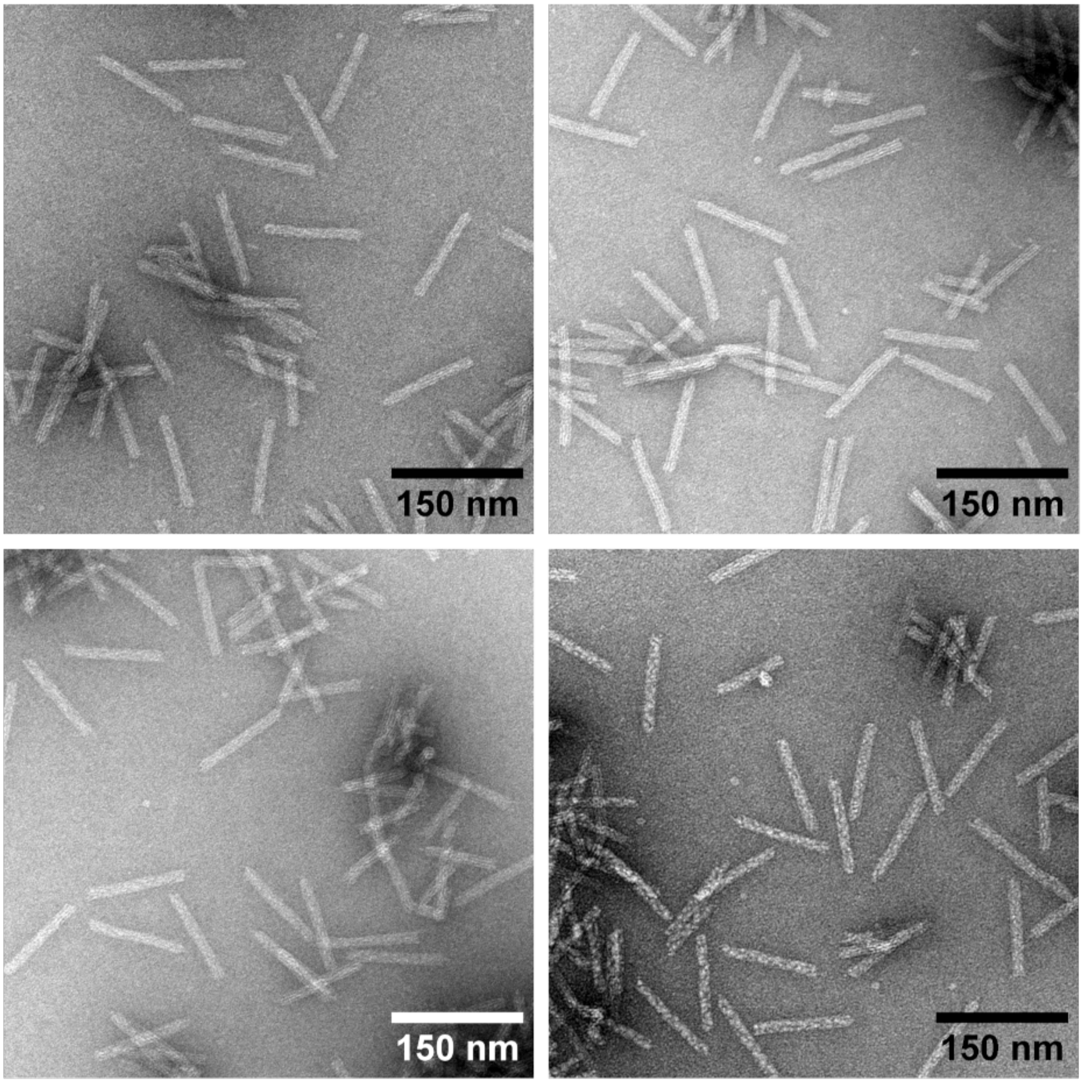

Figure S41. TEM images of 24HB in FOB exposed to 10% (v/v) DMF for 24 h at room temperature. The TEM samples are negatively stained with uranyl formate (2% (w/v)). DMF was removed by PEG precipitation before the TEM sample preparation.

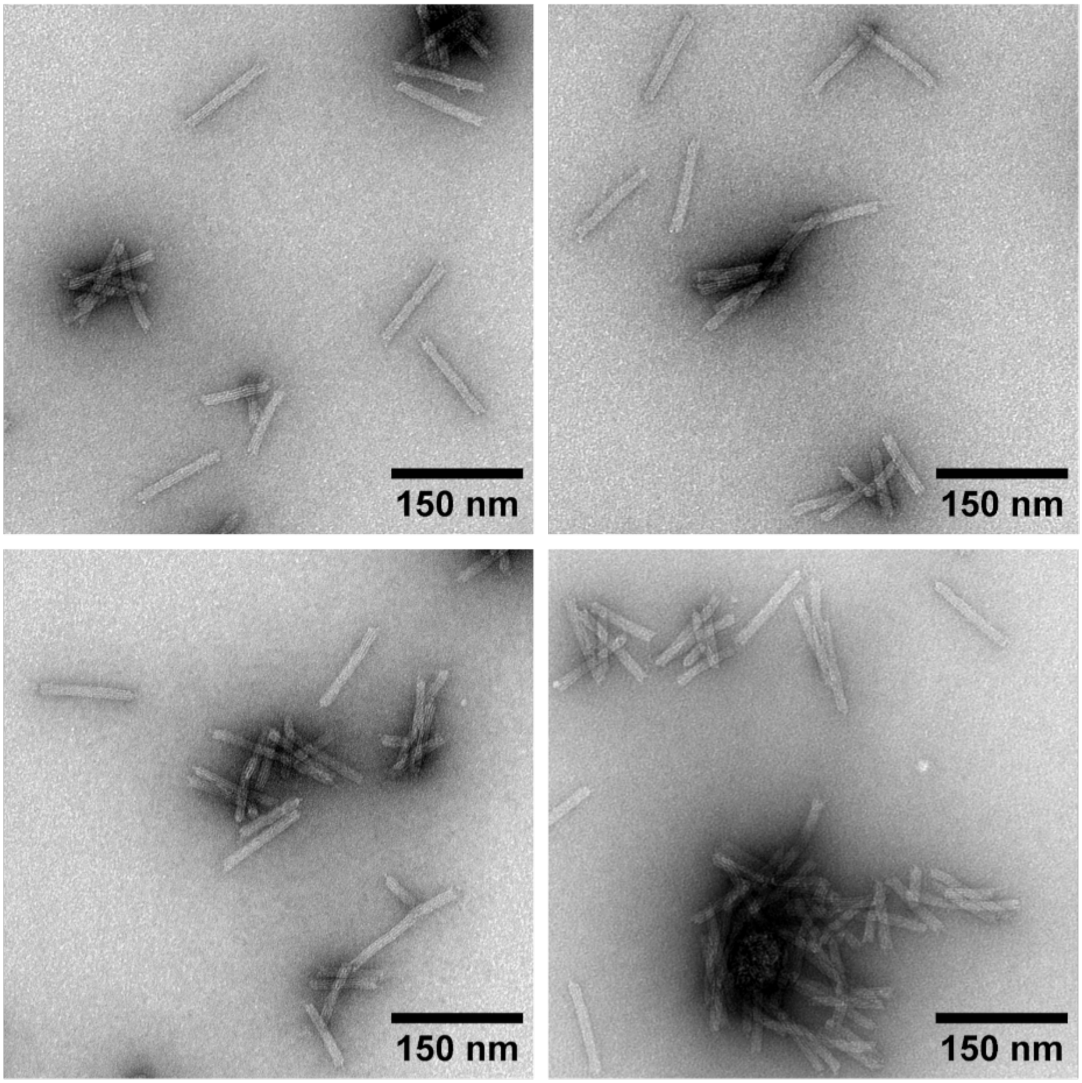

Figure S42. TEM images of 24HB in FOB exposed to 30% (v/v) DMF for 24 h at room temperature. The TEM samples are negatively stained with uranyl formate (2% (w/v)). DMF was removed by PEG precipitation before the TEM sample preparation.

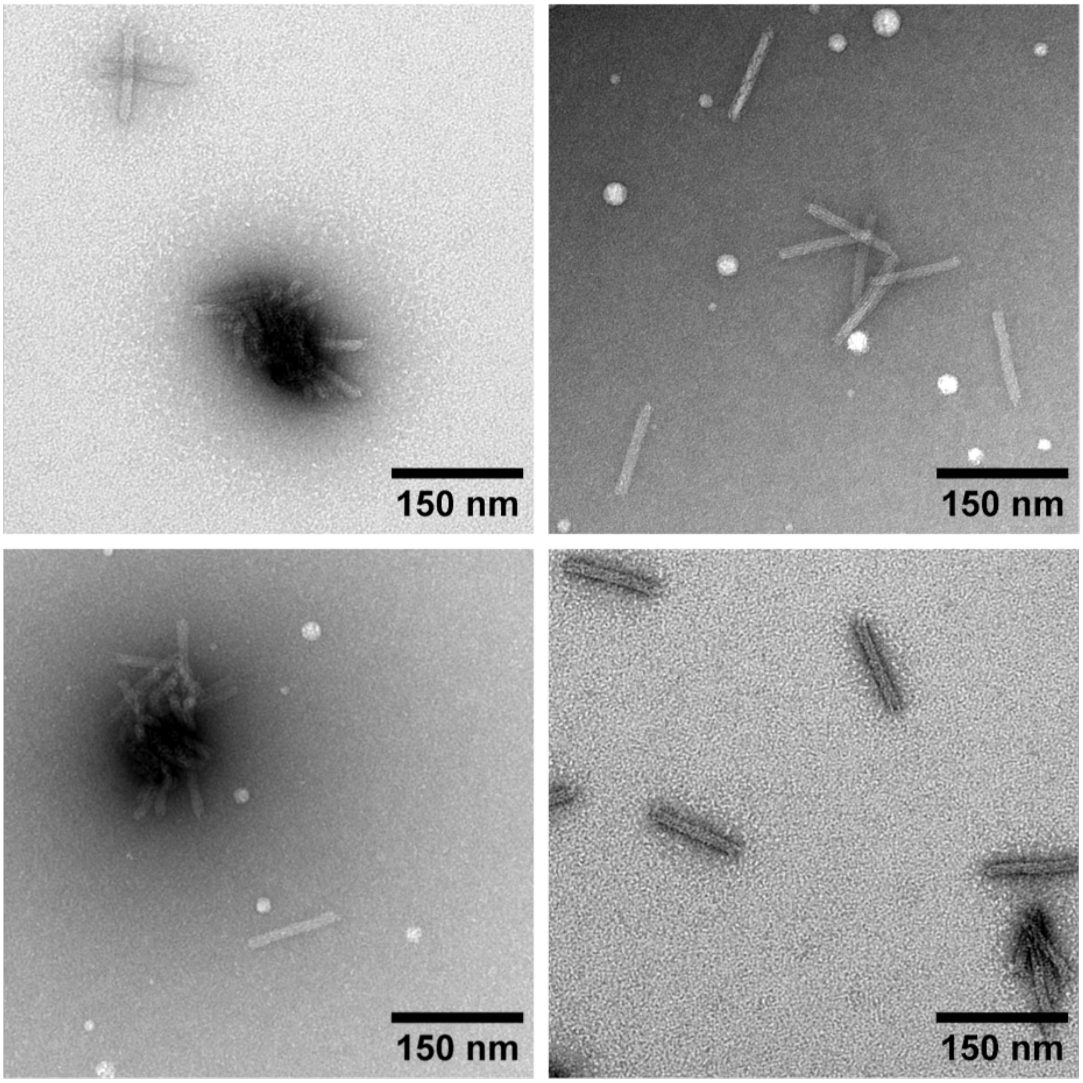

Figure S43. TEM images of 24HB in FOB exposed to 35% (v/v) DMF for 24 h at room temperature. The TEM samples are negatively stained with uranyl formate (2% (w/v)). DMF was removed by PEG precipitation before the TEM sample preparation.

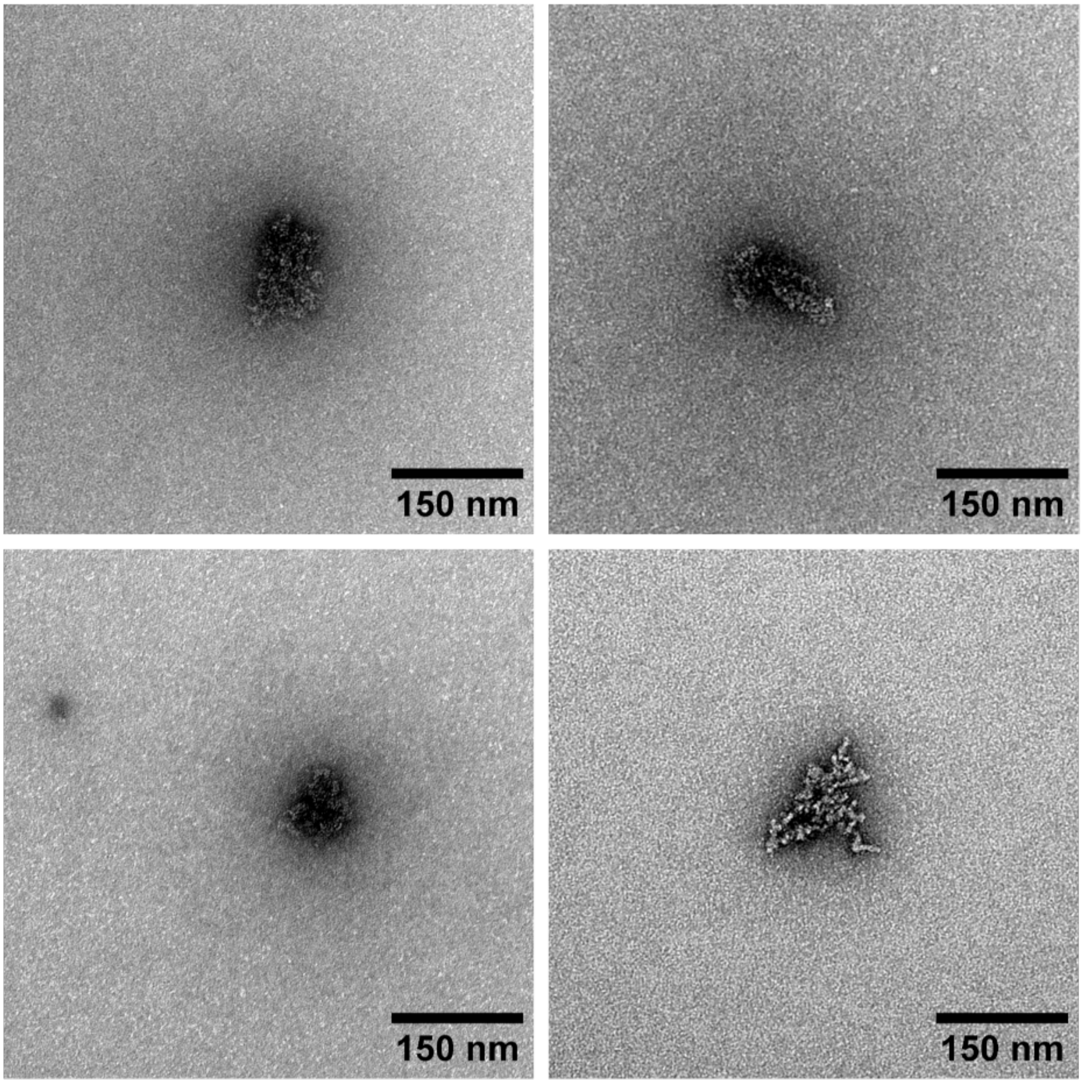

Figure S44. TEM images of 24HB in FOB exposed to 40% (v/v) DMF for 24 h at room temperature. The TEM samples are negatively stained with uranyl formate (2% (w/v)). DMF was removed by PEG precipitation before the TEM sample preparation.

4.6. 24HB in folding buffer exposed to DMSO

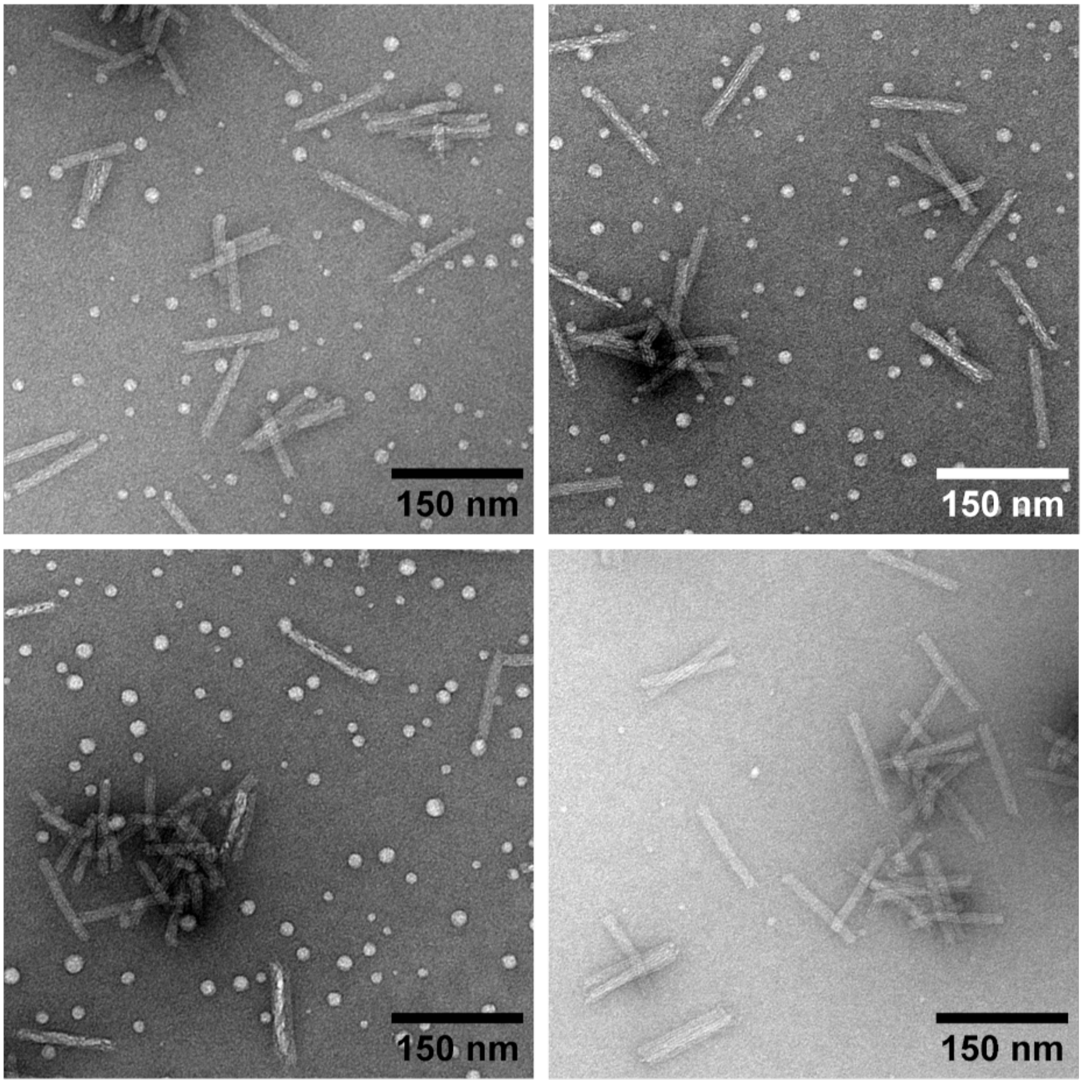

Figure S45. TEM images of 24HB in FOB exposed to 10% (v/v) DMSO for 24 h at room temperature. The TEM samples are negatively stained with uranyl formate (2% (w/v)). DMSO was removed by PEG precipitation before the TEM sample preparation.

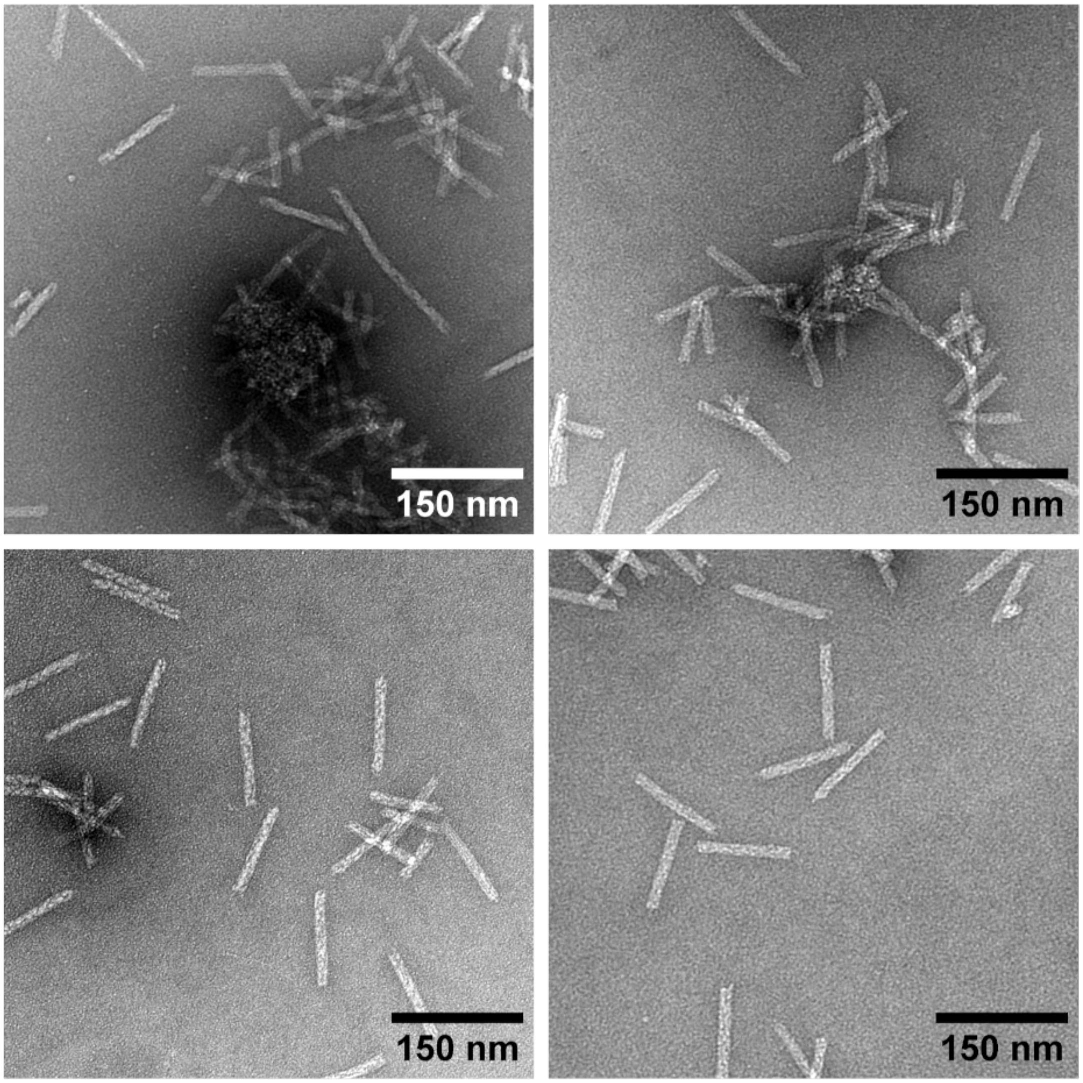

Figure S46. TEM images of 24HB in FOB exposed to 35% (v/v) DMSO for 24 h at room temperature. The TEM samples are negatively stained with uranyl formate (2% (w/v)). DMSO was removed by PEG precipitation before the TEM sample preparation.

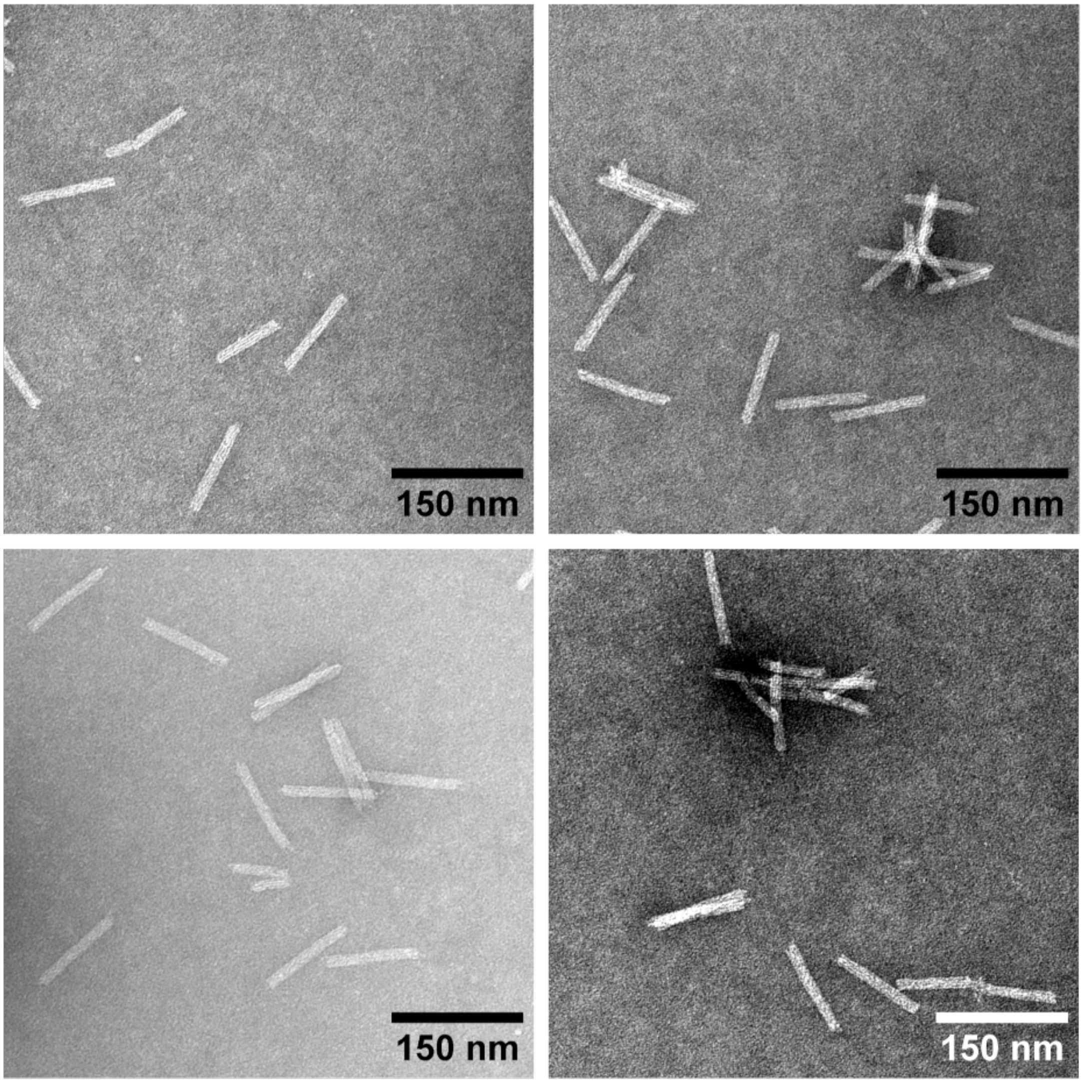

Figure S47. TEM images of 24HB in FOB exposed to 40% (v/v) DMSO for 24 h at room temperature. The TEM samples are negatively stained with uranyl formate (2% (w/v)). DMSO was removed by PEG precipitation before the TEM sample preparation.

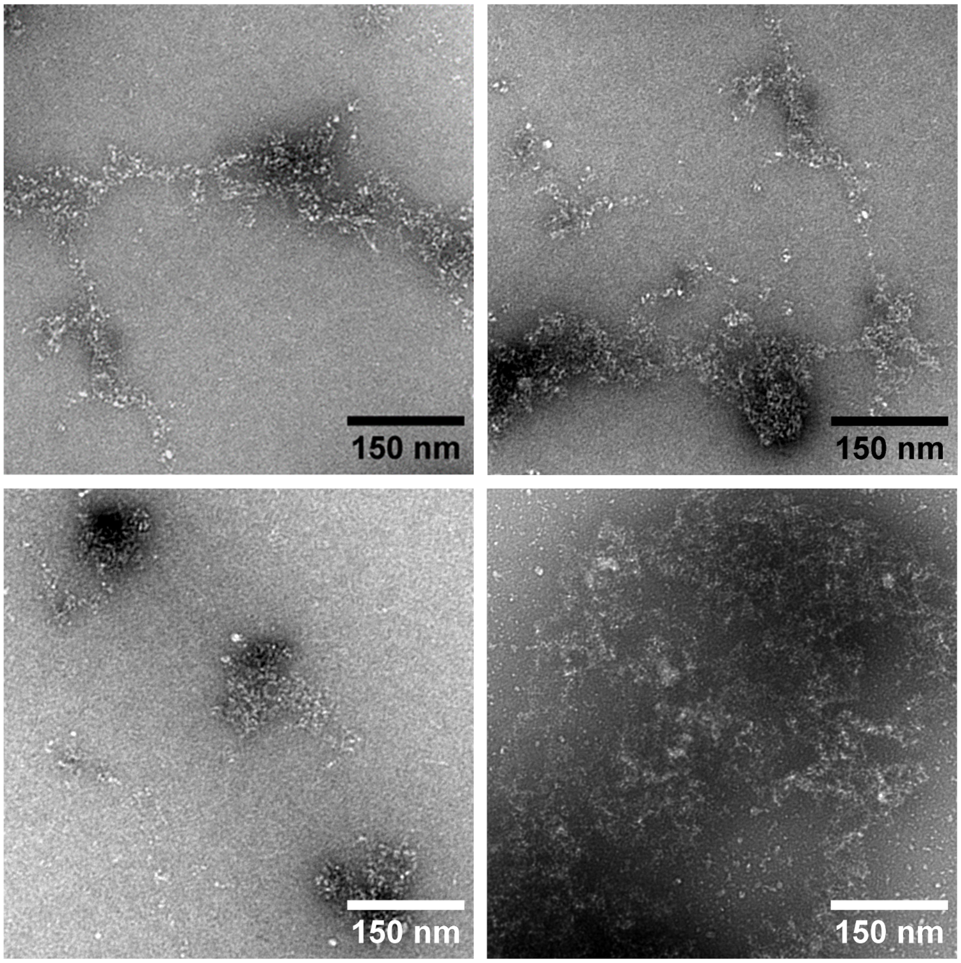

Figure S48. TEM images of 24HB in FOB exposed to 45% (v/v) DMSO for 24 h at room temperature. The TEM samples are negatively stained with uranyl formate (2% (w/v)). DMSO was removed by PEG precipitation before the TEM sample preparation.

4.7. 24HB in folding buffer exposed to ethanol

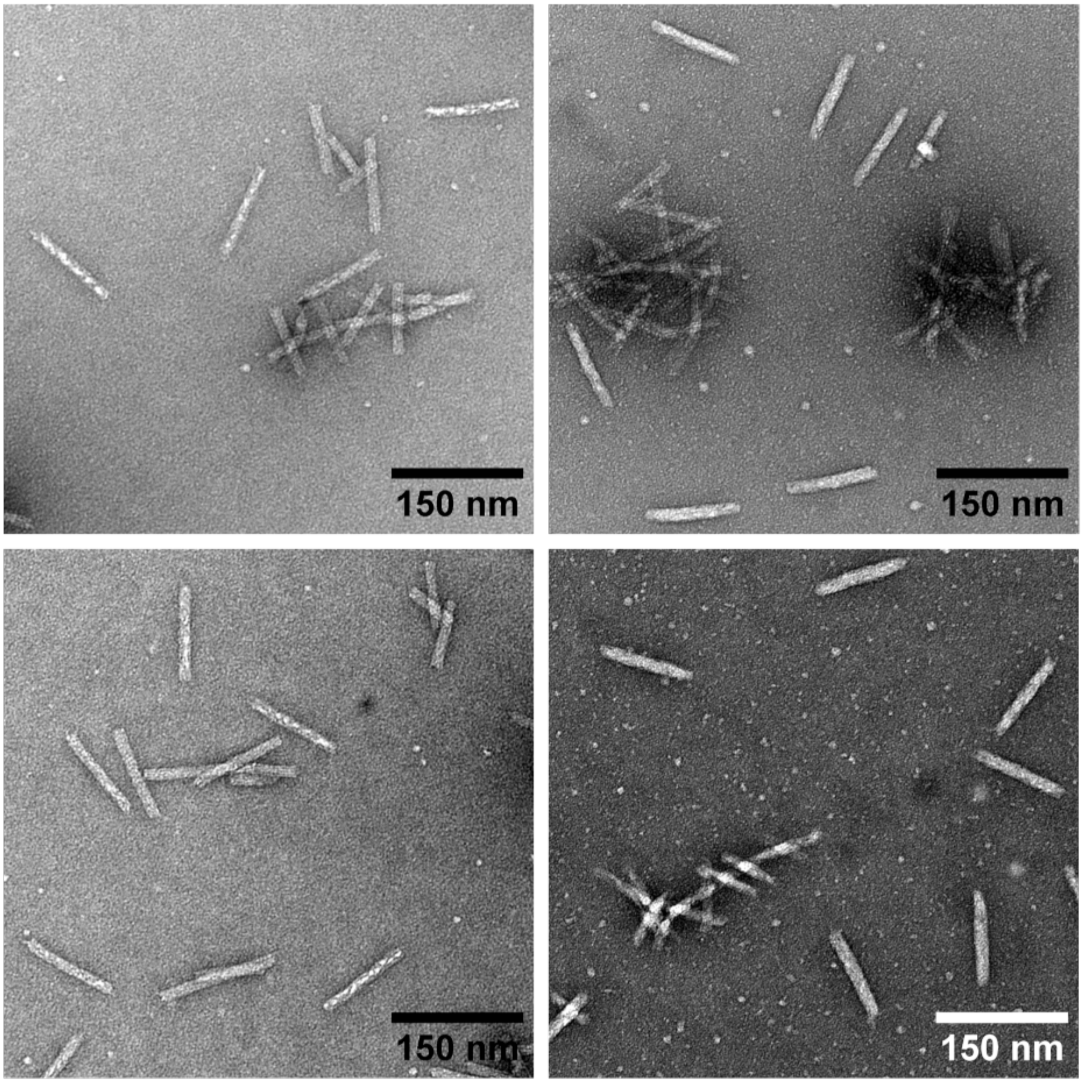

Figure S49. TEM images of 24HB in FOB exposed to 10% (v/v) ethanol for 24 h at room temperature. The TEM samples are negatively stained with uranyl formate (2% (w/v)). Ethanol was removed by PEG precipitation before the TEM sample preparation.

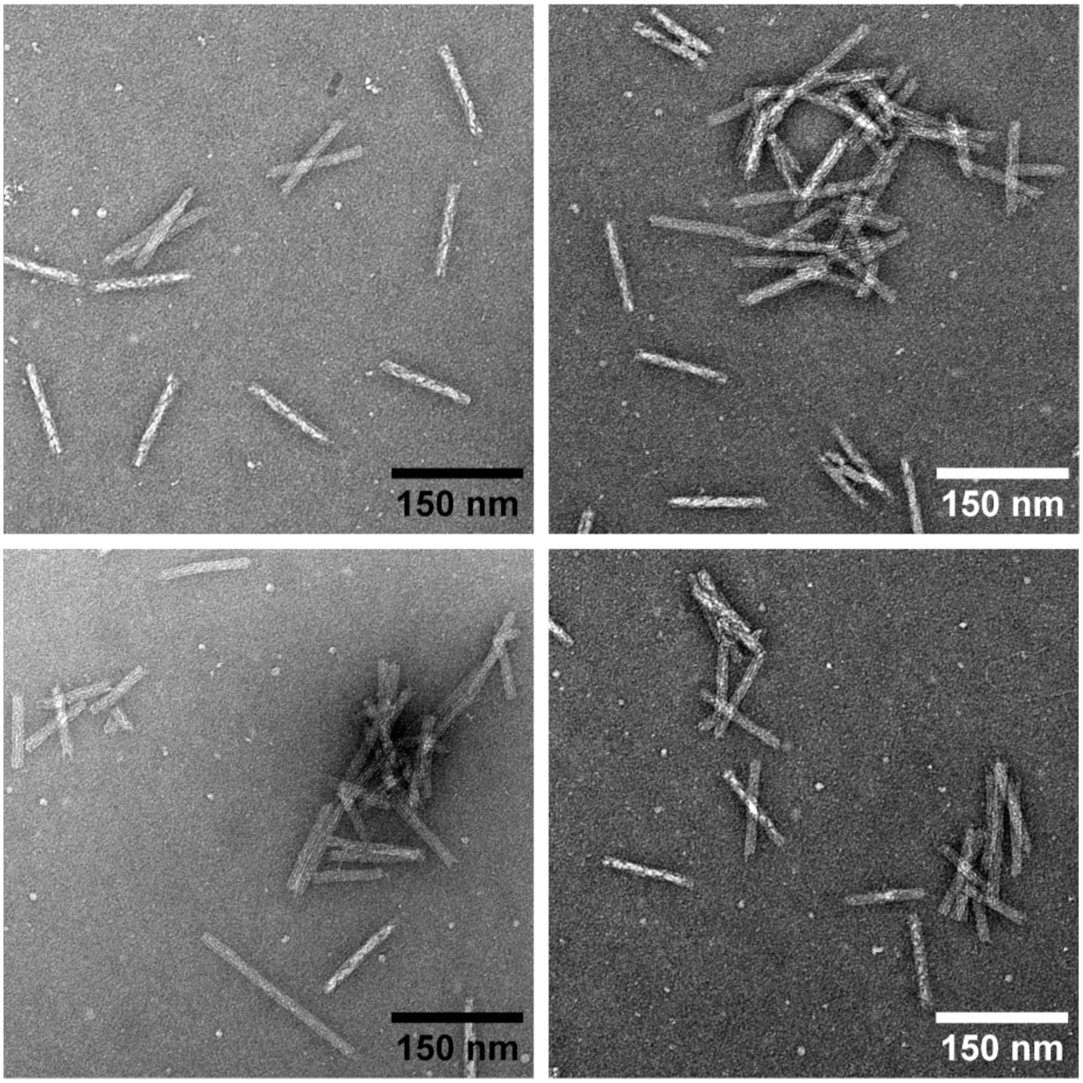

Figure S50. TEM images of 24HB in FOB exposed to 40% (v/v) ethanol for 24 h at room temperature. The TEM samples are negatively stained with uranyl formate (2% (w/v)). Ethanol was removed by PEG precipitation before the TEM sample preparation.

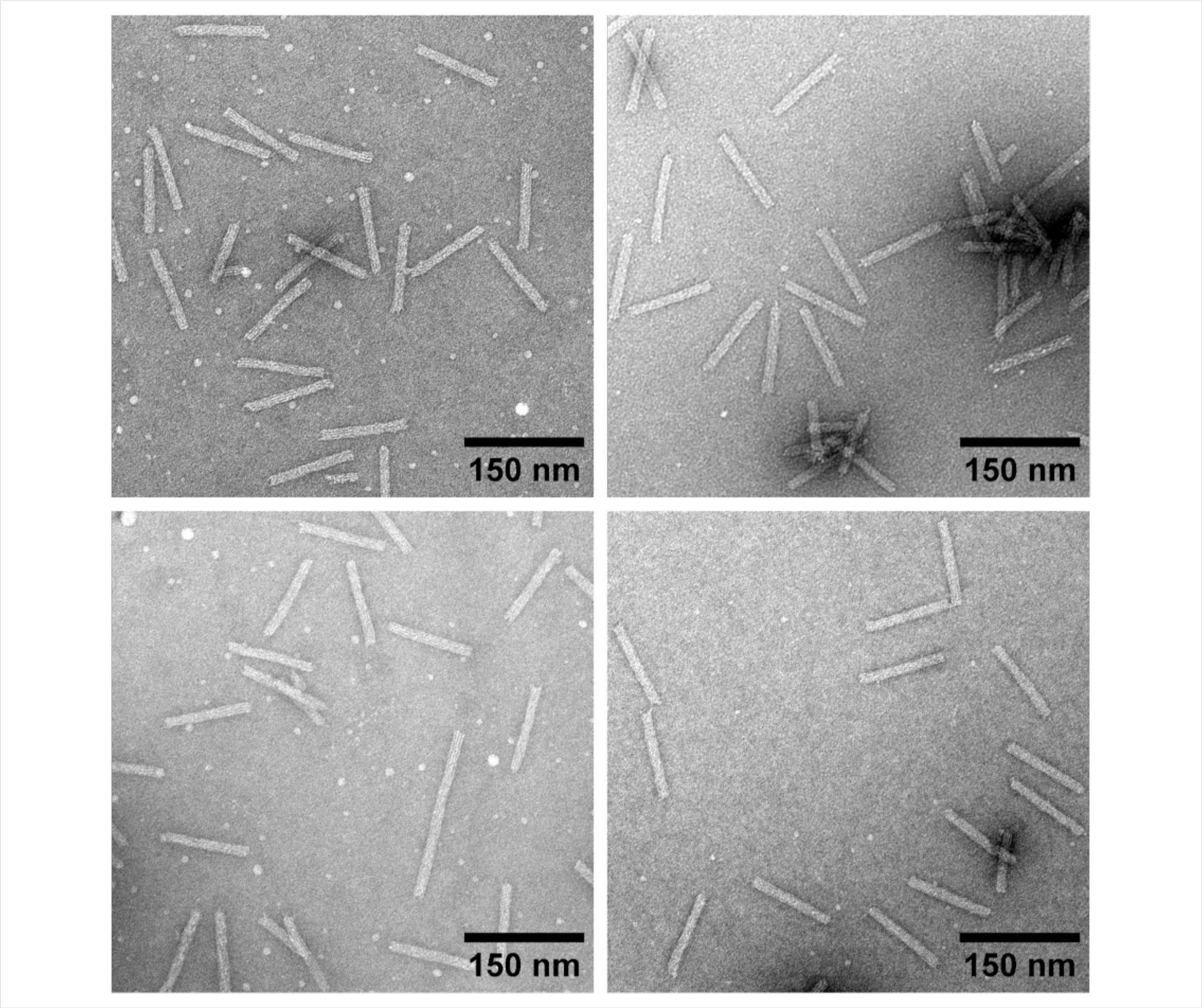

Figure S51. TEM images of 24HB in FOB exposed to 90% (v/v) ethanol for 24 h at room temperature. The TEM samples are negatively stained with uranyl formate (2% (w/v)). Ethanol was removed by PEG precipitation before the TEM sample preparation.

4.8. 24HB in folding buffer exposed to acetone

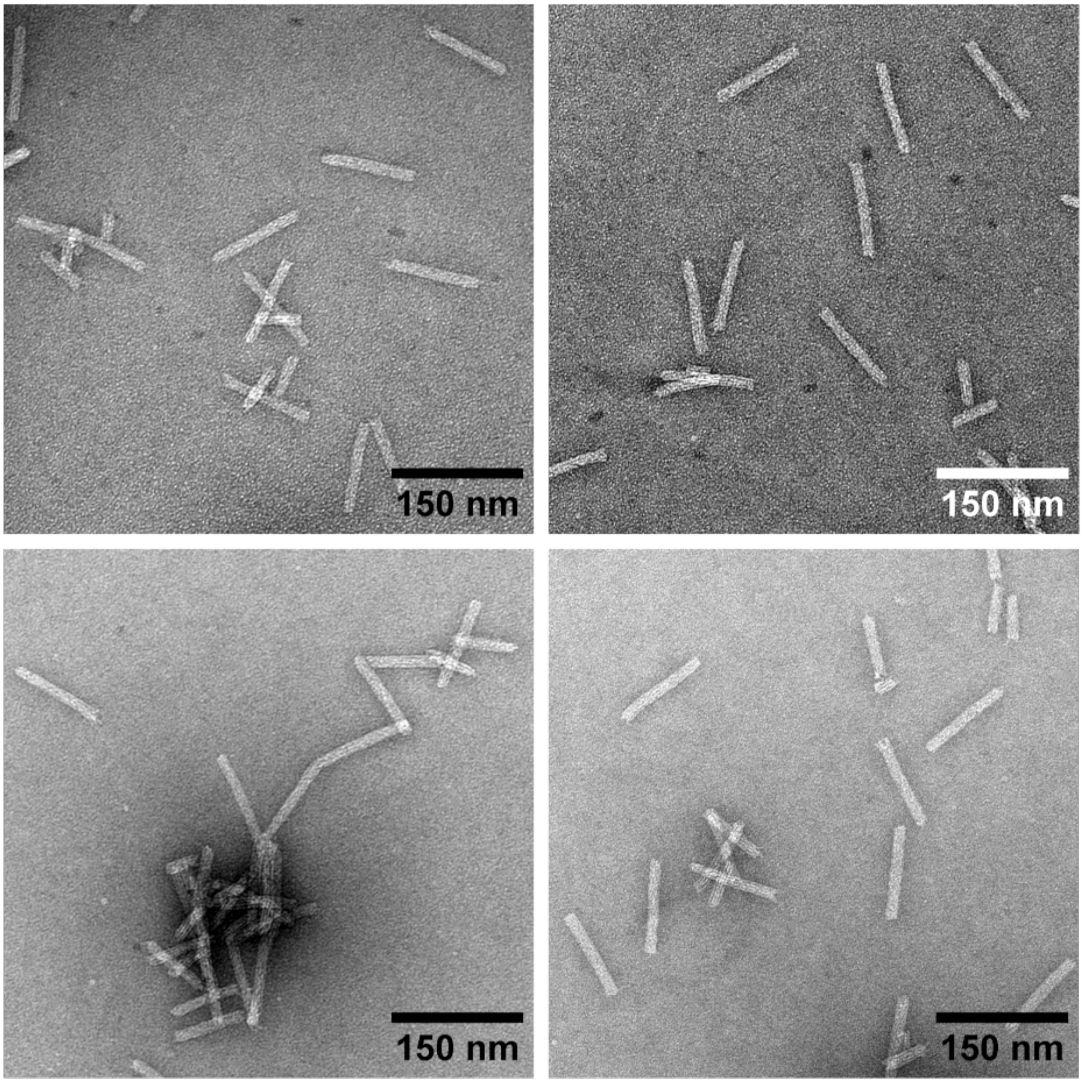

Figure S52. TEM images of 24HB in FOB exposed to 10% (v/v) acetone for 24 h at room temperature. The TEM samples are negatively stained with uranyl formate (2% (w/v)). Acetone was removed by PEG precipitation before the TEM sample preparation.

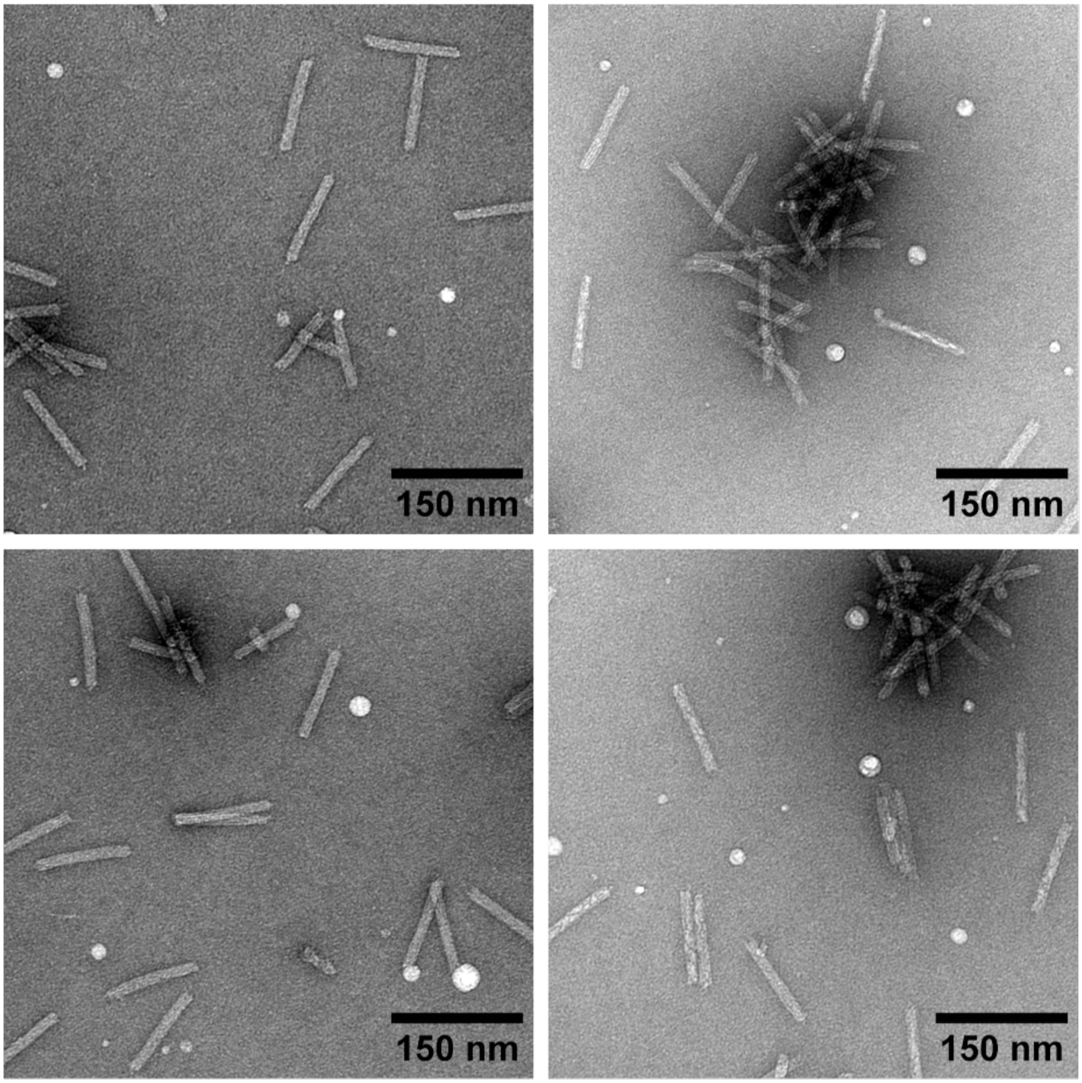

Figure S53. TEM images of 24HB in FOB exposed to 40% (v/v) acetone for 24 h at room temperature. The TEM samples are negatively stained with uranyl formate (2% (w/v)). Acetone was removed by PEG precipitation before the TEM sample preparation.

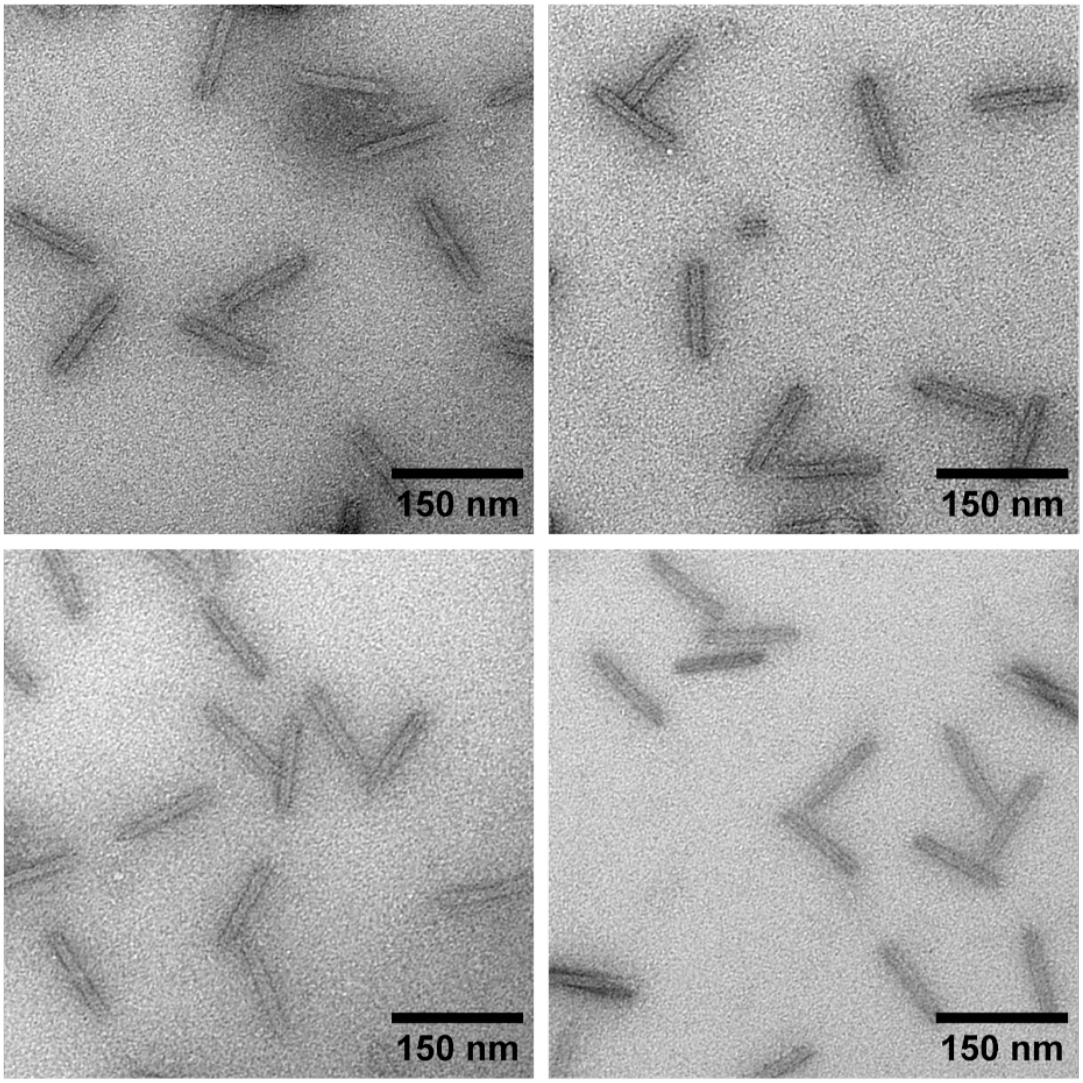

Figure S54. TEM images of 24HB in FOB exposed to 90% (v/v) acetone for 24 h at room temperature. The TEM samples are negatively stained with uranyl formate (2% (w/v)). Acetone was removed by PEG precipitation before the TEM sample preparation.

5. Additional agarose gels in deionized water

5.1. DNA origami triangle in deionized water

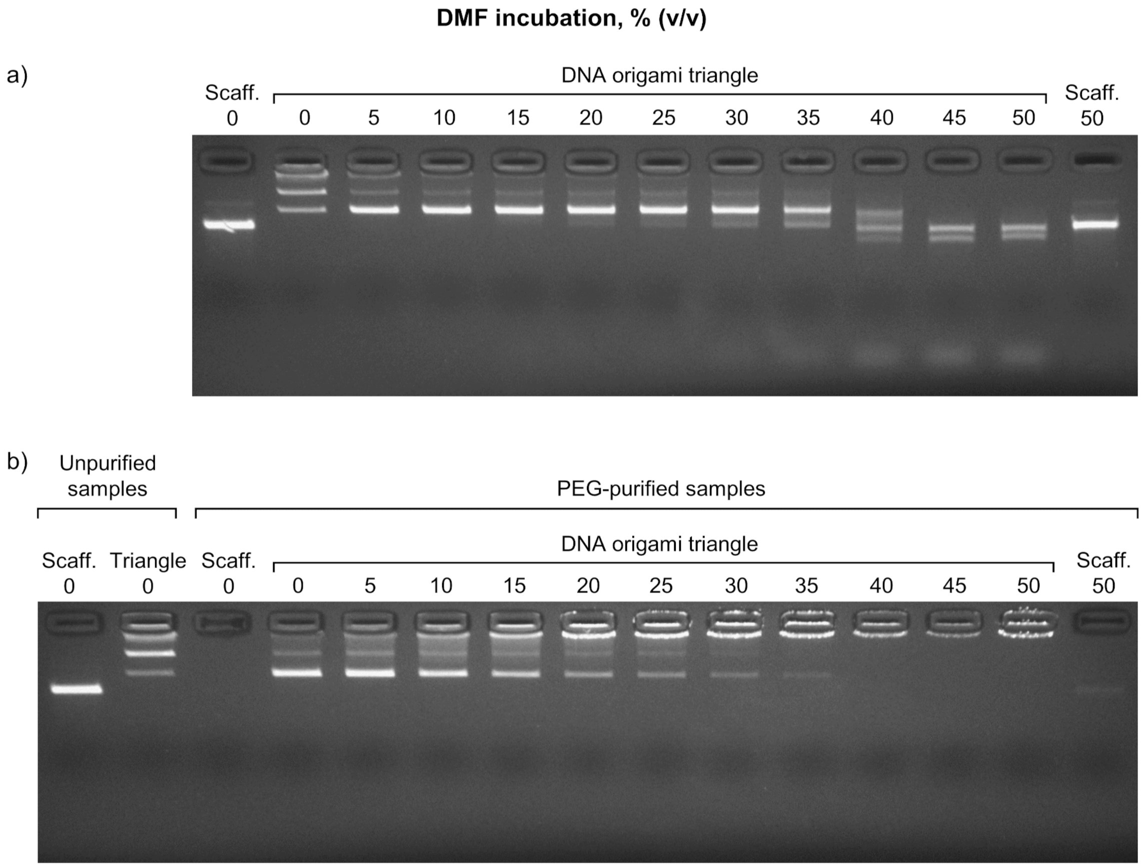

Figure S55. AGE of DNA origami triangles in deionized water exposed to DMF for 24 h at room temperature a) before and b) after removing DMF by PEG precipitation. The p7249 scaffold concentration is 10 nM and the DNA origami concentration is 5 nM in the gel.

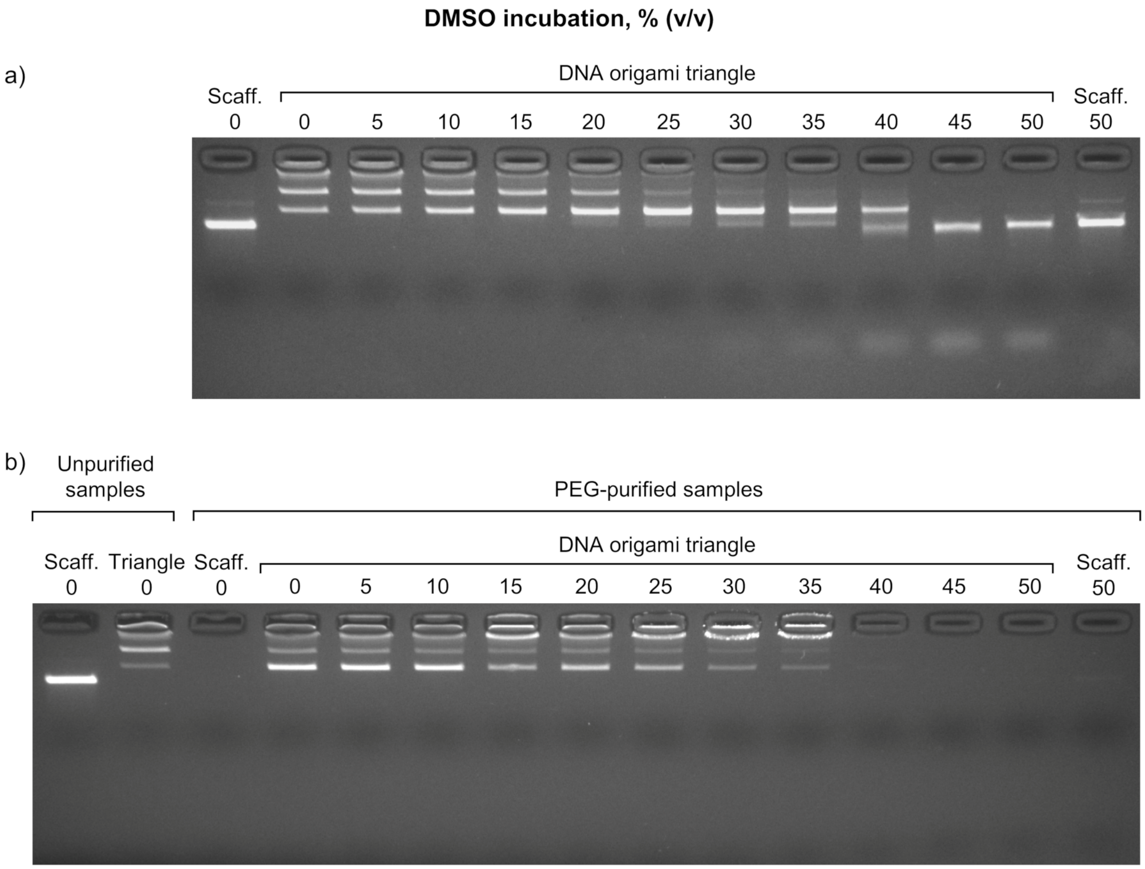

Figure S56. AGE of DNA origami triangles in deionized water exposed to DMSO for 24 h at room temperature a) before and b) after removing DMSO by PEG precipitation. The p7249 scaffold concentration is 10 nM and the DNA origami concentration is 5 nM in the gel.

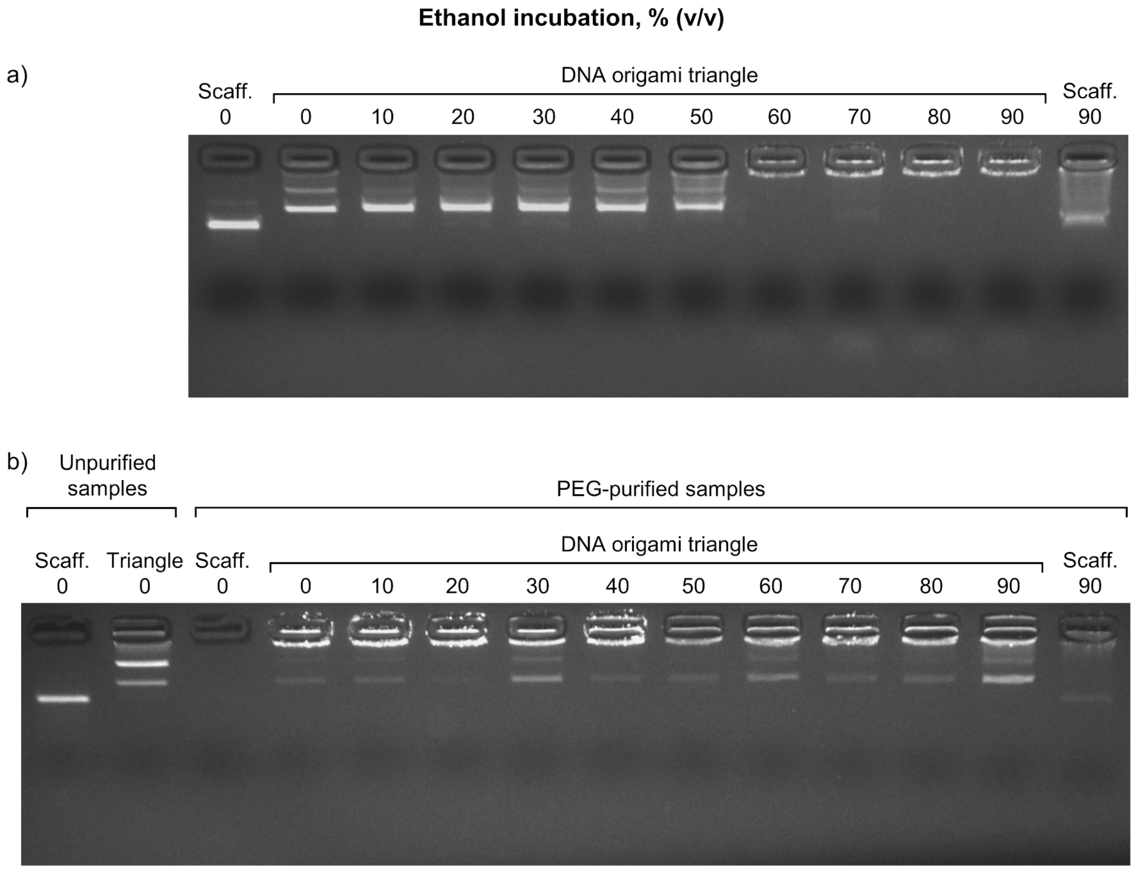

Figure S57. AGE of DNA origami triangles in deionized water exposed to ethanol for 24 h at room temperature a) before and b) after removing ethanol by PEG precipitation. The p7249 scaffold concentration is 10 nM and the DNA origami concentration is 5 nM in the gel.

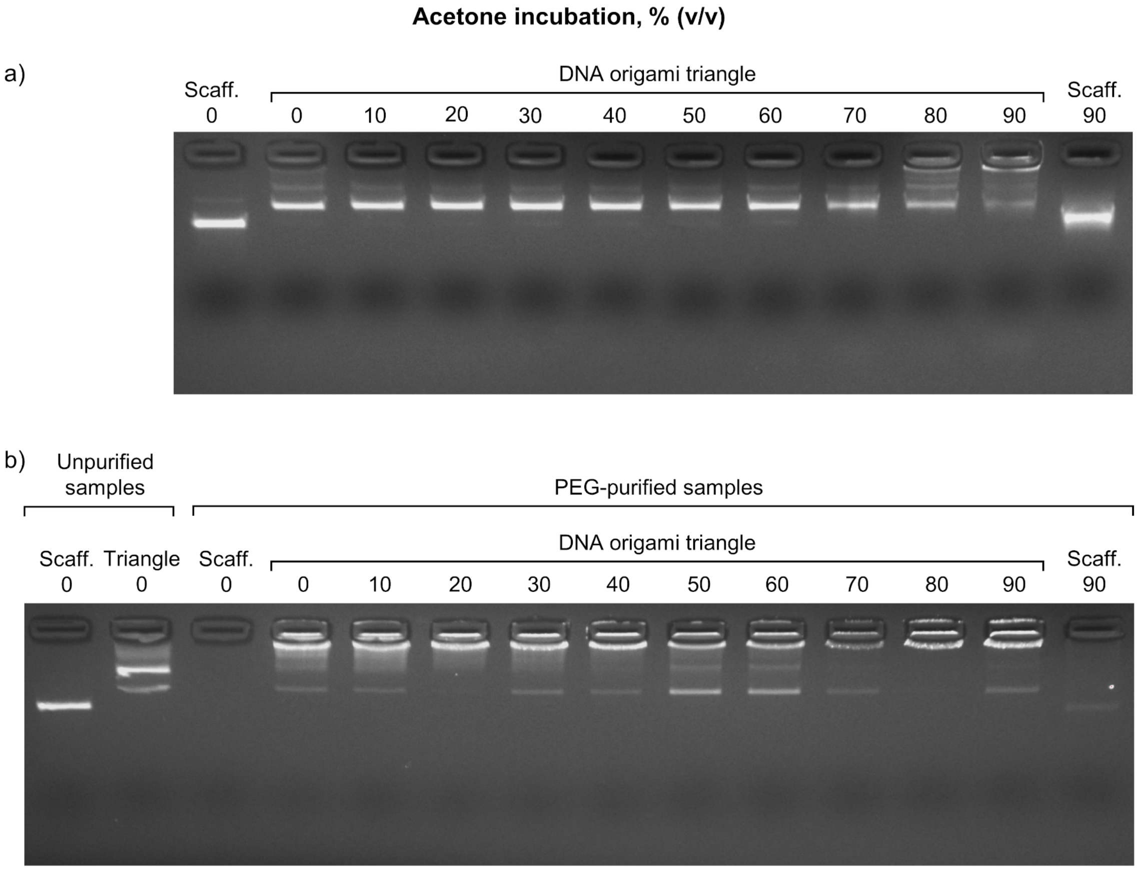

Figure S58. AGE of DNA origami triangles in deionized water exposed to acetone for 24 h at room temperature a) before and b) after removing acetone by PEG precipitation. The p7249 scaffold concentration is 10 nM and the DNA origami concentration is 5 nM in the gel.

5.2. 6HB in deionized water

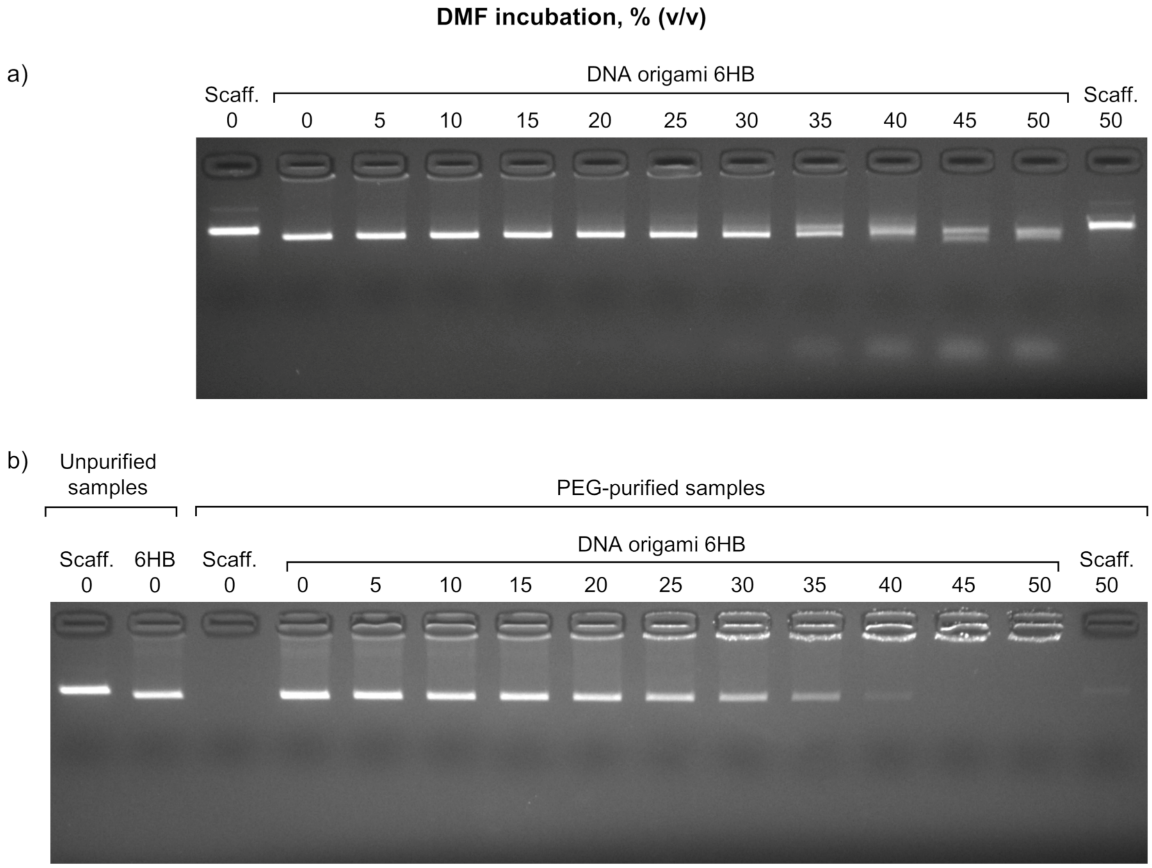

Figure S59. AGE of 6HB in deionized water exposed to DMF for 24 h at room temperature a) before and b) after removing DMF by PEG precipitation. The p7249 scaffold concentration is 10 nM and the DNA origami concentration is 5 nM in the gel.

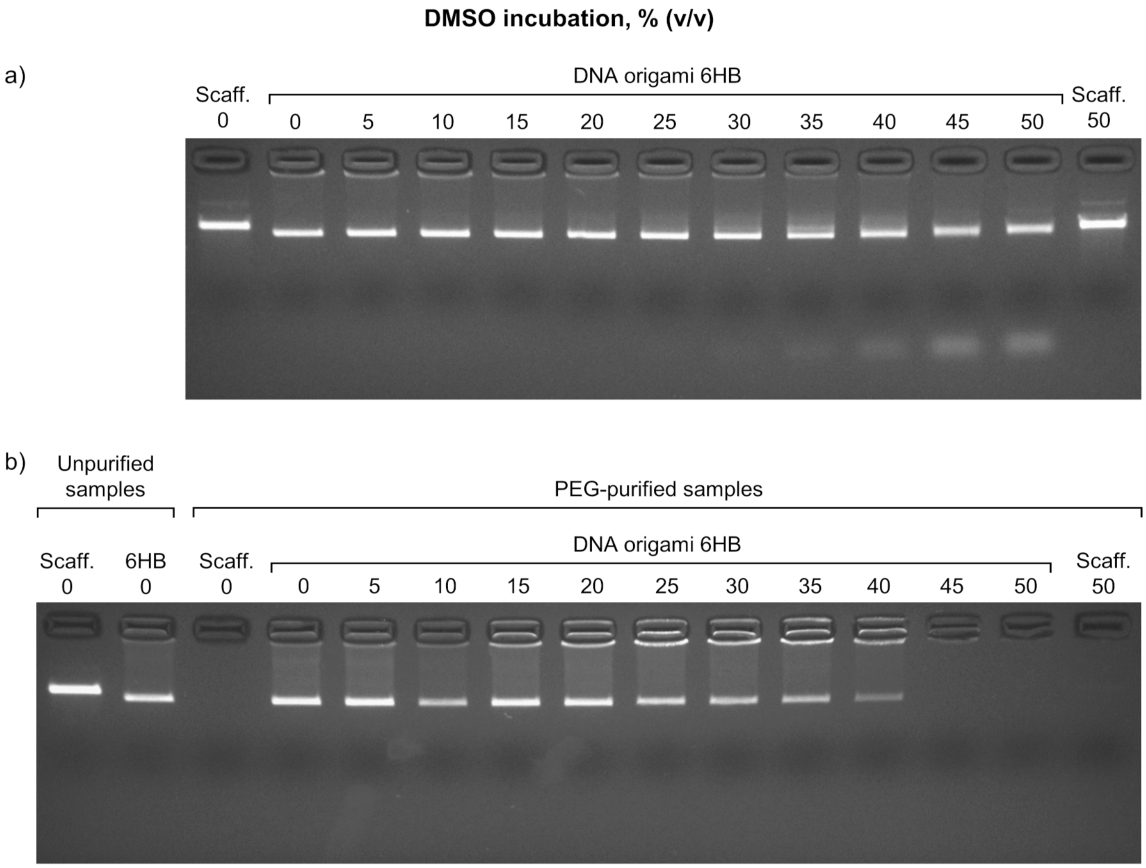

Figure S60. AGE of 6HB in deionized water exposed to DMSO for 24 h at room temperature a) before and b) after removing DMSO by PEG precipitation. The p7249 scaffold concentration is 10 nM and the DNA origami concentration is 5 nM in the gel.

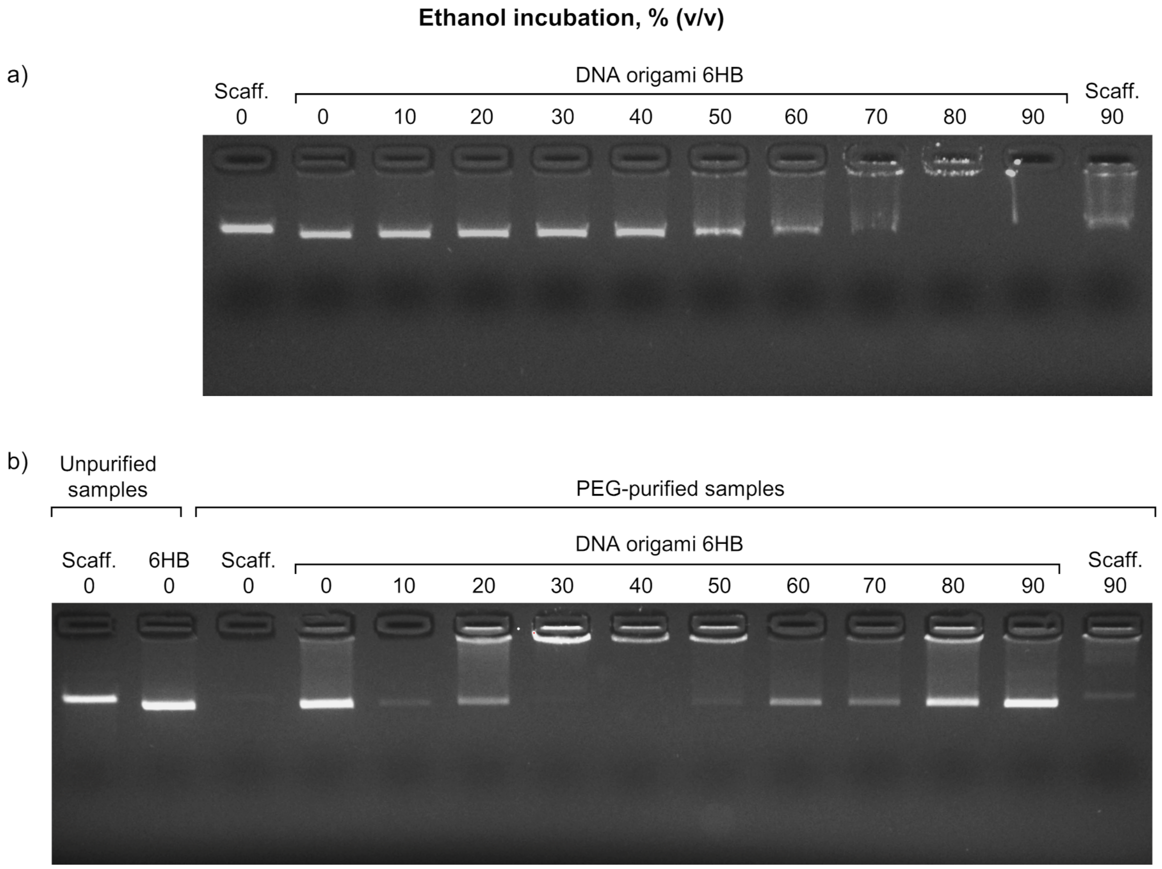

Figure S61. AGE of 6HB in deionized water exposed to ethanol for 24 h at room temperature a) before and b) after removing ethanol by PEG precipitation. The p7249 scaffold concentration is 10 nM and the DNA origami concentration is 5 nM in the gel.

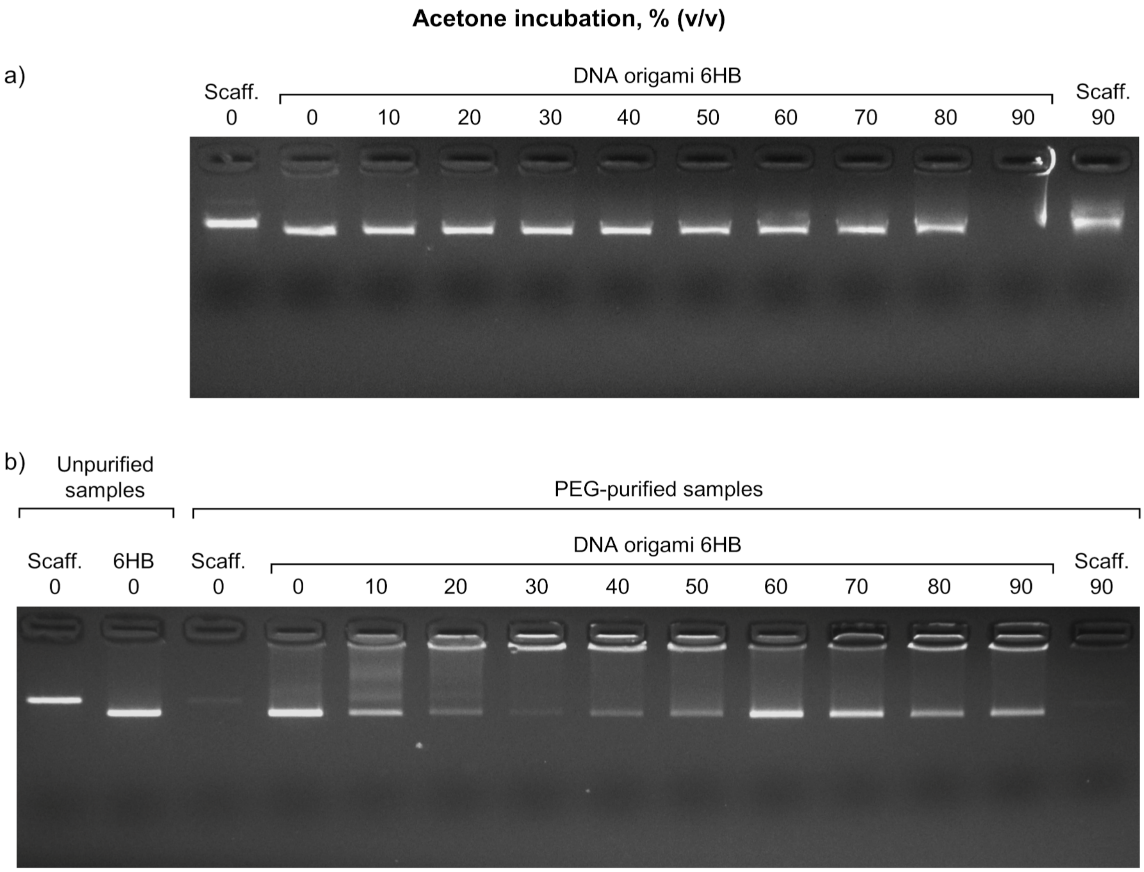

Figure S62. AGE of 6HB in deionized water exposed to acetone for 24 h at room temperature a) before and b) after removing acetone by PEG precipitation. The p7249 scaffold concentration is 10 nM and the DNA origami concentration is 5 nM in the gel.

5.3. 24HB in deionized water

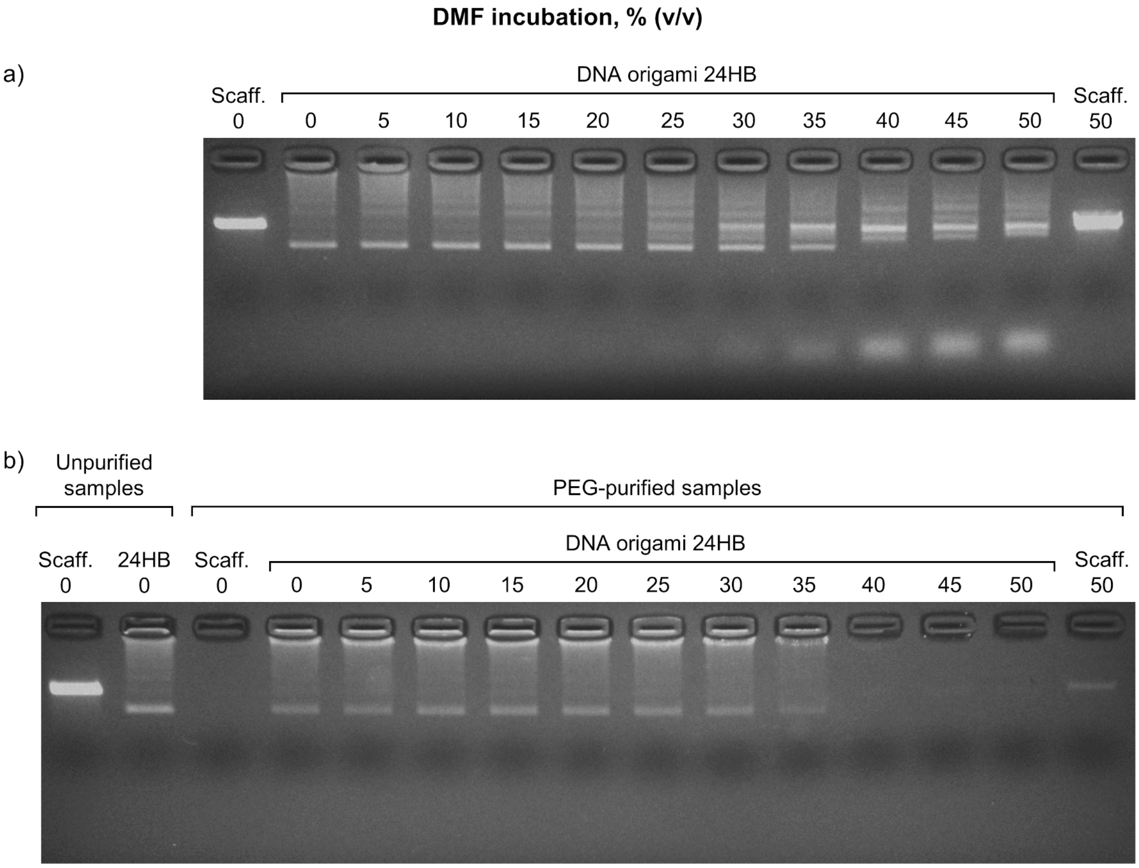

Figure S63. AGE of 24HB in deionized water exposed to DMF for 24 h at room temperature a) before and b) after removing DMF by PEG precipitation. The p7560 scaffold concentration is 10 nM and the DNA origami concentration is 5 nM in the gel.

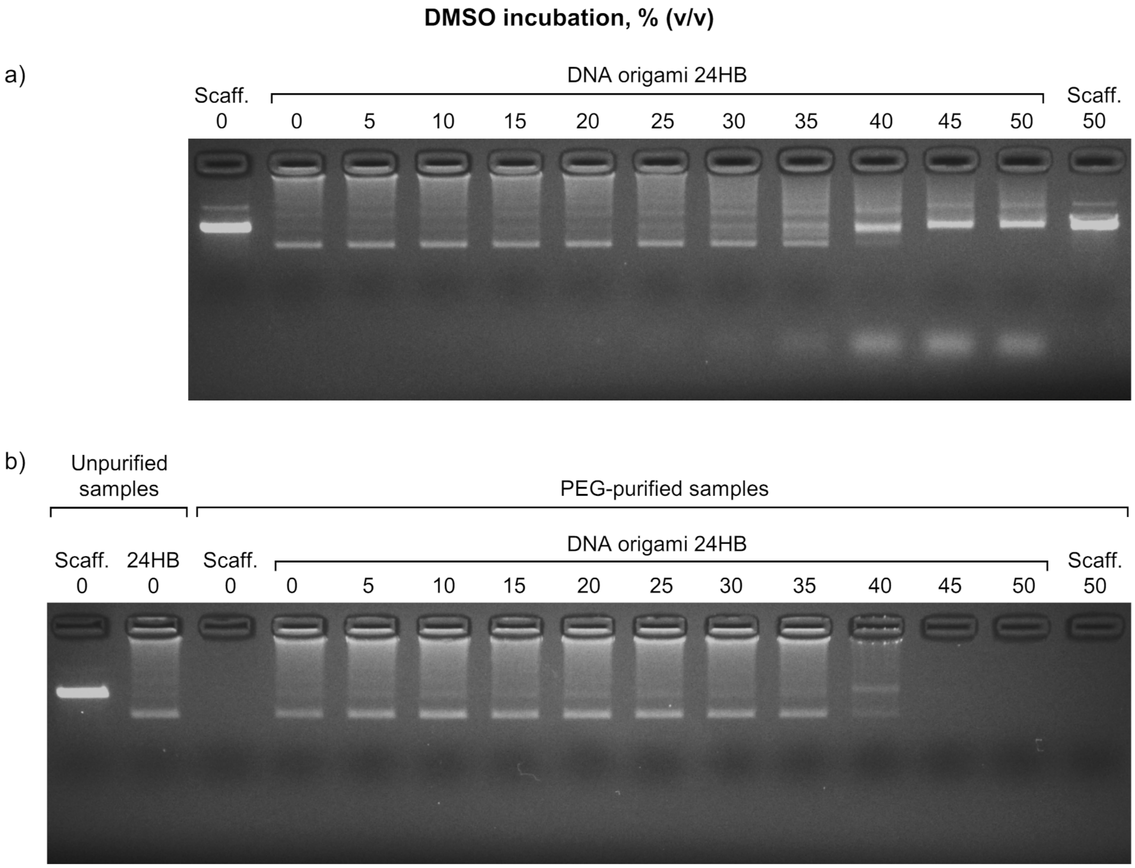

Figure S64. AGE of 24HB in deionized water exposed to DMSO for 24 h at room temperature a) before and b) after removing DMSO by PEG precipitation. The p7560 scaffold concentration is 10 nM and the DNA origami concentration is 5 nM in the gel.

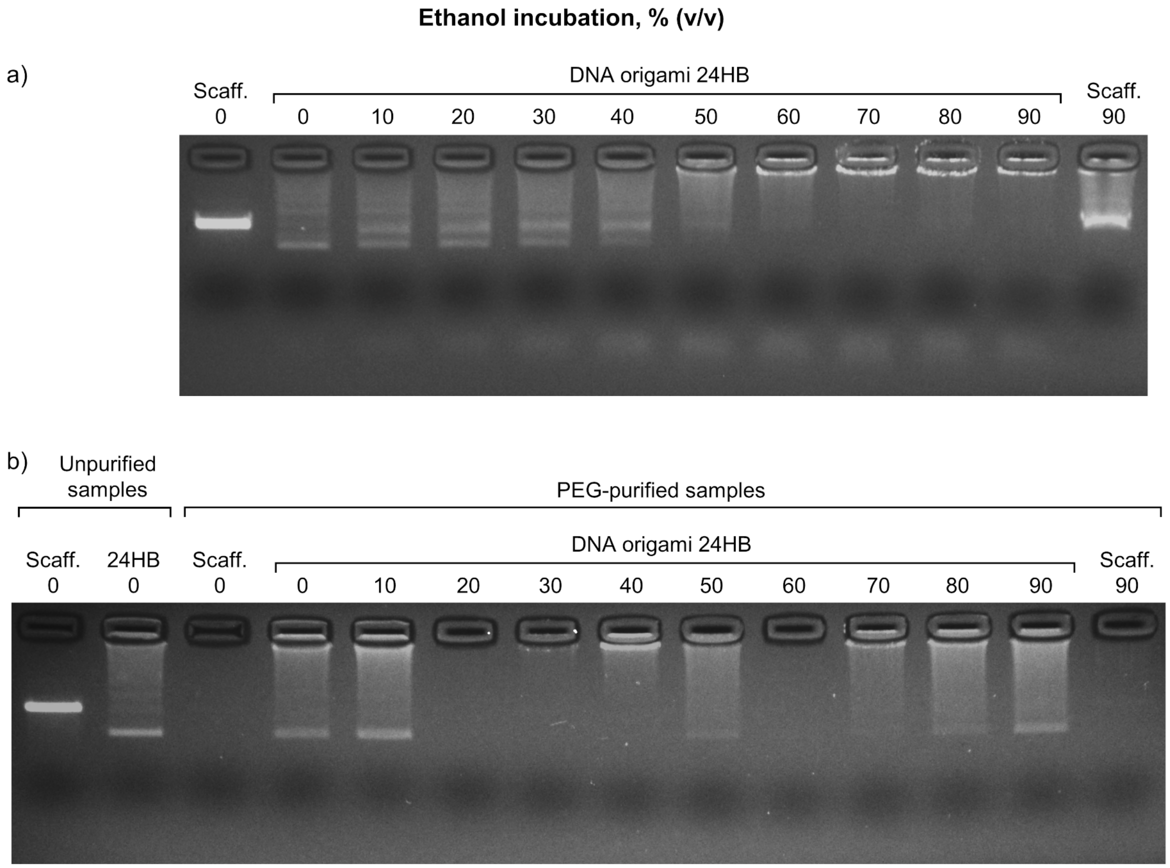

Figure S65. AGE of 24HB in deionized water exposed to ethanol for 24 h at room temperature a) before and b) after removing ethanol by PEG precipitation. The p7560 scaffold concentration is 10 nM and the DNA origami concentration is 5 nM in the gel.

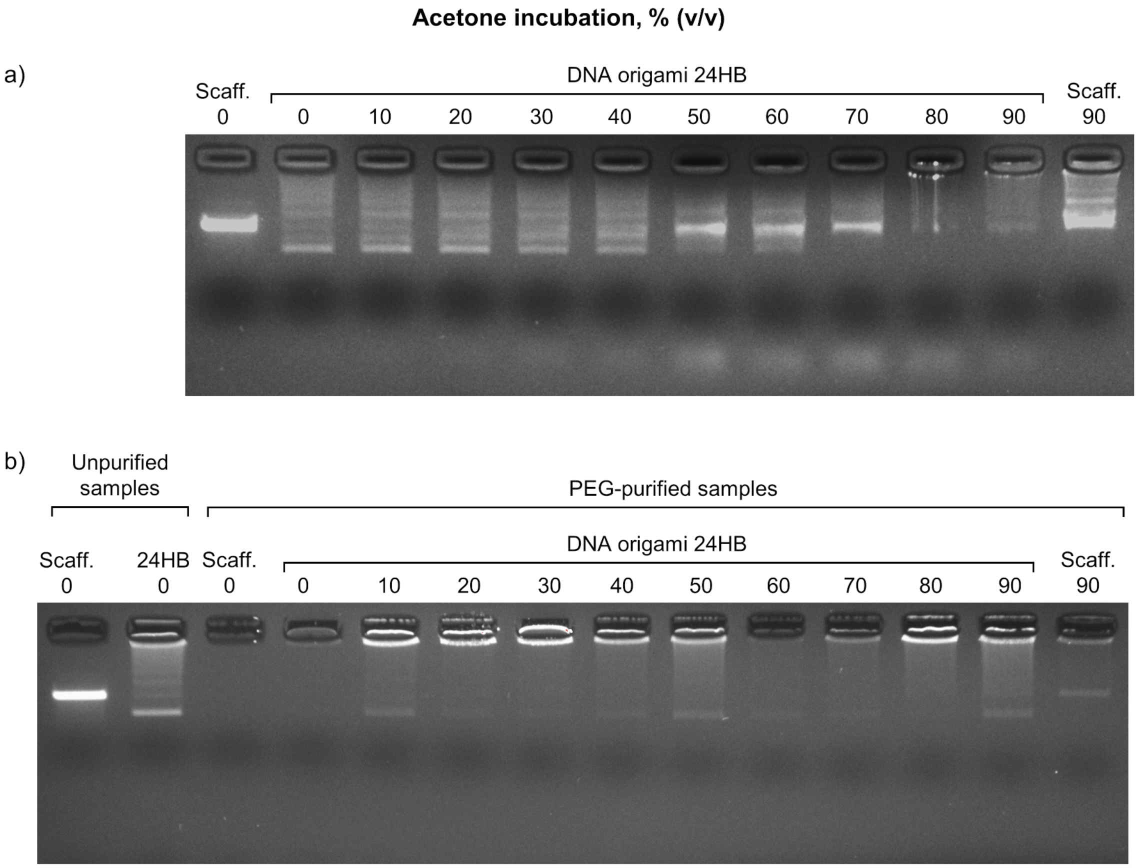

Figure S66. AGE of 24HB in deionized water exposed to acetone for 24 h at room temperature a) before and b) after removing acetone by PEG precipitation. The p7560 scaffold concentration is 10 nM and the DNA origami concentration is 5 nM in the gel.

6. Additional AFM images in deionized water

6.1. DNA origami triangle in deionized water exposed to DMF

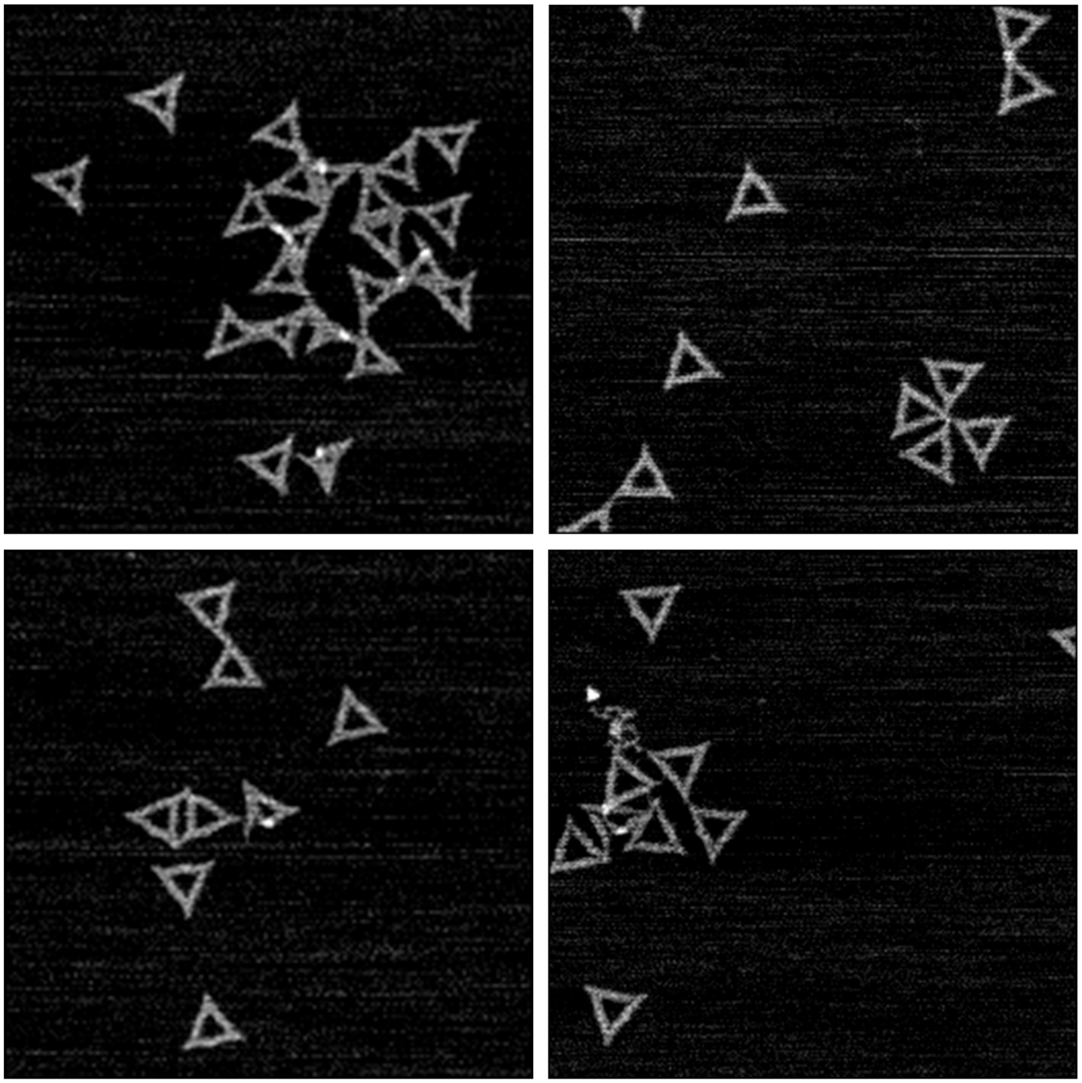

Figure S67. AFM images (1 μm × 1 μm) of DNA origami triangles in deionized water exposed to 25% (v/v) DMF for 24 h at room temperature. DMF was removed by PEG precipitation before the AFM sample preparation.

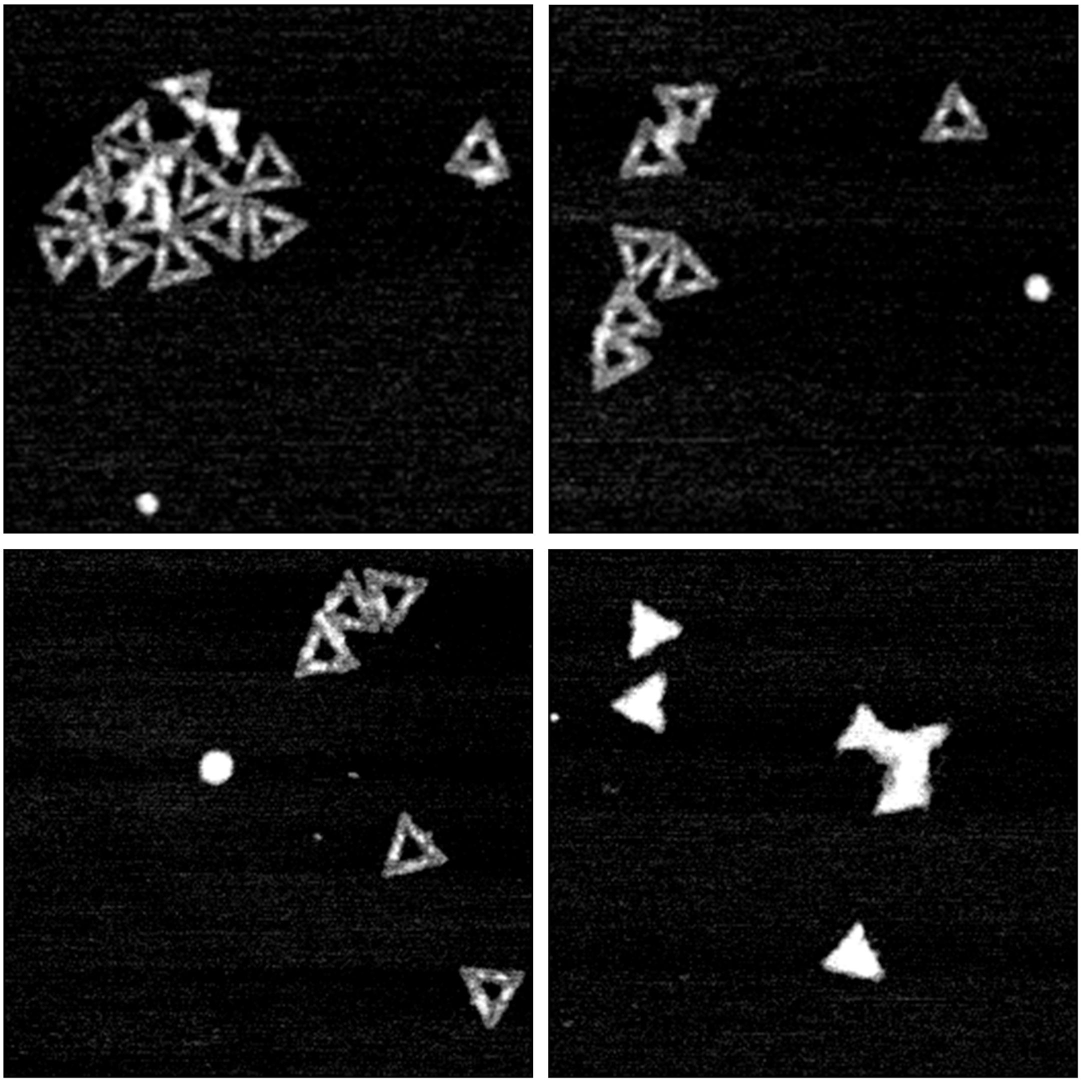

Figure S68. AFM images (1 μm × 1 μm) of DNA origami triangles in deionized water exposed to 30% (v/v) DMF for 24 h at room temperature. DMF was removed by PEG precipitation before the AFM sample preparation.

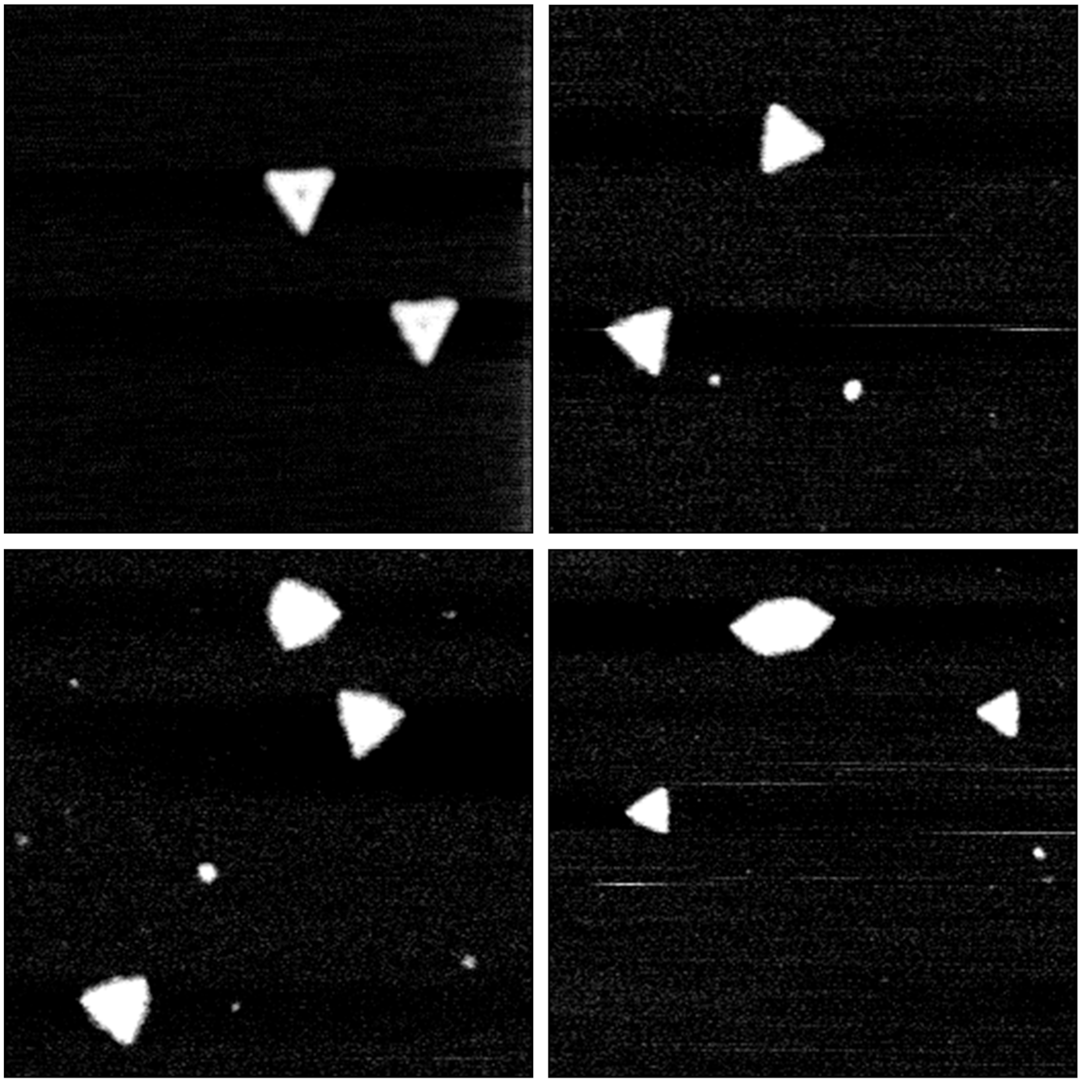

Figure S69. AFM images (1 μm × 1 μm) of DNA origami triangles in deionized water exposed to 35% (v/v) DMF for 24 h at room temperature. DMF was removed by PEG precipitation before the AFM sample preparation.

6.2. DNA origami triangle in deionized water exposed to DMSO

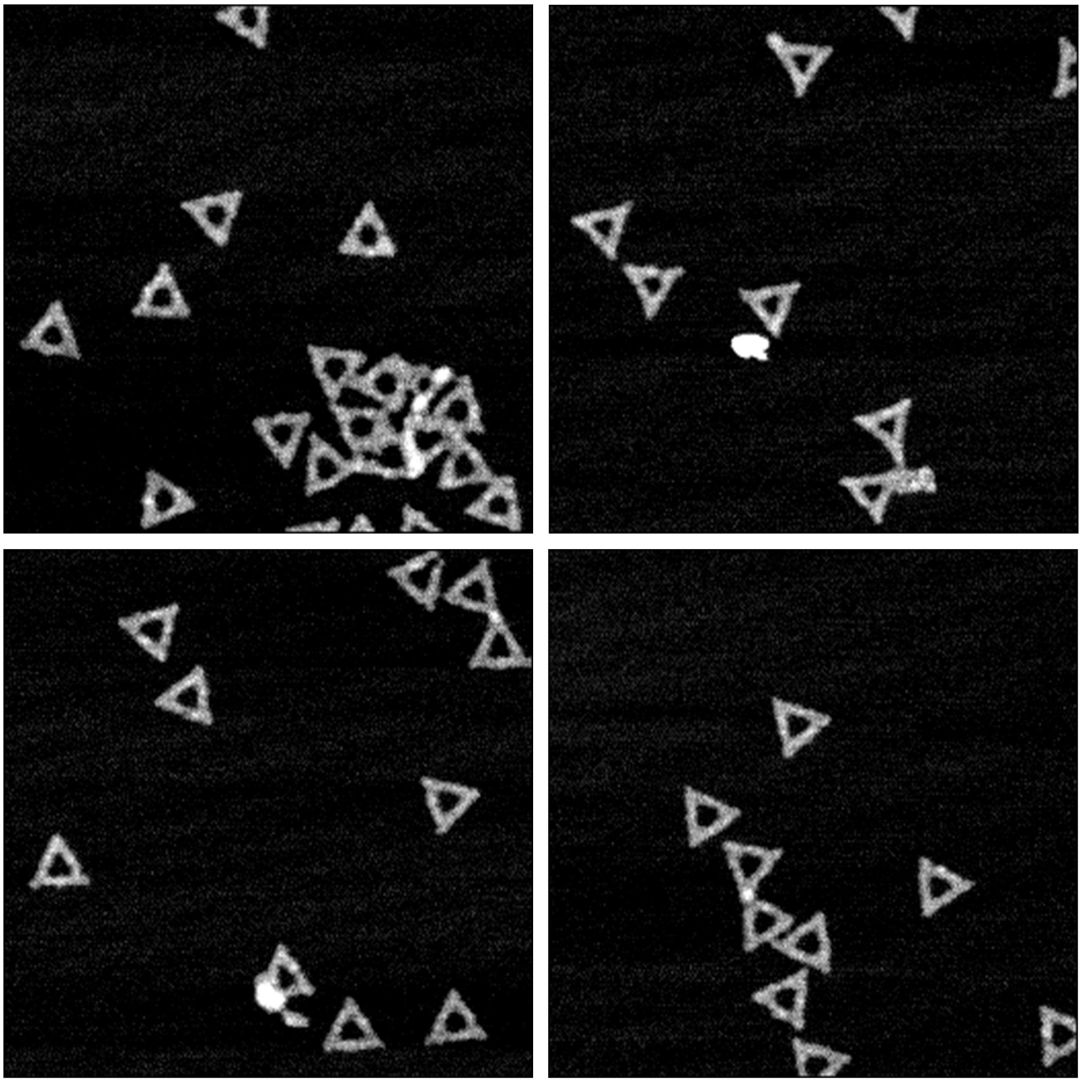

Figure S70. AFM images (1 μm × 1 μm) of DNA origami triangles in deionized water exposed to 25% (v/v) DMSO for 24 h at room temperature. DMSO was removed by PEG precipitation before the AFM sample preparation.

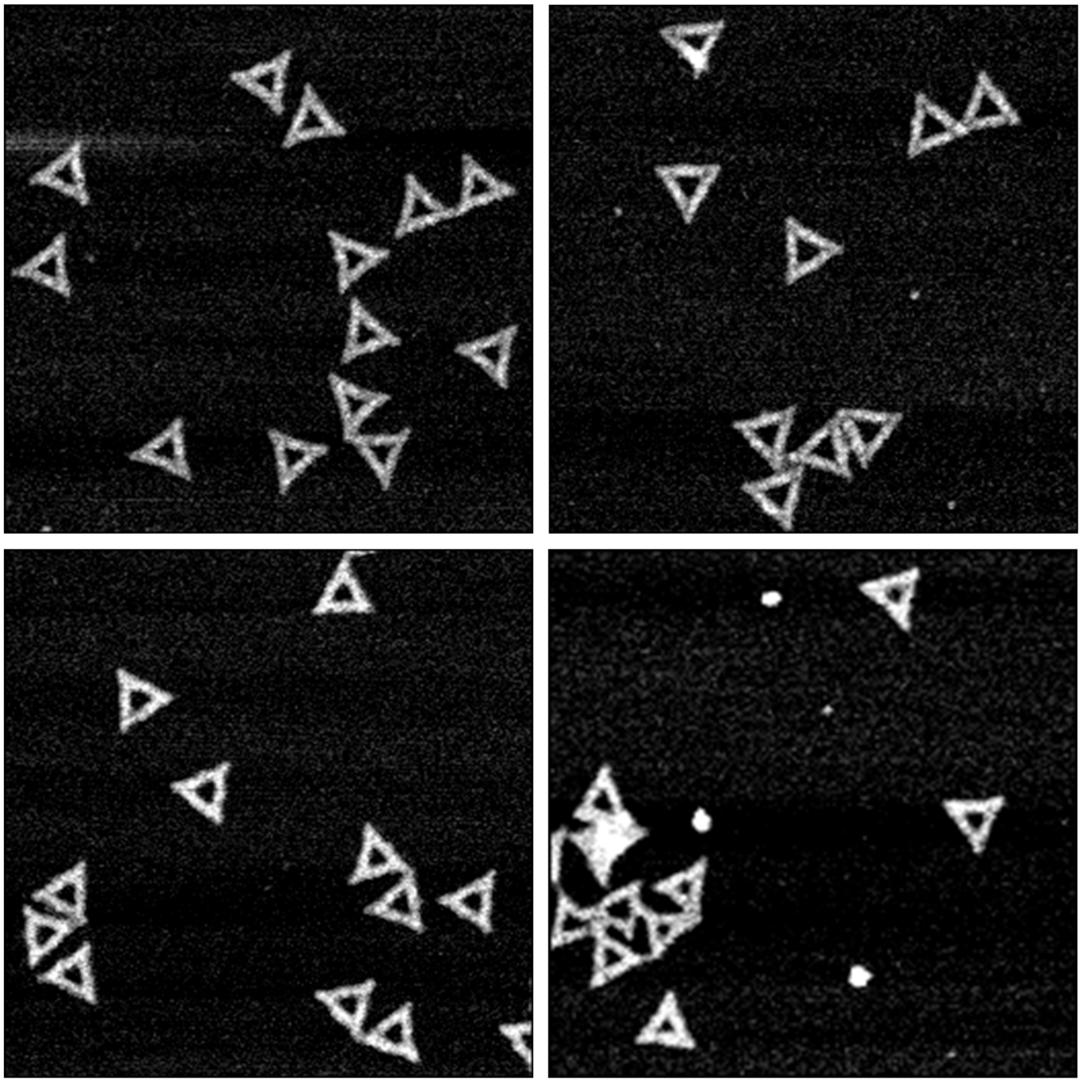

Figure S71. AFM images (1 μm × 1 μm) of DNA origami triangles in deionized water exposed to 30% (v/v) DMSO for 24 h at room temperature. DMSO was removed by PEG precipitation before the AFM sample preparation.

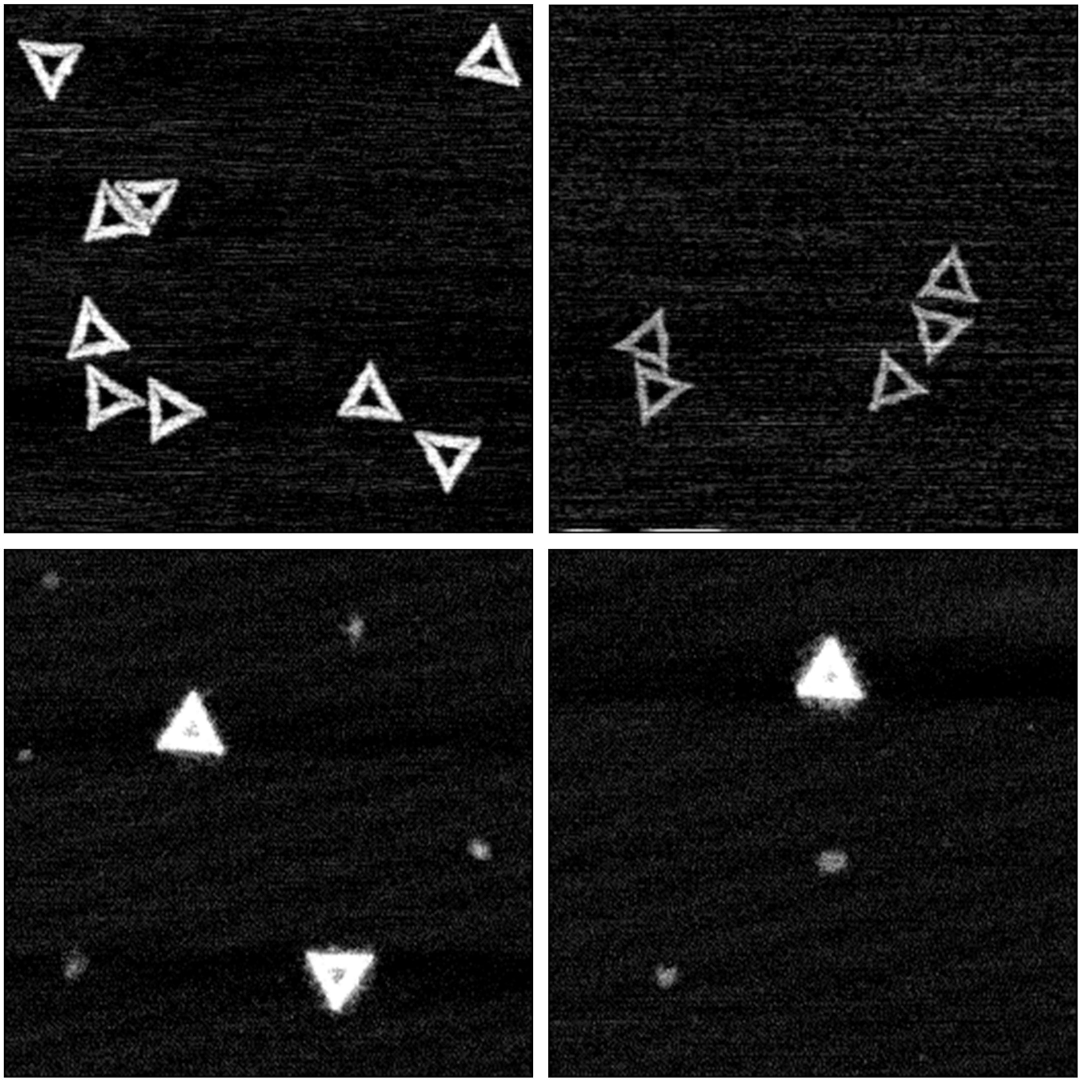

Figure S72. AFM images (1 μm × 1 μm) of DNA origami triangles in deionized water exposed to 35% (v/v) DMSO for 24 h at room temperature. DMSO was removed by PEG precipitation before the AFM sample preparation.

6.3. DNA origami triangle in deionized water exposed to ethanol

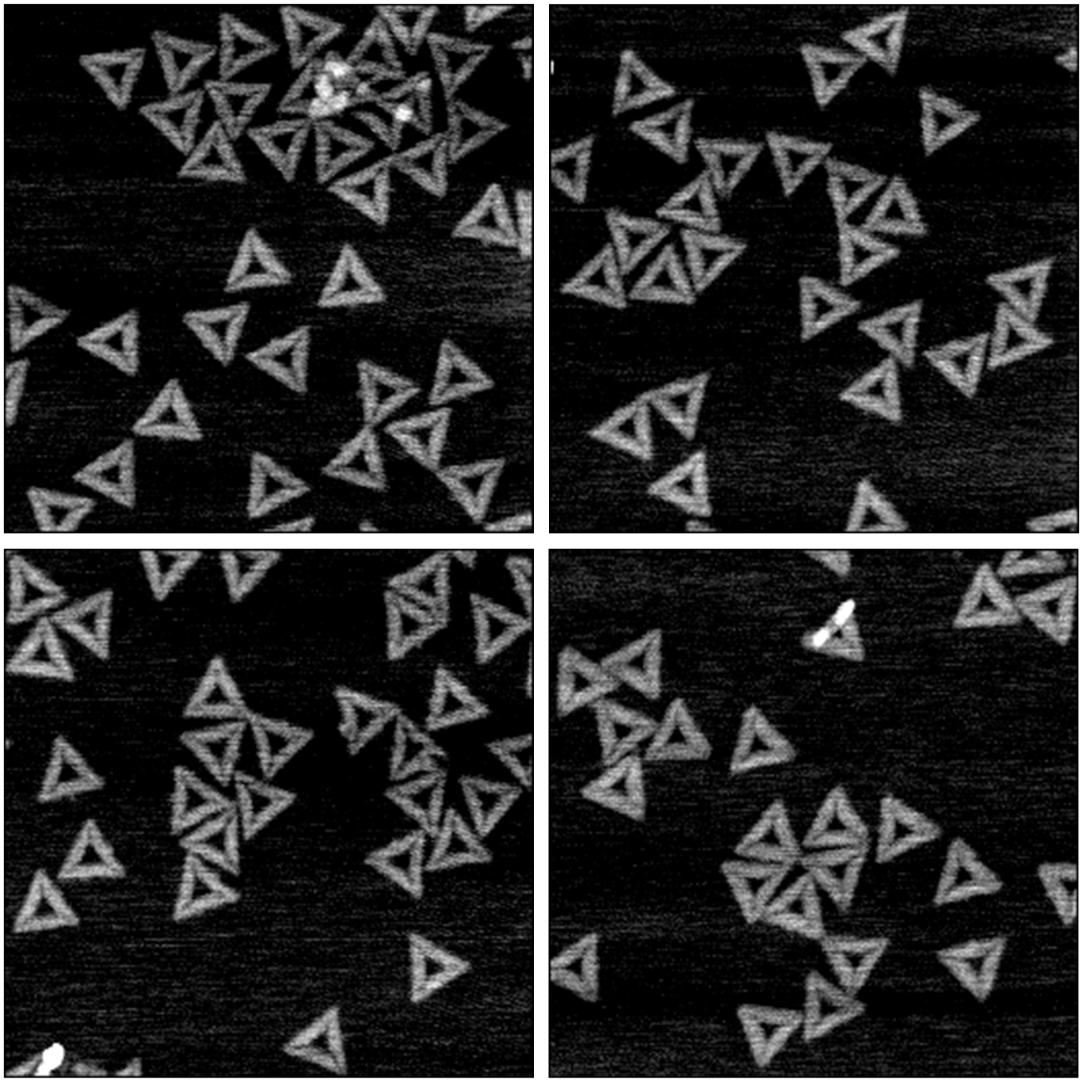

Figure S73. AFM images (1 μm × 1 μm) of DNA origami triangles in deionized water exposed to 10% (v/v) ethanol for 24 h at room temperature. Ethanol was removed by PEG precipitation before the AFM sample preparation.

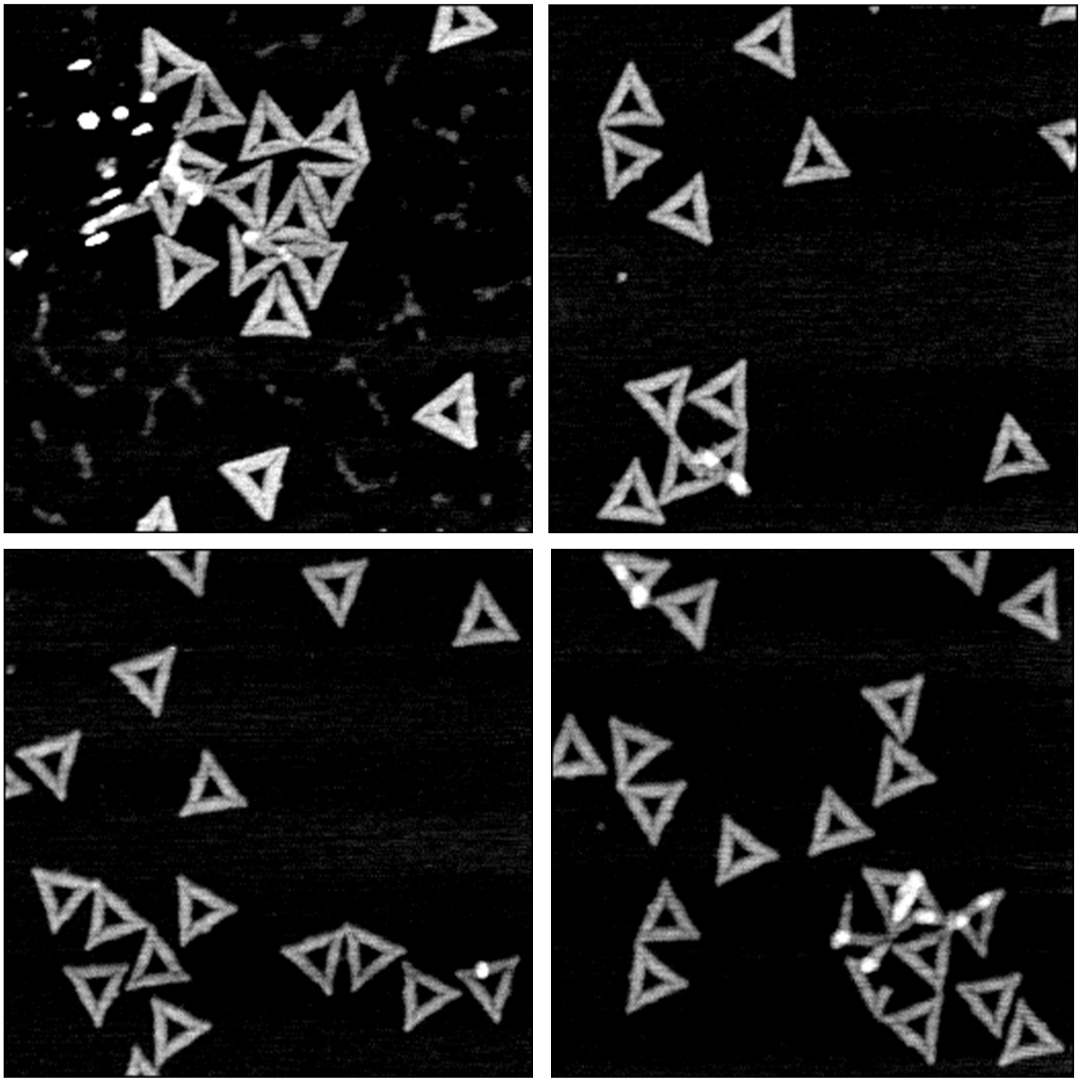

Figure S74. AFM images (1 μm × 1 μm) of DNA origami triangles in deionized water exposed to 40% (v/v) ethanol for 24 h at room temperature. Ethanol was removed by PEG precipitation before the AFM sample preparation.

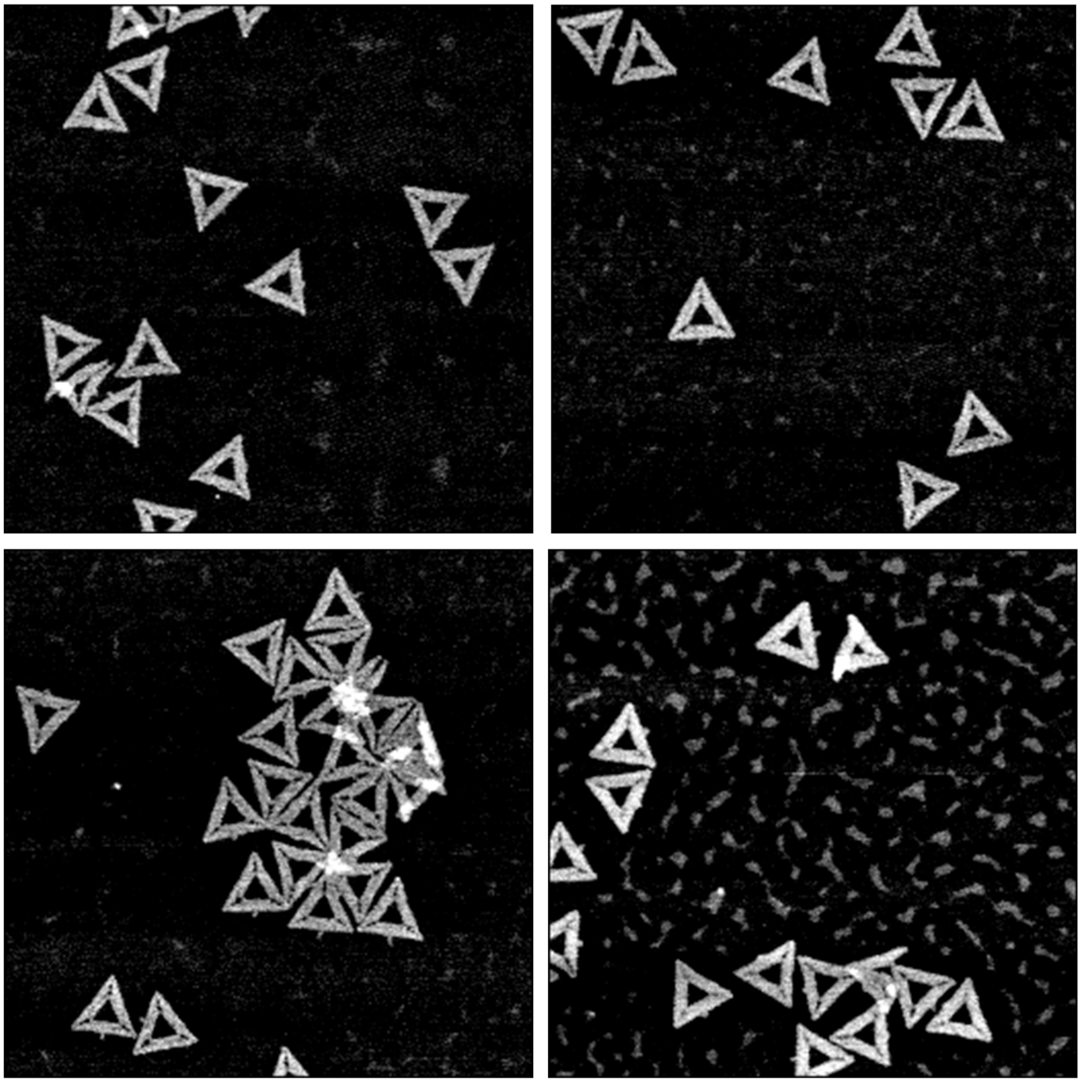

Figure S75. AFM images (1 μm × 1 μm) of DNA origami triangles in deionized water exposed to 90% (v/v) ethanol for 24 h at room temperature. Ethanol was removed by PEG precipitation before the AFM sample preparation.

6.4. DNA origami triangle in deionized water exposed to acetone

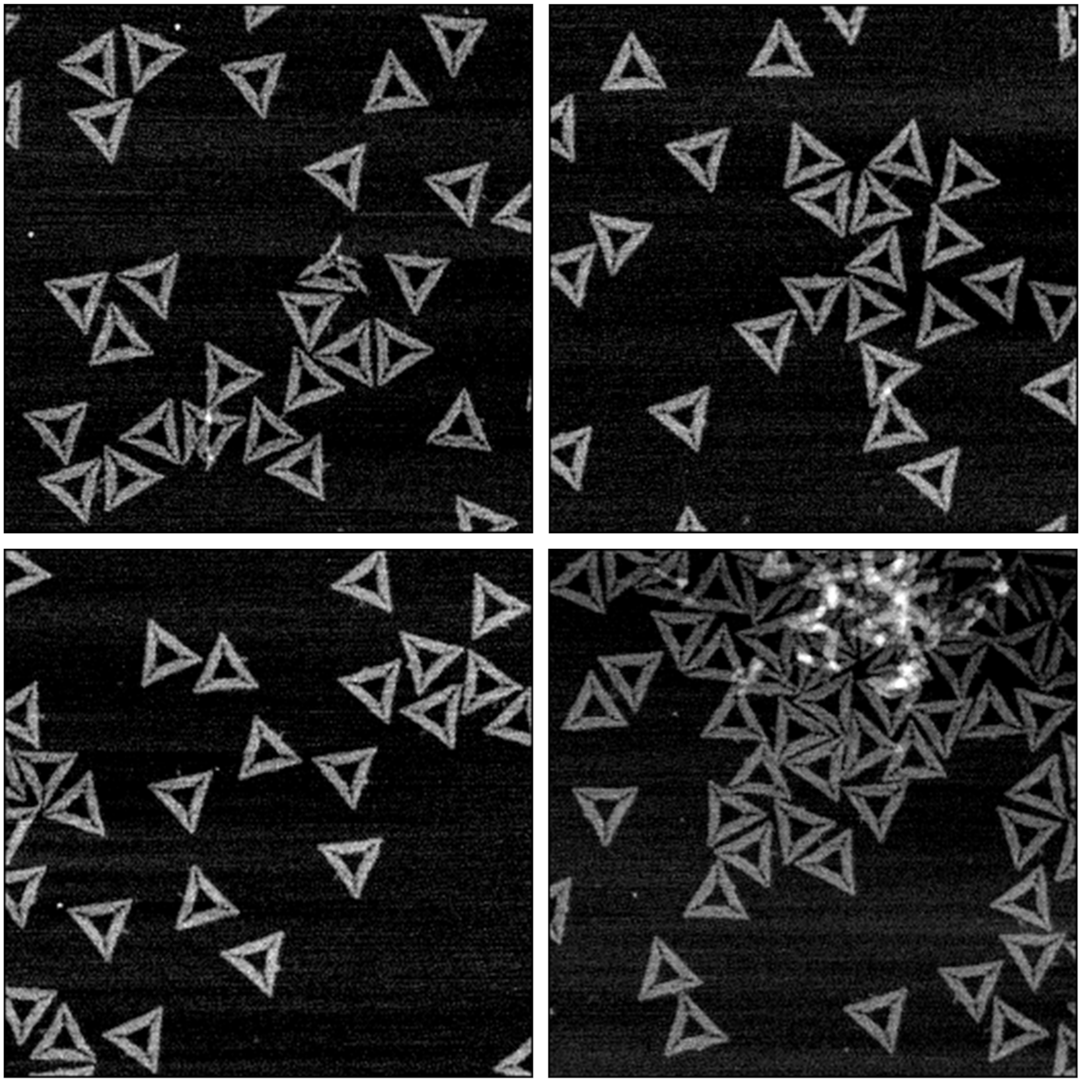

Figure S76. AFM images (1 μm × 1 μm) of DNA origami triangles in deionized water exposed to 10% (v/v) acetone for 24 h at room temperature. Acetone was removed by PEG precipitation before the AFM sample preparation.

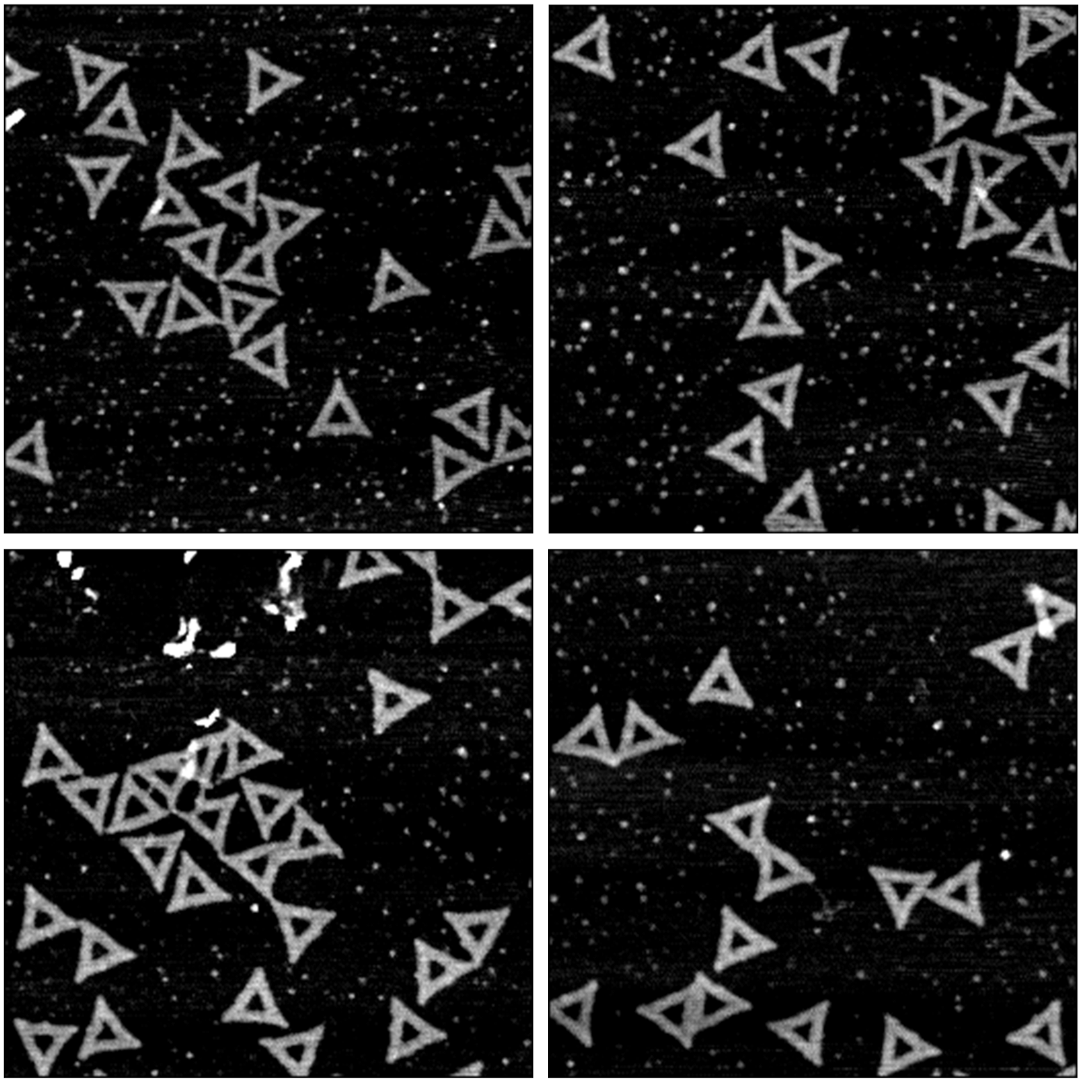

Figure S77. AFM images (1 μm × 1 μm) of DNA origami triangles in deionized water exposed to 40% (v/v) acetone for 24 h at room temperature. Acetone was removed by PEG precipitation before the AFM sample preparation.

7. Additional TEM images in deionized water

7.1. 6HB in deionized water exposed to DMF

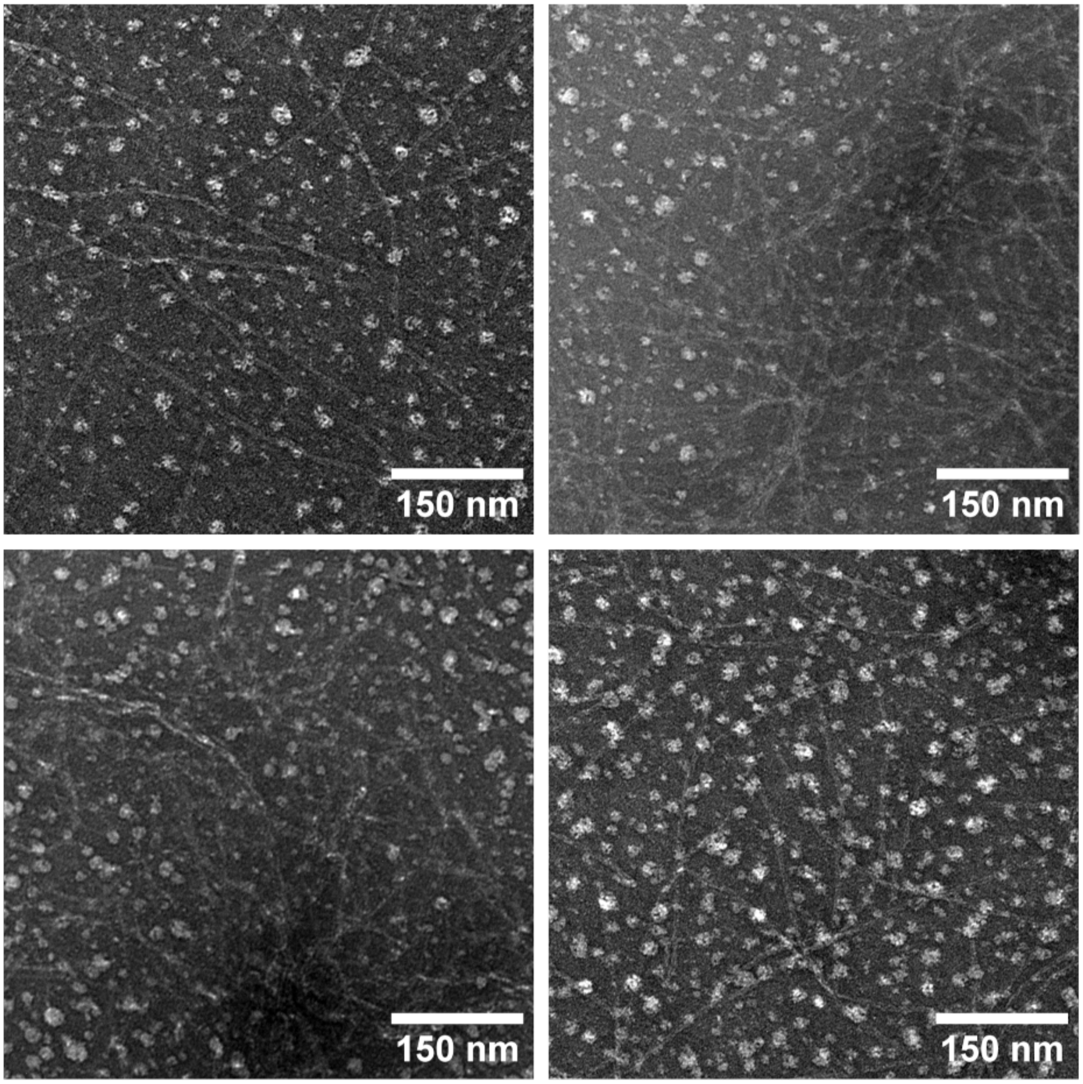

Figure S78. TEM images of 6HB in deionized water exposed to 10% (v/v) DMF for 24 h at room temperature. The TEM samples are negatively stained with uranyl formate (2% (w/v)). DMF was removed by PEG precipitation before the TEM sample preparation.

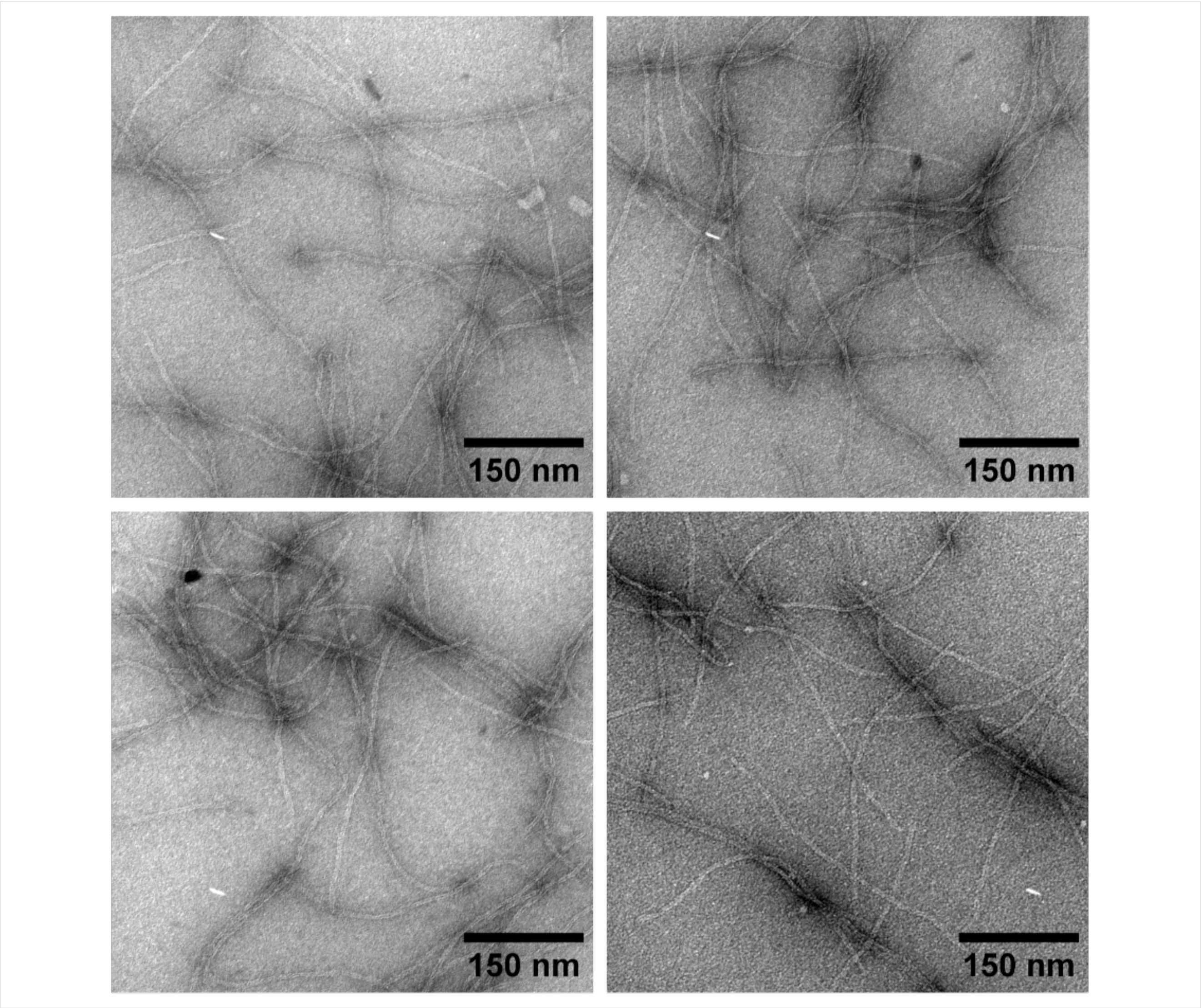

Figure S79. TEM images of 6HB in deionized water exposed to 35% (v/v) DMF for 24 h at room temperature. The TEM samples are negatively stained with uranyl formate (2% (w/v)). DMF was removed by PEG precipitation before the TEM sample preparation.

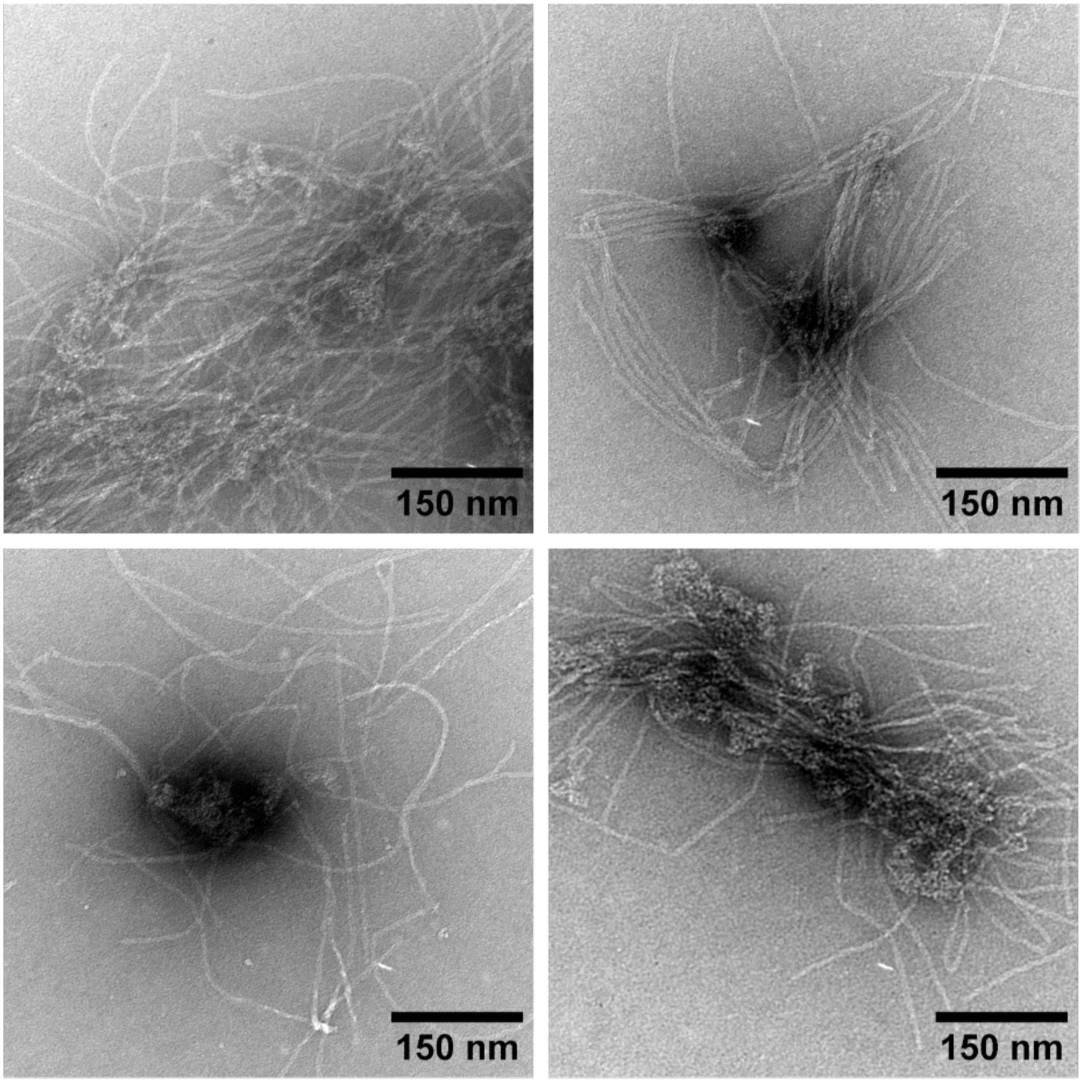

Figure S80. TEM images of 6HB in deionized water exposed to 40% (v/v) DMF for 24 h at room temperature. The TEM samples are negatively stained with uranyl formate (2% (w/v)). DMF was removed by PEG precipitation before the TEM sample preparation.

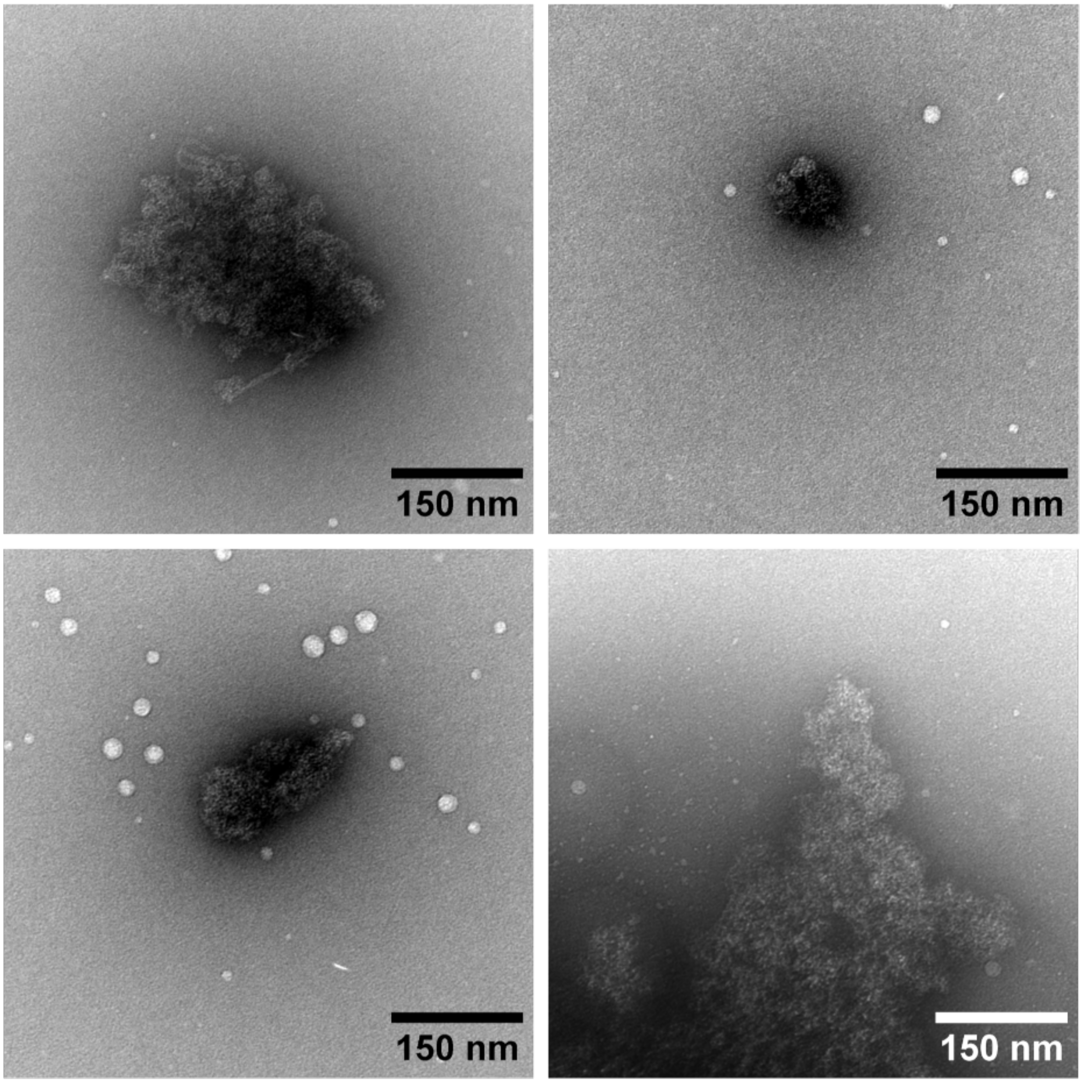

Figure S81. TEM images of 6HB in deionized water exposed to 45% (v/v) DMF for 24 h at room temperature. The TEM samples are negatively stained with uranyl formate (2% (w/v)). DMF was removed by PEG precipitation before the TEM sample preparation.

7.2. 6HB in deionized water exposed to DMSO

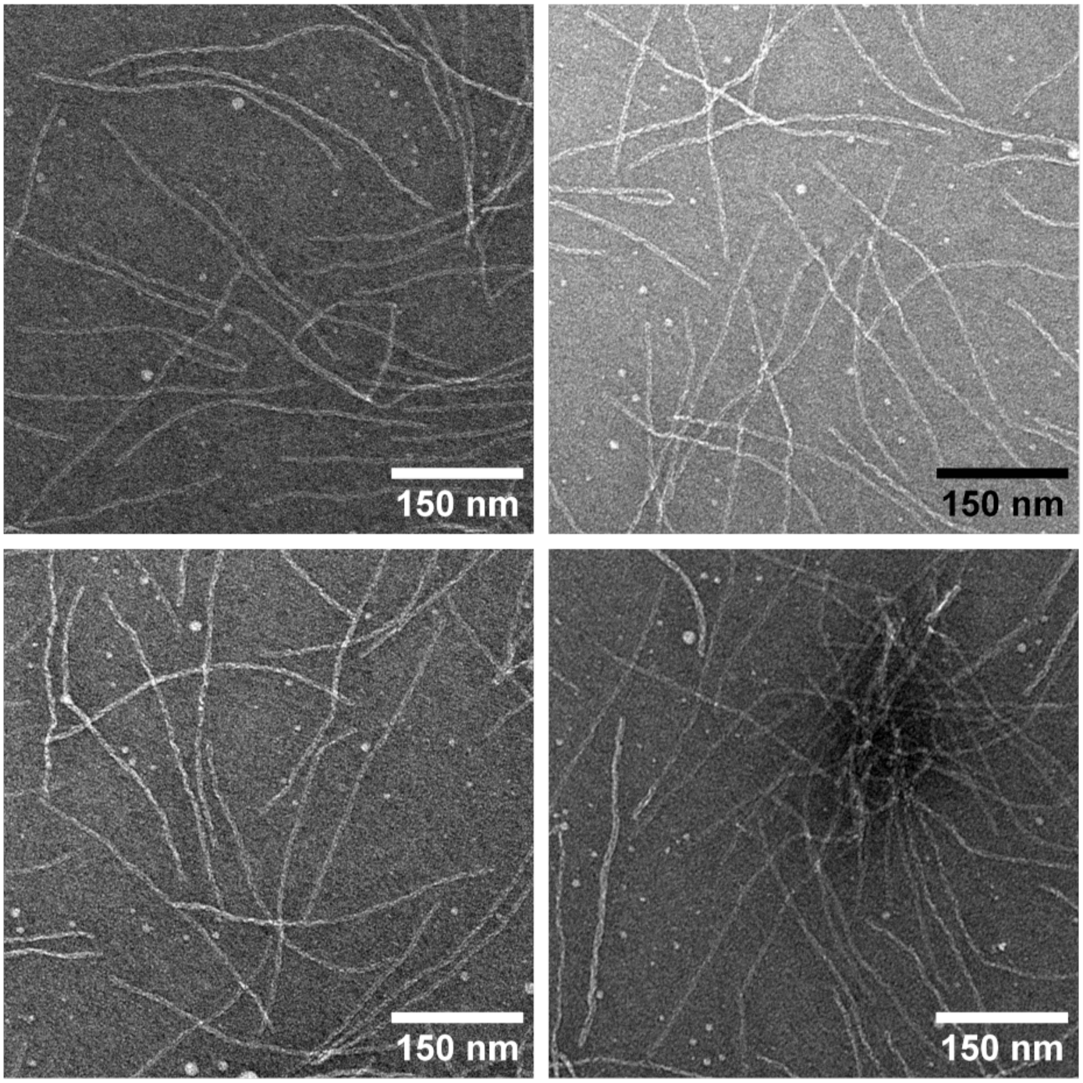

Figure S82. TEM images of 6HB in deionized water exposed to 10% (v/v) DMSO for 24 h at room temperature. The TEM samples are negatively stained with uranyl formate (2% (w/v)). DMSO was removed by PEG precipitation before the TEM sample preparation.

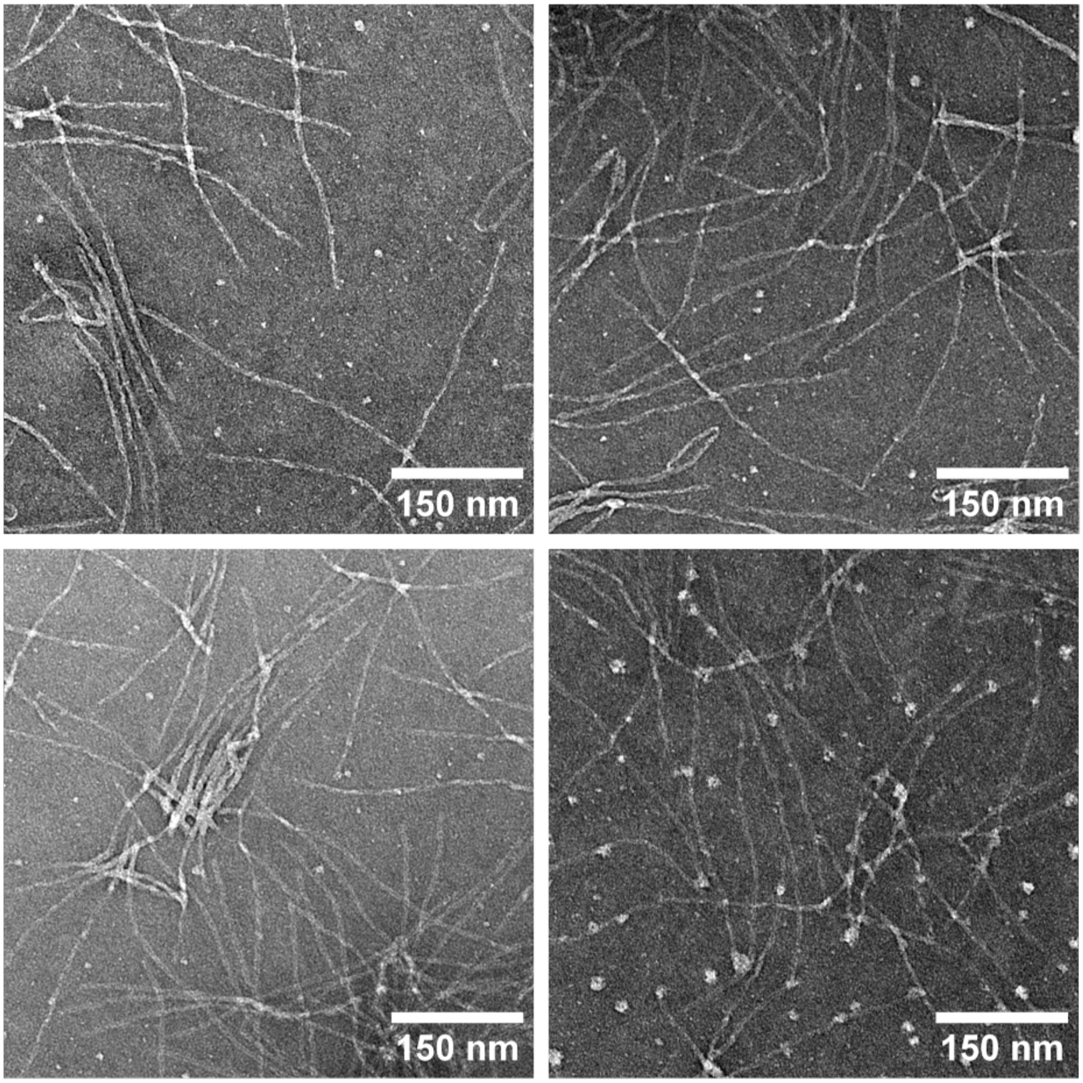

Figure S83. TEM images of 6HB in deionized water exposed to 30% (v/v) DMSO for 24 h at room temperature. The TEM samples are negatively stained with uranyl formate (2% (w/v)). DMSO was removed by PEG precipitation before the TEM sample preparation.

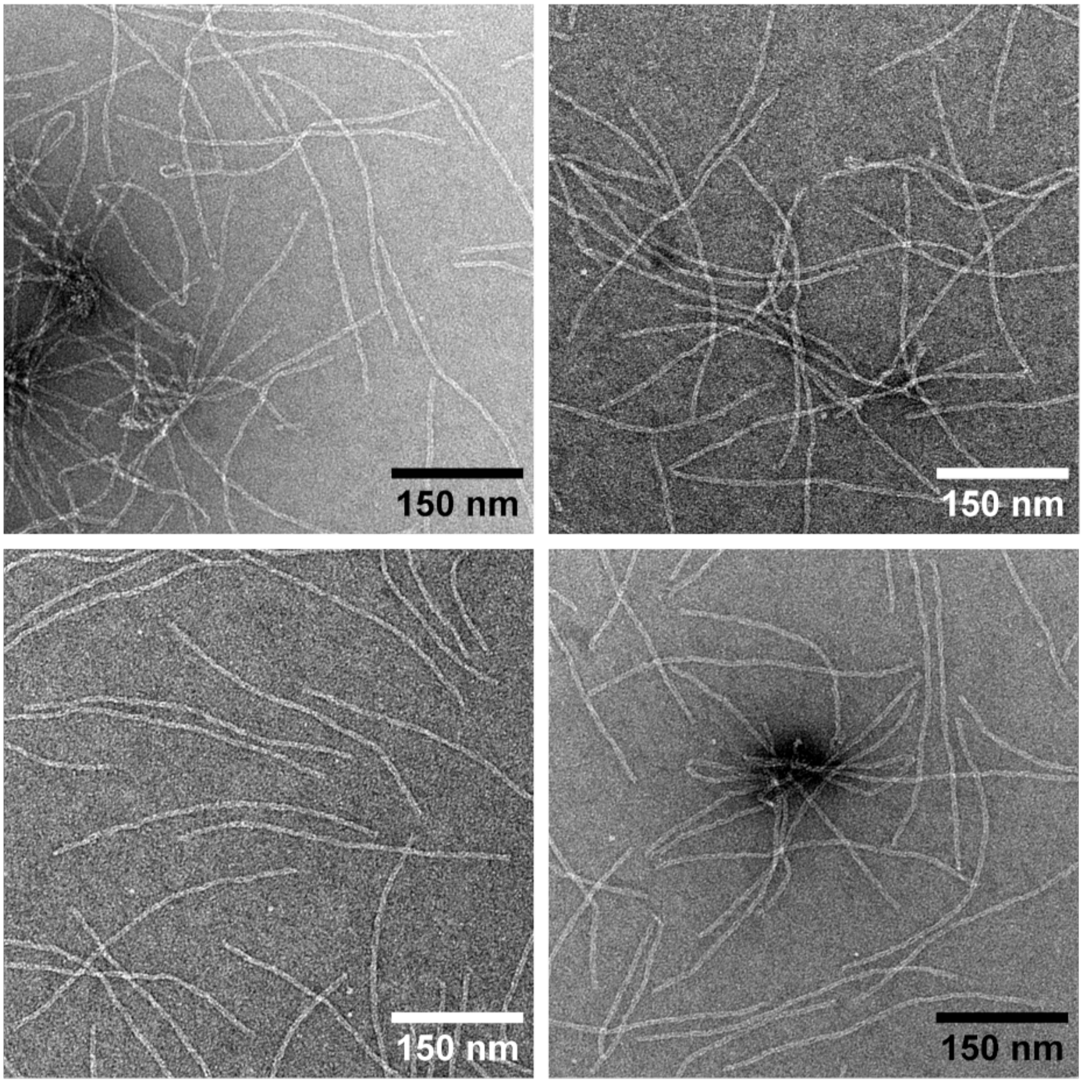

Figure S84. TEM images of 6HB in deionized water exposed to 35% (v/v) DMSO for 24 h at room temperature. The TEM samples are negatively stained with uranyl formate (2% (w/v)). DMSO was removed by PEG precipitation before the TEM sample preparation.

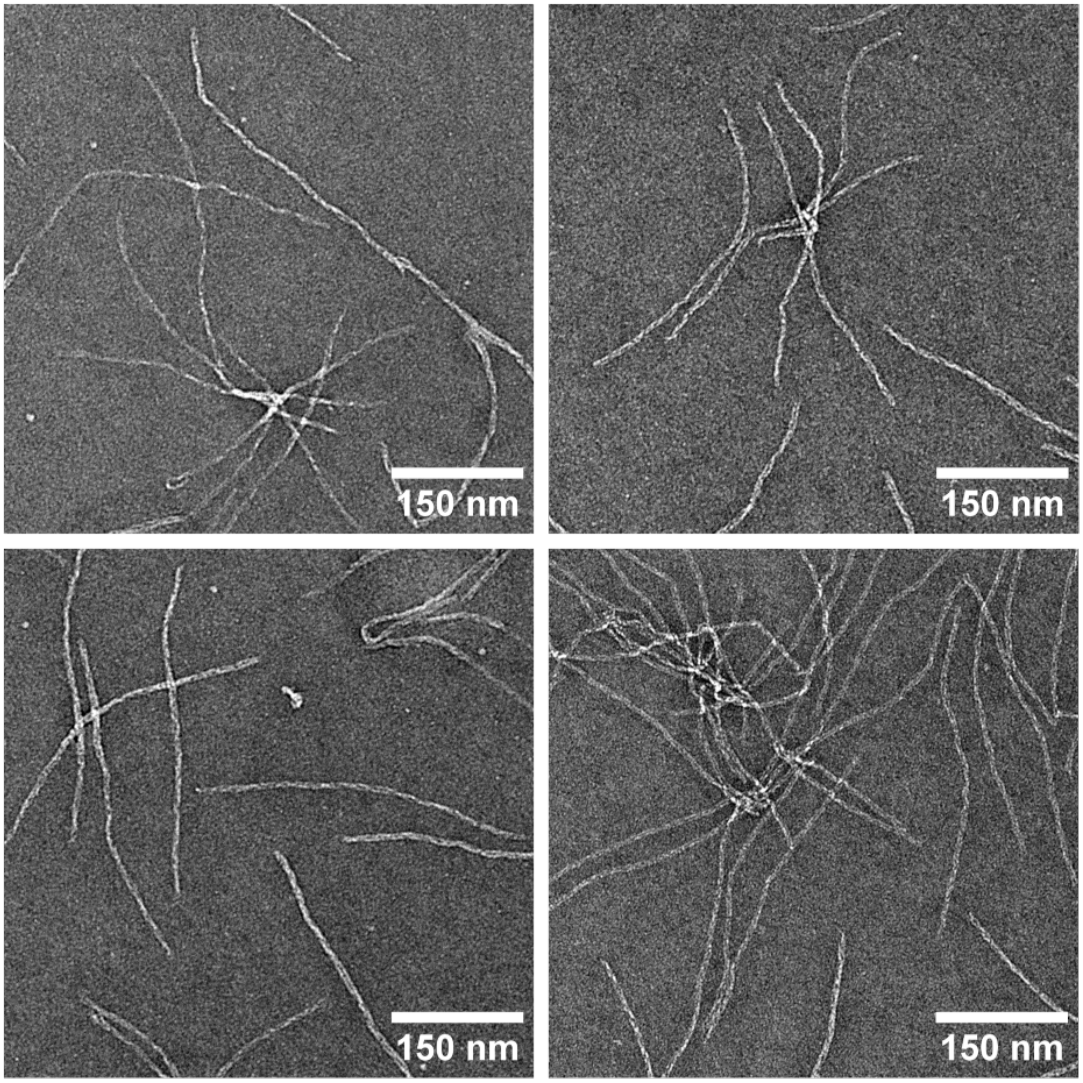

Figure S85. TEM images of 6HB in deionized water exposed to 40% (v/v) DMSO for 24 h at room temperature. The TEM samples are negatively stained with uranyl formate (2% (w/v)). DMSO was removed by PEG precipitation before the TEM sample preparation.

7.3. 6HB in deionized water exposed to ethanol

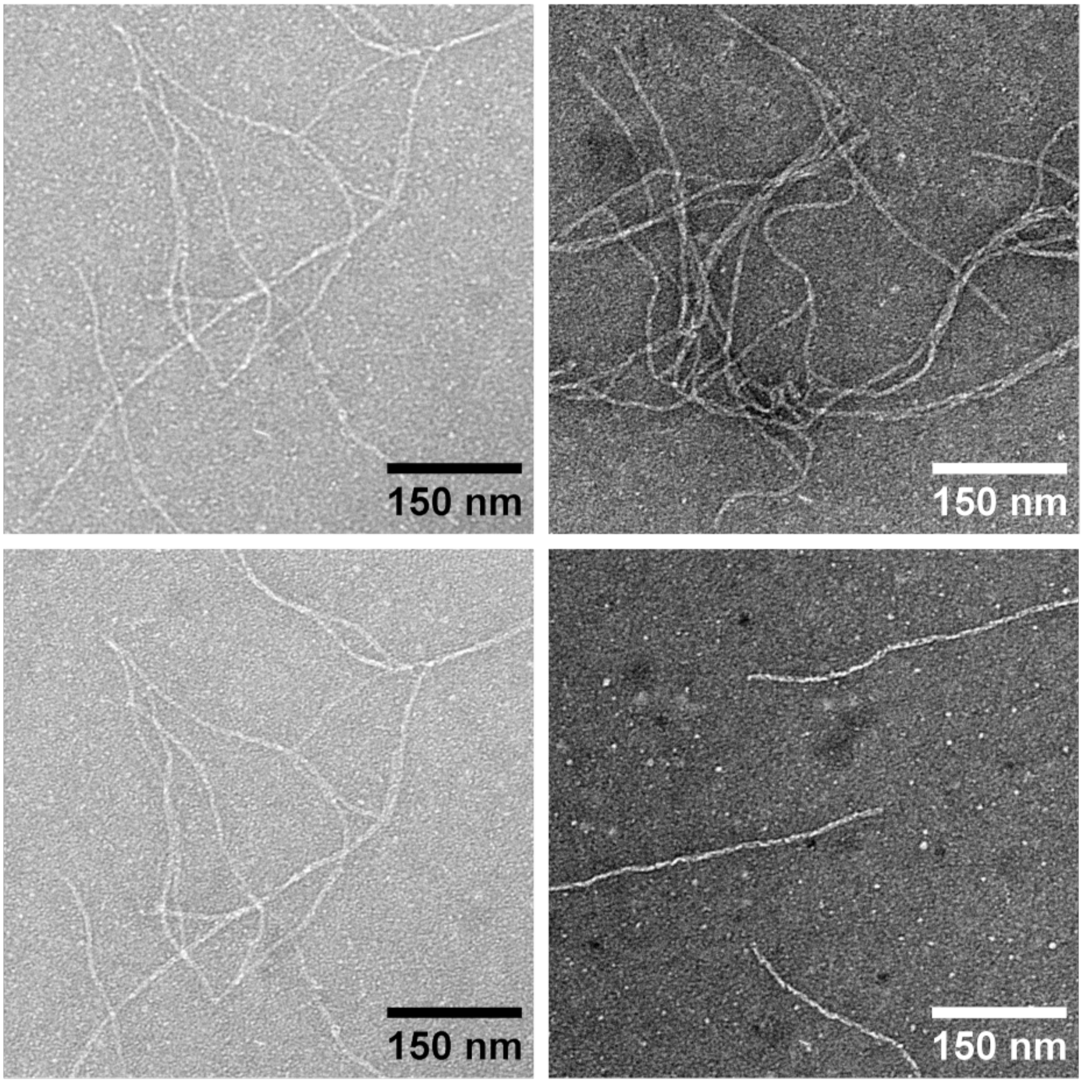

Figure S86. TEM images of 6HB in deionized water exposed to 10% (v/v) ethanol for 24 h at room temperature. The TEM samples are negatively stained with uranyl formate (2% (w/v)). Ethanol was removed by PEG precipitation before the TEM sample preparation.

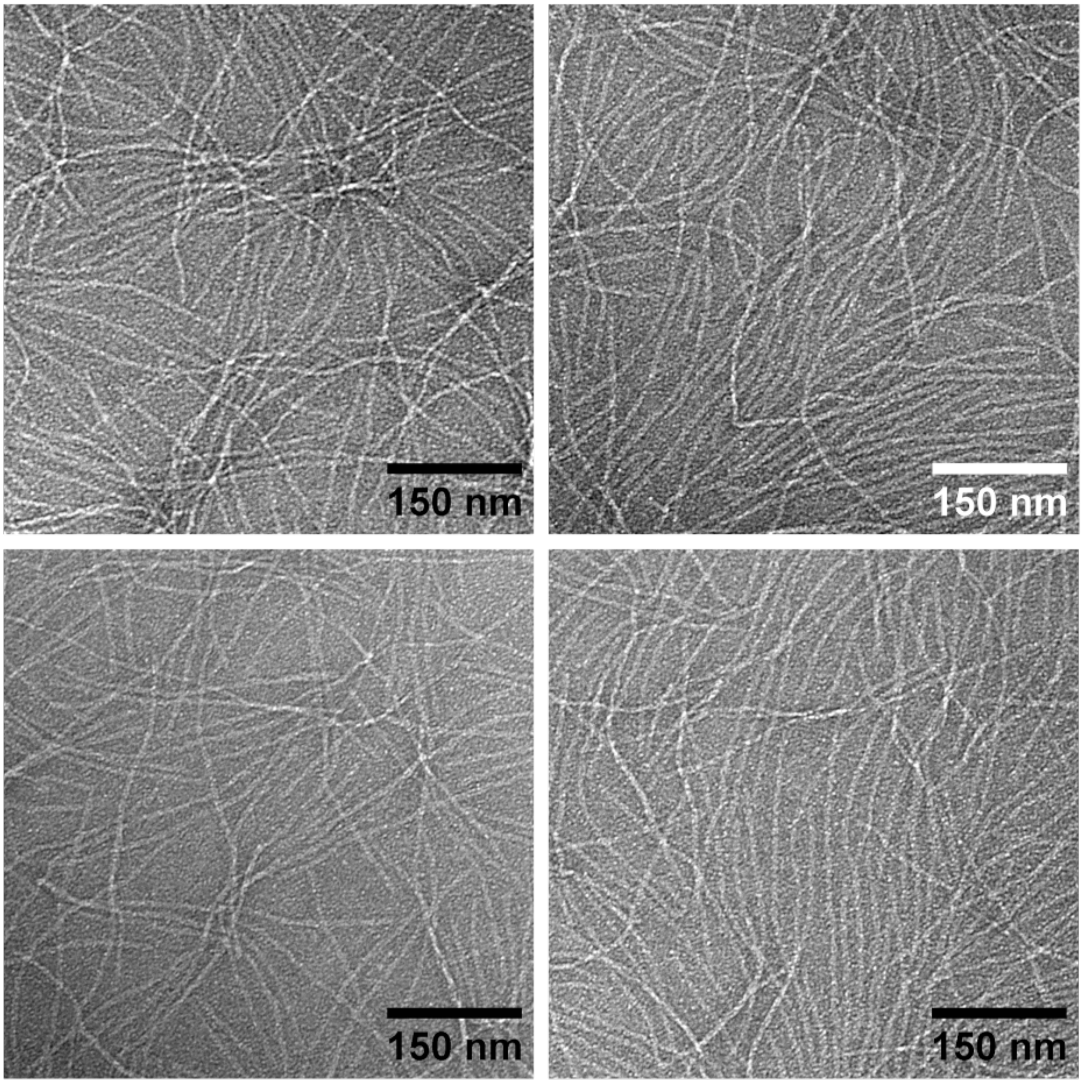

Figure S87. TEM images of 6HB in deionized water exposed to 40% (v/v) ethanol for 24 h at room temperature. The TEM samples are negatively stained with uranyl formate (2% (w/v)). Ethanol was removed by PEG precipitation before the TEM sample preparation.

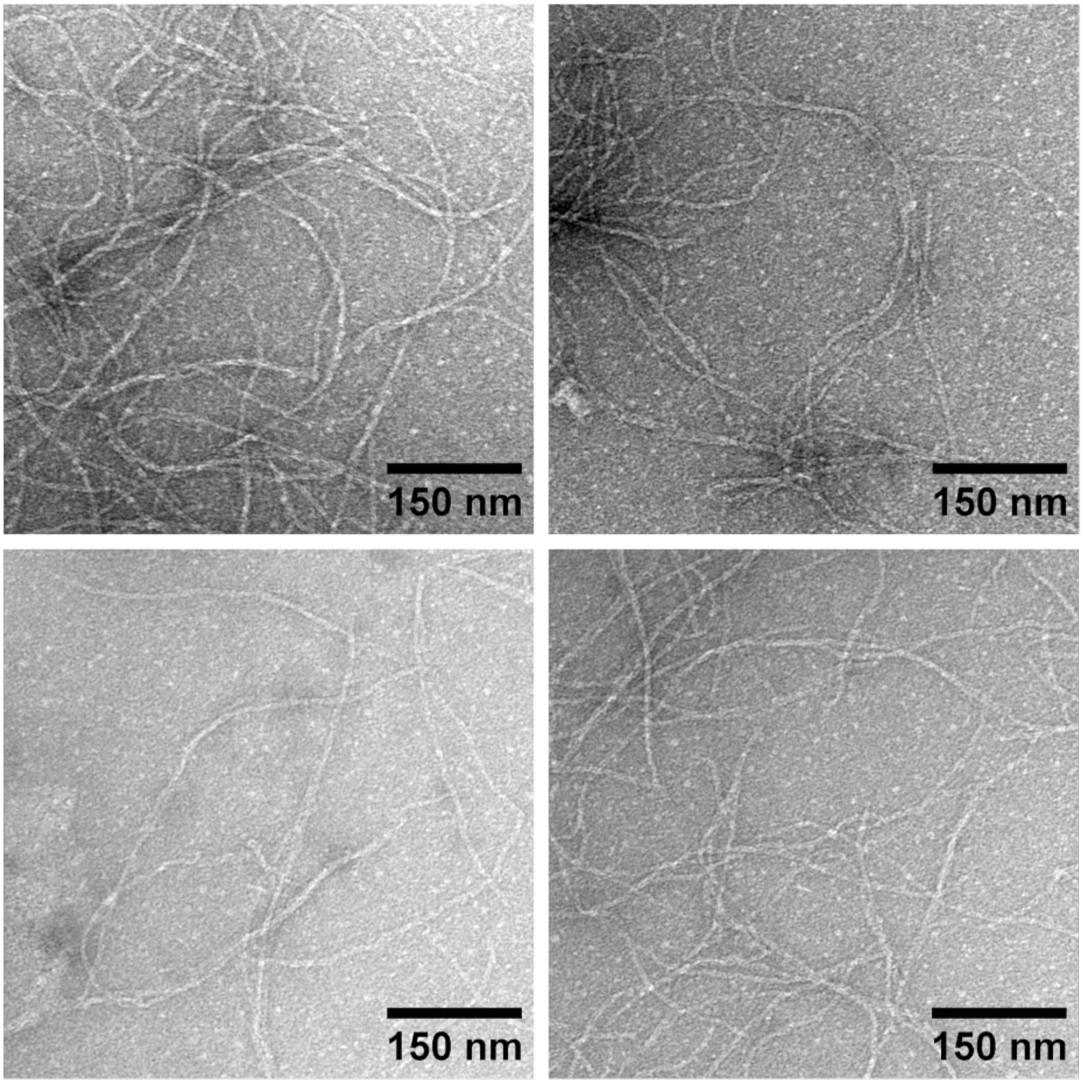

Figure S88. TEM images of 6HB in deionized water exposed to 90% (v/v) ethanol for 24 h at room temperature. The TEM samples are negatively stained with uranyl formate (2% (w/v)). Ethanol was removed by PEG precipitation before the TEM sample preparation.

7.4. 6HB in deionized water exposed to acetone

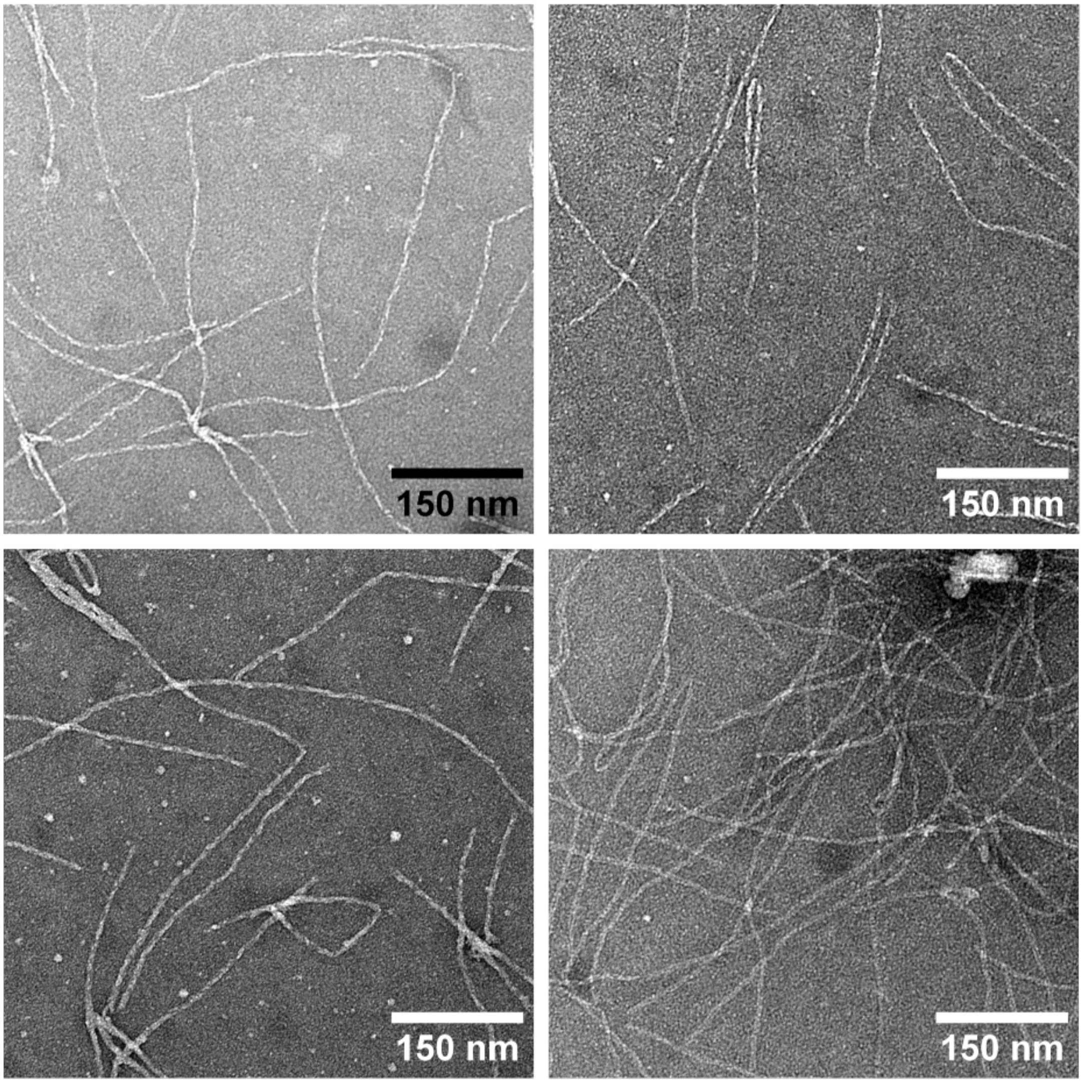

Figure S89. TEM images of 6HB in deionized water exposed to 10% (v/v) acetone for 24 h at room temperature. The TEM samples are negatively stained with uranyl formate (2% (w/v)). Acetone was removed by PEG precipitation before the TEM sample preparation.

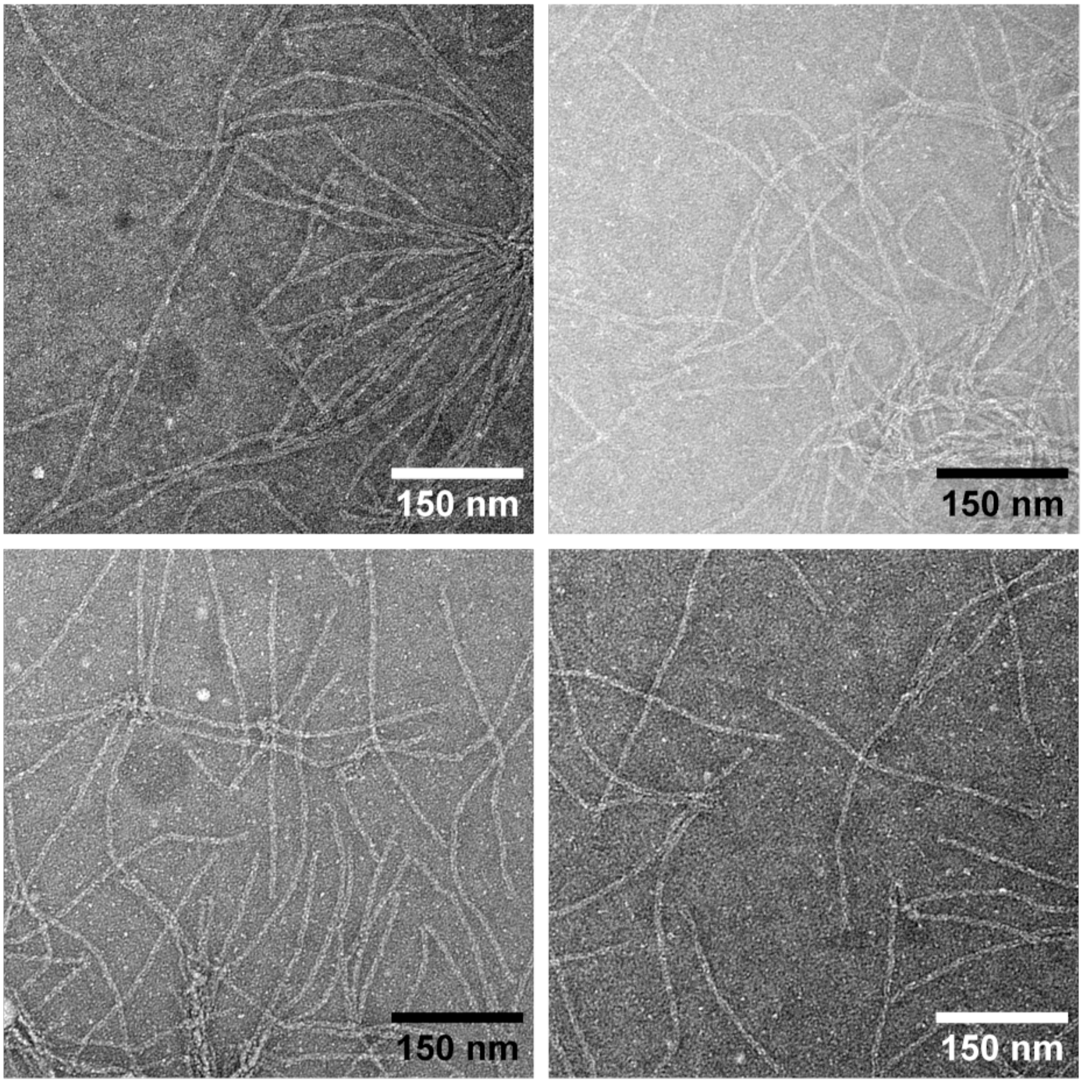

Figure S90. TEM images of 6HB in deionized water exposed to 40% (v/v) acetone for 24 h at room temperature. The TEM samples are negatively stained with uranyl formate (2% (w/v)). Acetone was removed by PEG precipitation before the TEM sample preparation.

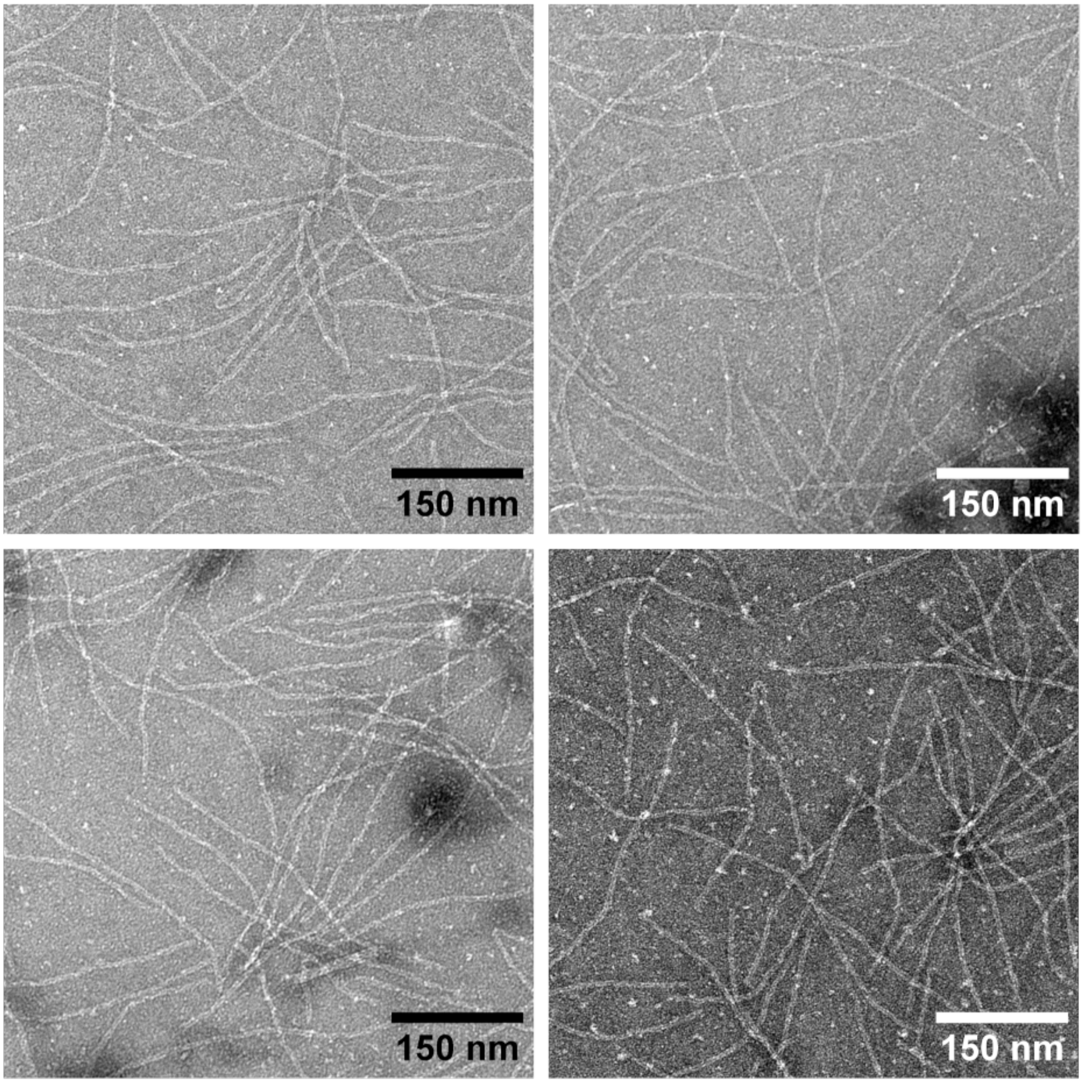

Figure S91. TEM images of 6HB in deionized water exposed to 90% (v/v) acetone for 24 h at room temperature. The TEM samples are negatively stained with uranyl formate (2% (w/v)). Acetone was removed by PEG precipitation before the TEM sample preparation.

7.5. 24HB in deionized water exposed to DMF

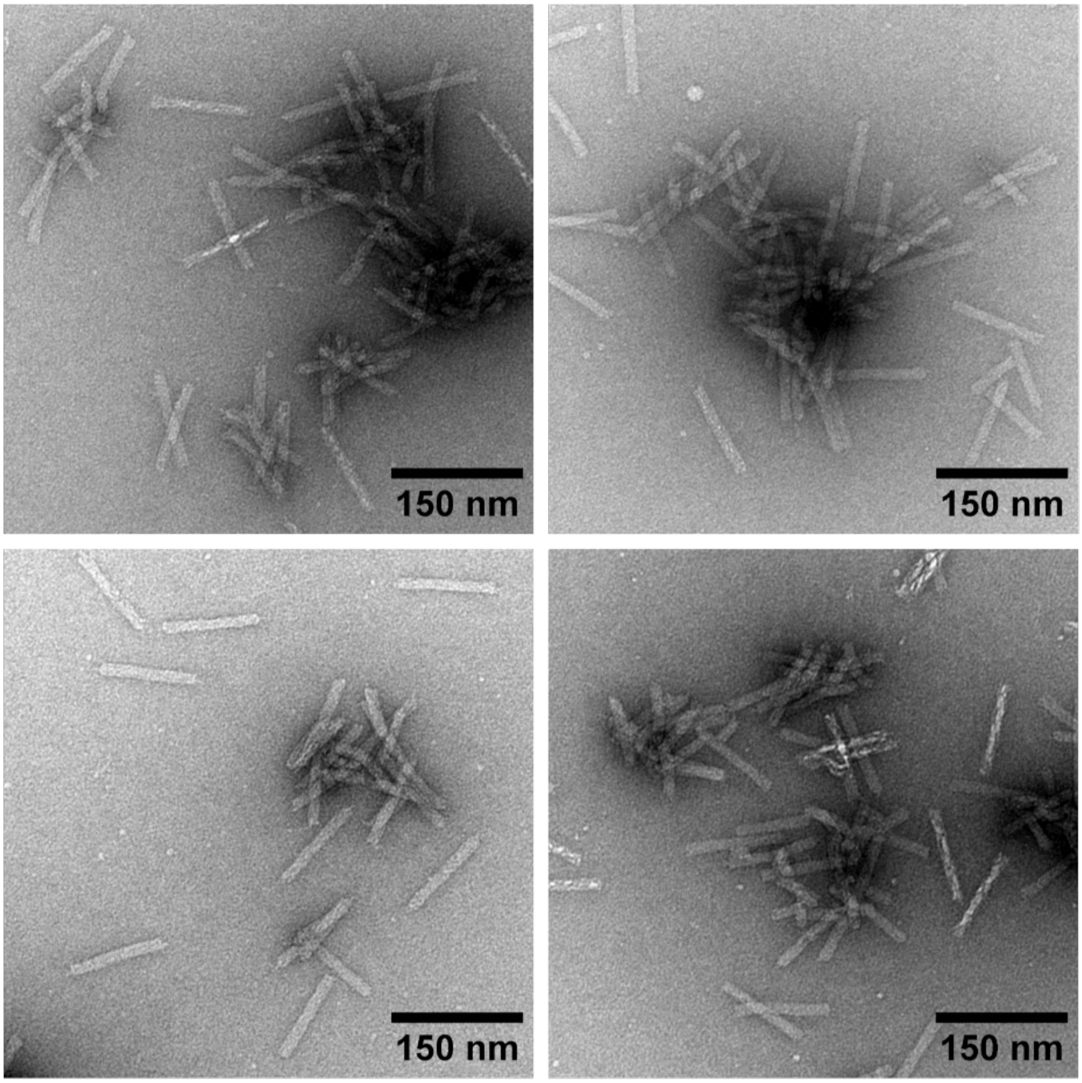

Figure S92. TEM images of 24HB in deionized water exposed to 10% (v/v) DMF for 24 h at room temperature. The TEM samples are negatively stained with uranyl formate (2% (w/v)). DMF was removed by PEG precipitation before the TEM sample preparation.

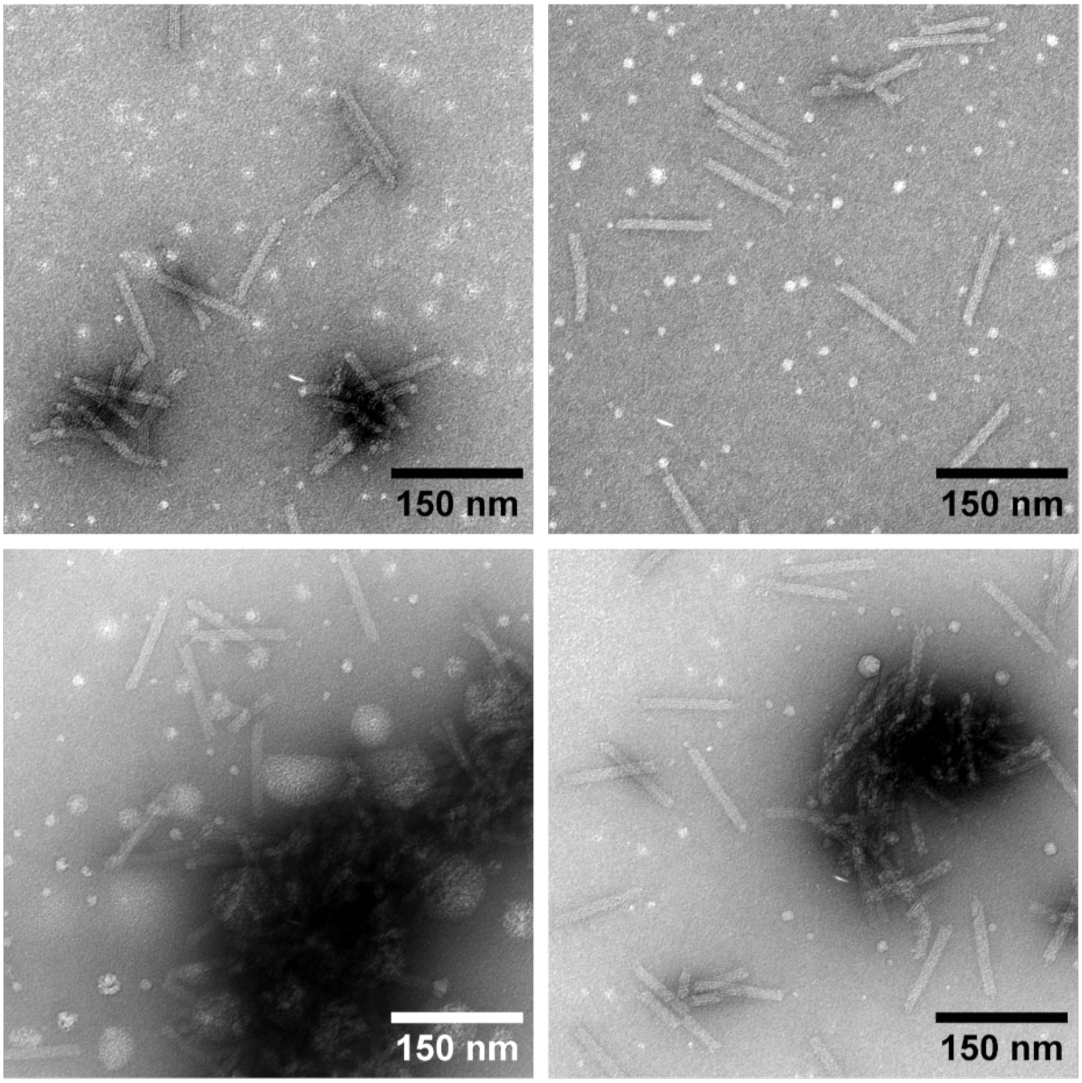

Figure S93. TEM images of 24HB in deionized water exposed to 25% (v/v) DMF for 24 h at room temperature. The TEM samples are negatively stained with uranyl formate (2% (w/v)). DMF was removed by PEG precipitation before the TEM sample preparation.

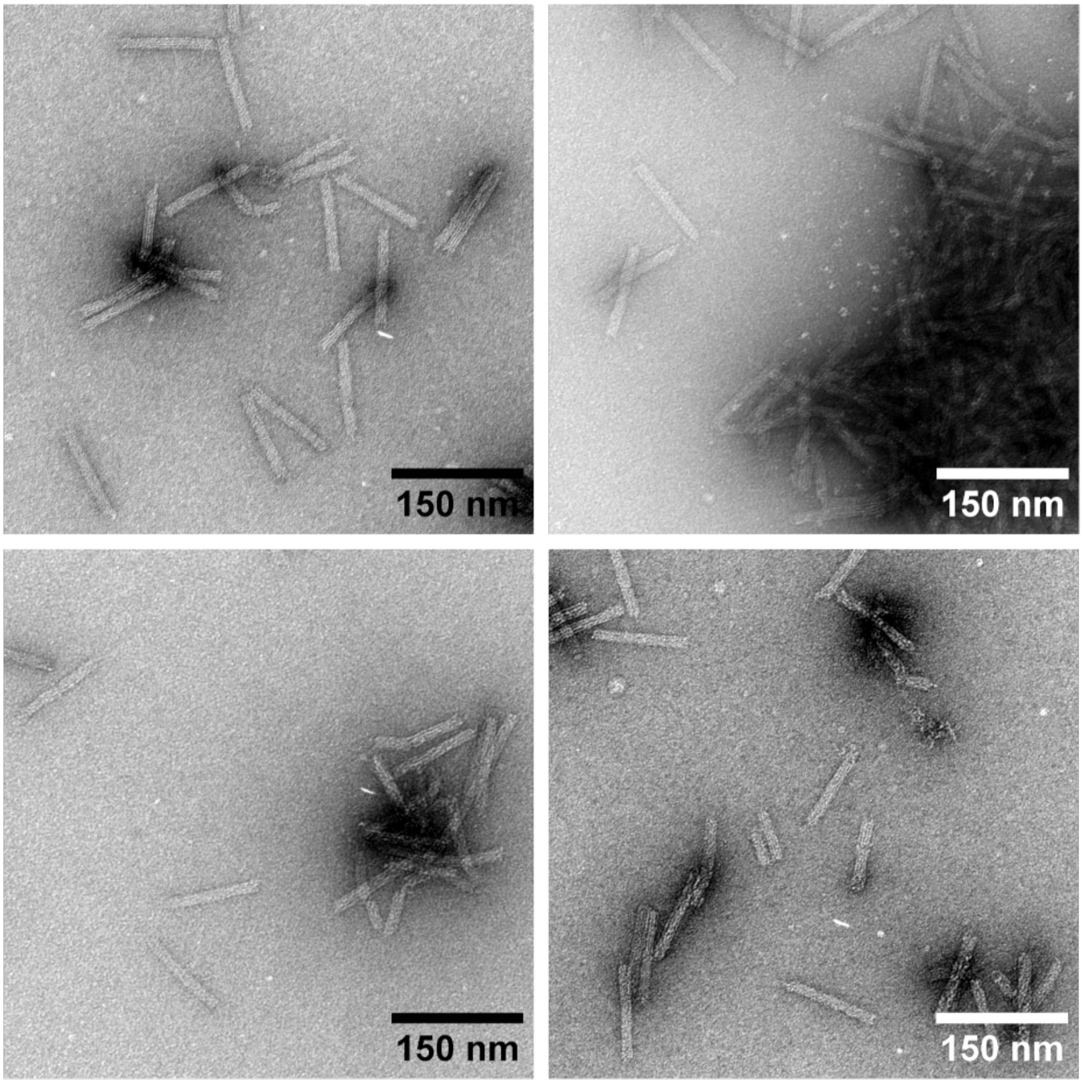

Figure S94. TEM images of 24HB in deionized water exposed to 30% (v/v) DMF for 24 h at room temperature. The TEM samples are negatively stained with uranyl formate (2% (w/v)). DMF was removed by PEG precipitation before the TEM sample preparation.

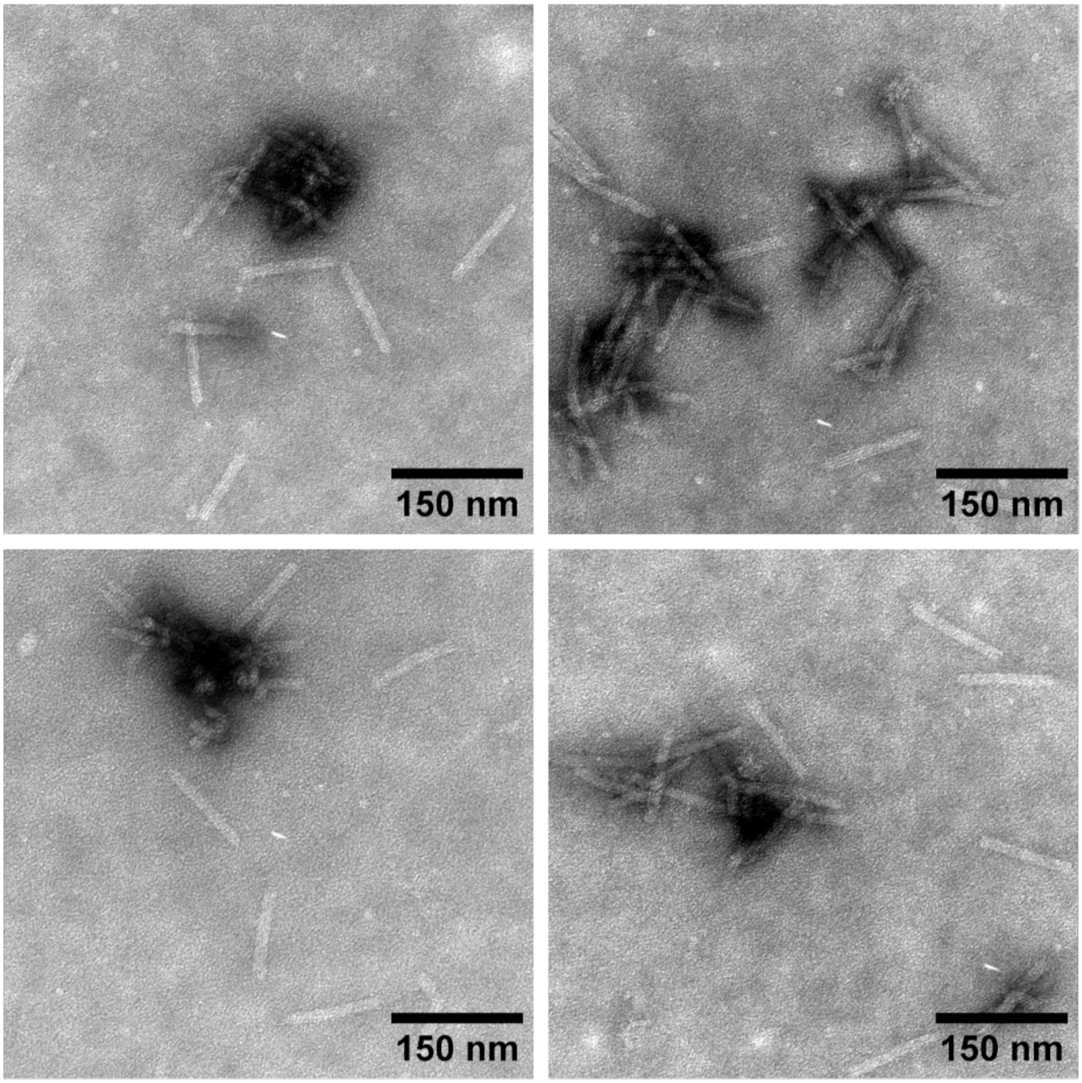

Figure S95. TEM images of 24HB in deionized water exposed to 35% (v/v) DMF for 24 h at room temperature. The TEM samples are negatively stained with uranyl formate (2% (w/v)). DMF was removed by PEG precipitation before the TEM sample preparation.

7.6. 24HB in deionized water exposed to DMSO

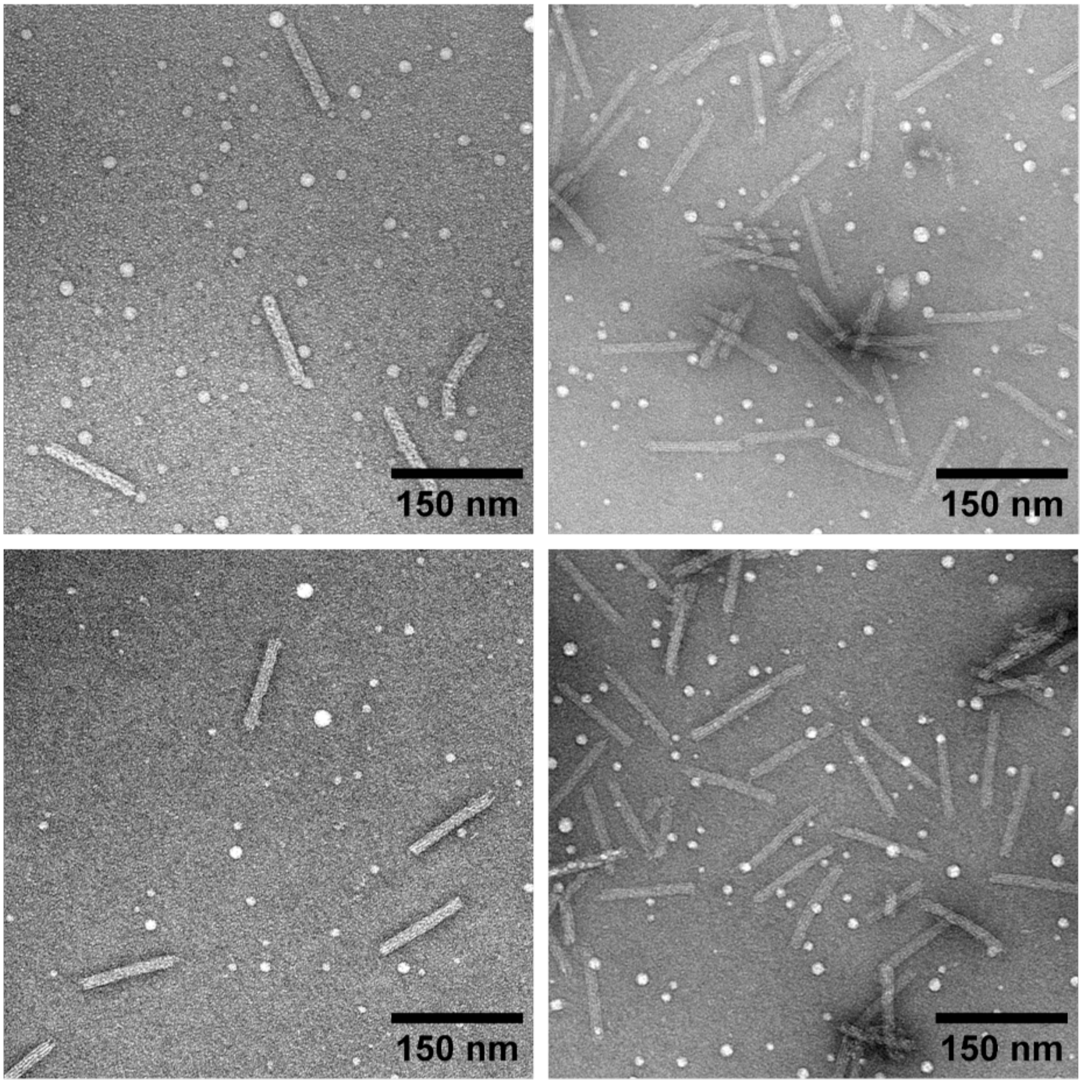

Figure S96. TEM images of 24HB in deionized water exposed to 10% (v/v) DMSO for 24 h at room temperature. The TEM samples are negatively stained with uranyl formate (2% (w/v)). DMSO was removed by PEG precipitation before the TEM sample preparation.

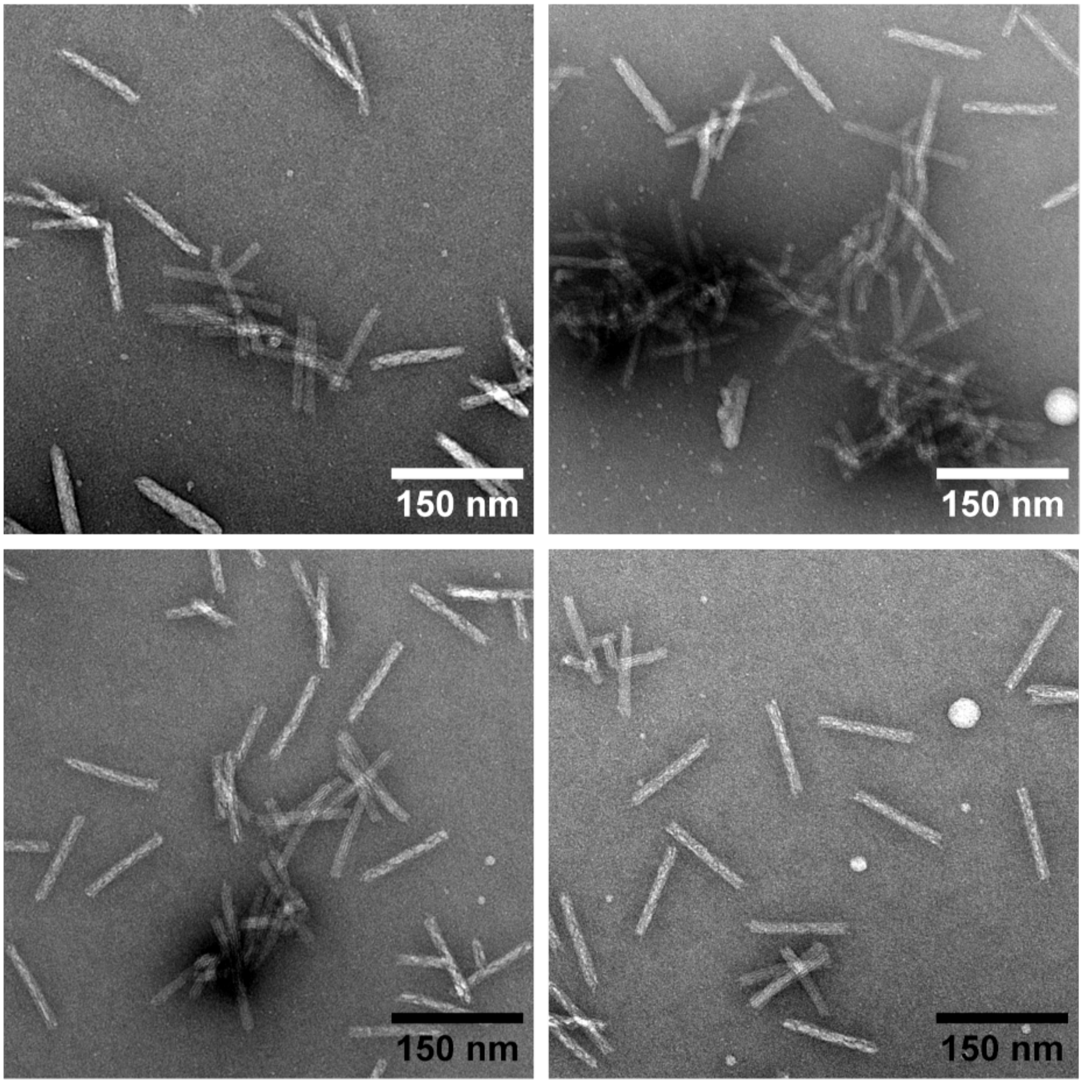

Figure S97. TEM images of 24HB in deionized water exposed to 30% (v/v) DMSO for 24 h at room temperature. The TEM samples are negatively stained with uranyl formate (2% (w/v)). DMSO was removed by PEG precipitation before the TEM sample preparation.

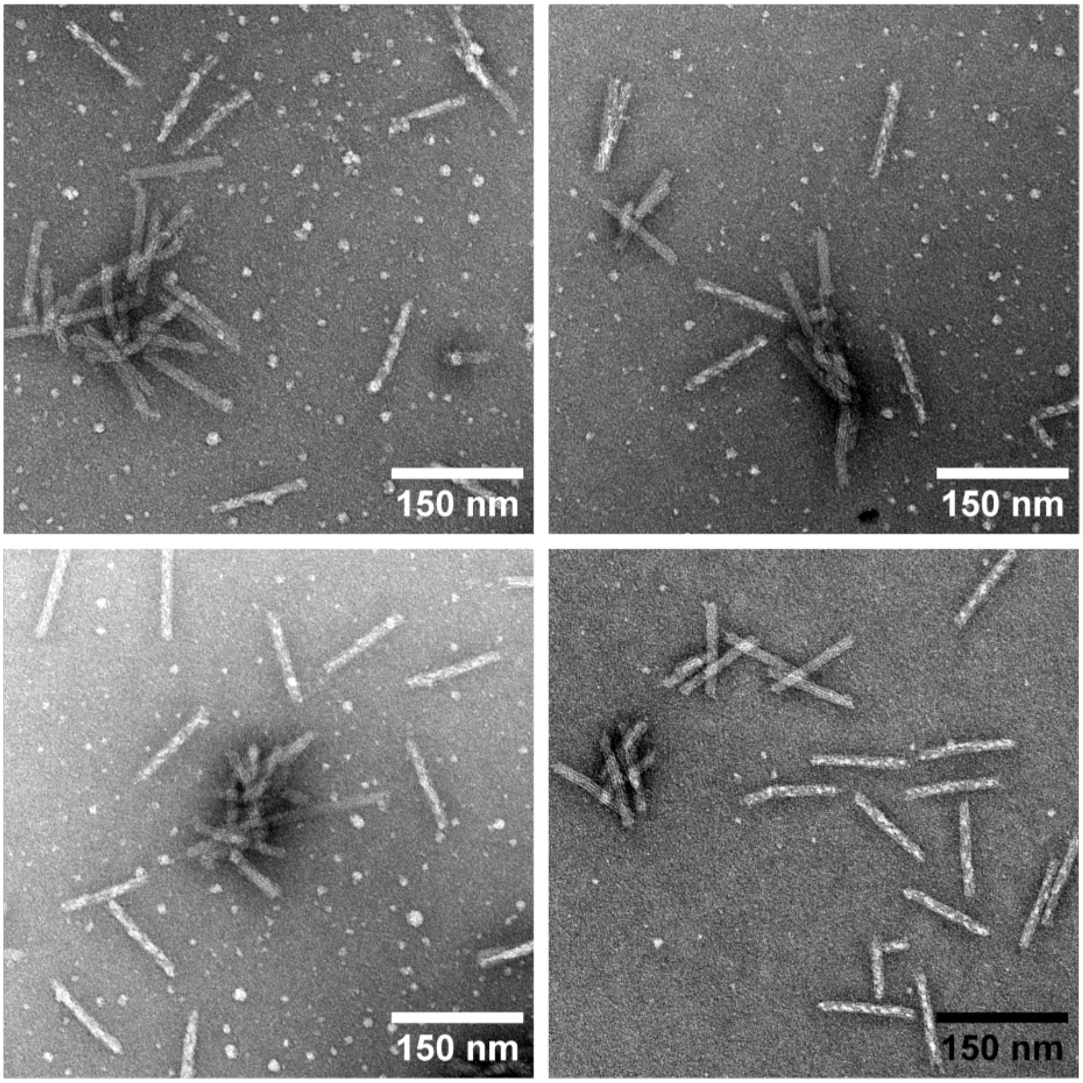

Figure S98. TEM images of 24HB in deionized water exposed to 35% (v/v) DMSO for 24 h at room temperature. The TEM samples are negatively stained with uranyl formate (2% (w/v)). DMSO was removed by PEG precipitation before the TEM sample preparation.

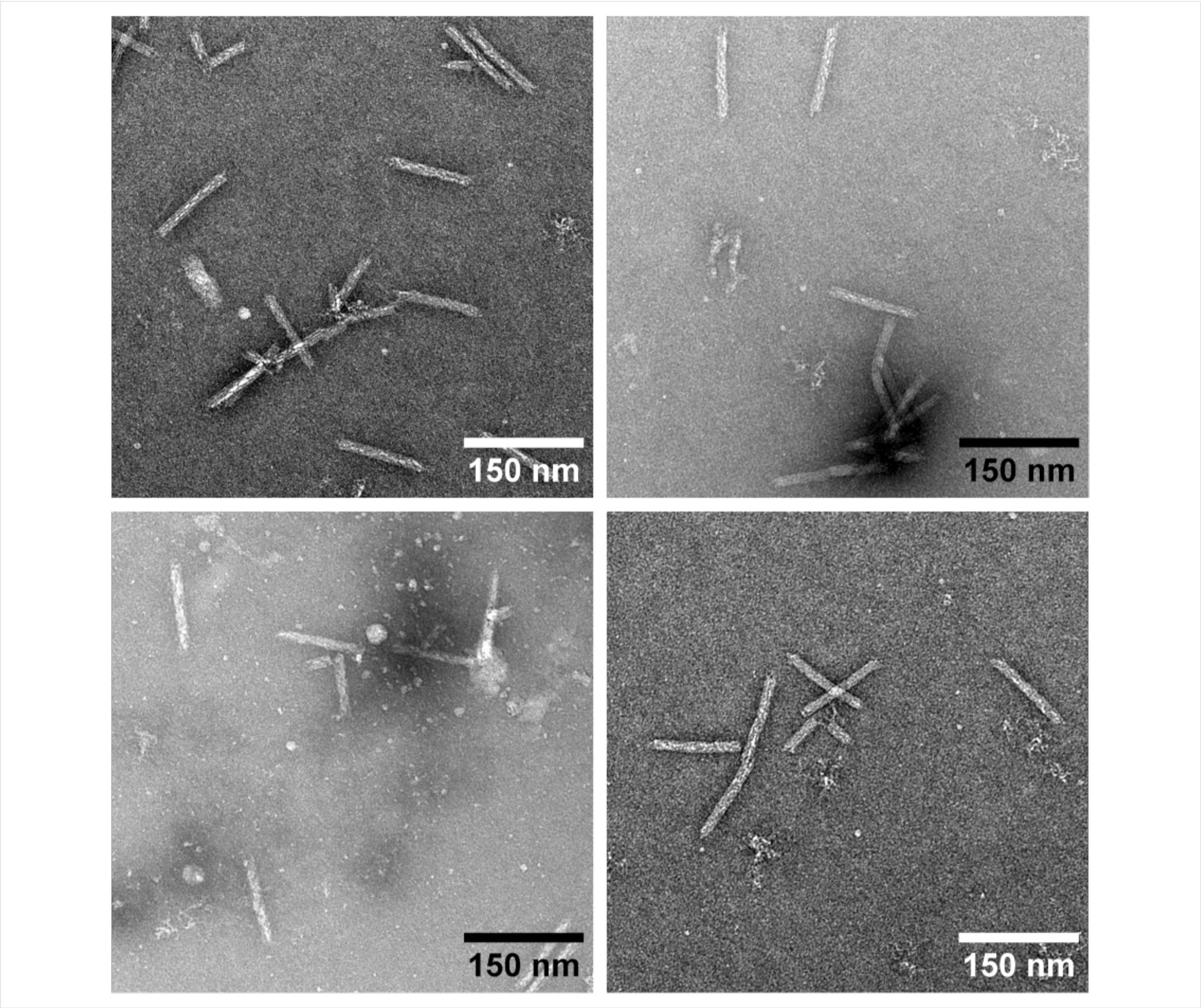

Figure S99. TEM images of 24HB in deionized water exposed to 40% (v/v) DMSO for 24 h at room temperature. The TEM samples are negatively stained with uranyl formate (2% (w/v)). DMSO was removed by PEG precipitation before the TEM sample preparation.

7.7. 24HB in deionized water exposed to ethanol

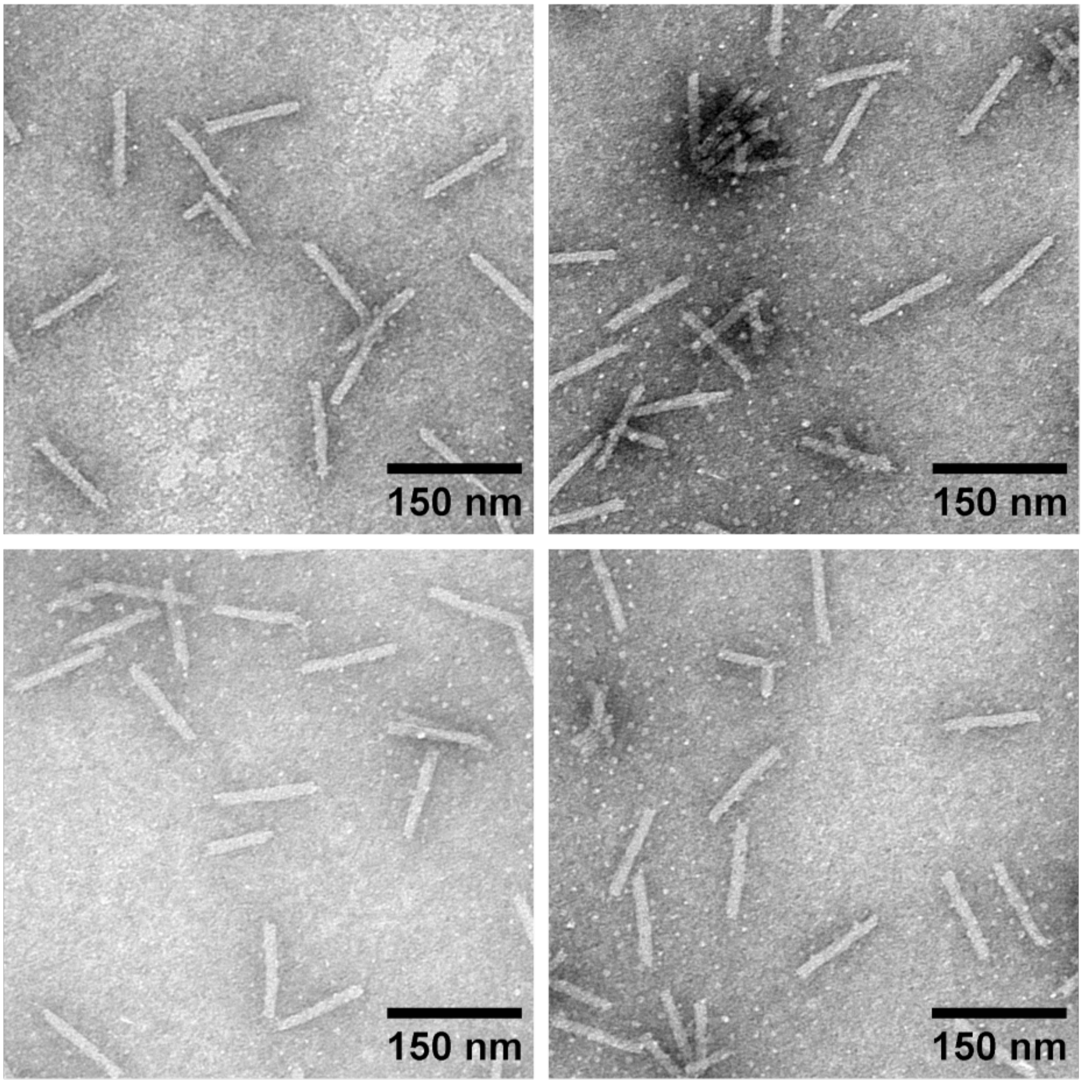

Figure S100. TEM images of 24HB in deionized water exposed to 10% (v/v) ethanol for 24 h at room temperature. The TEM samples are negatively stained with uranyl formate (2% (w/v)). Ethanol was removed by PEG precipitation before the TEM sample preparation.

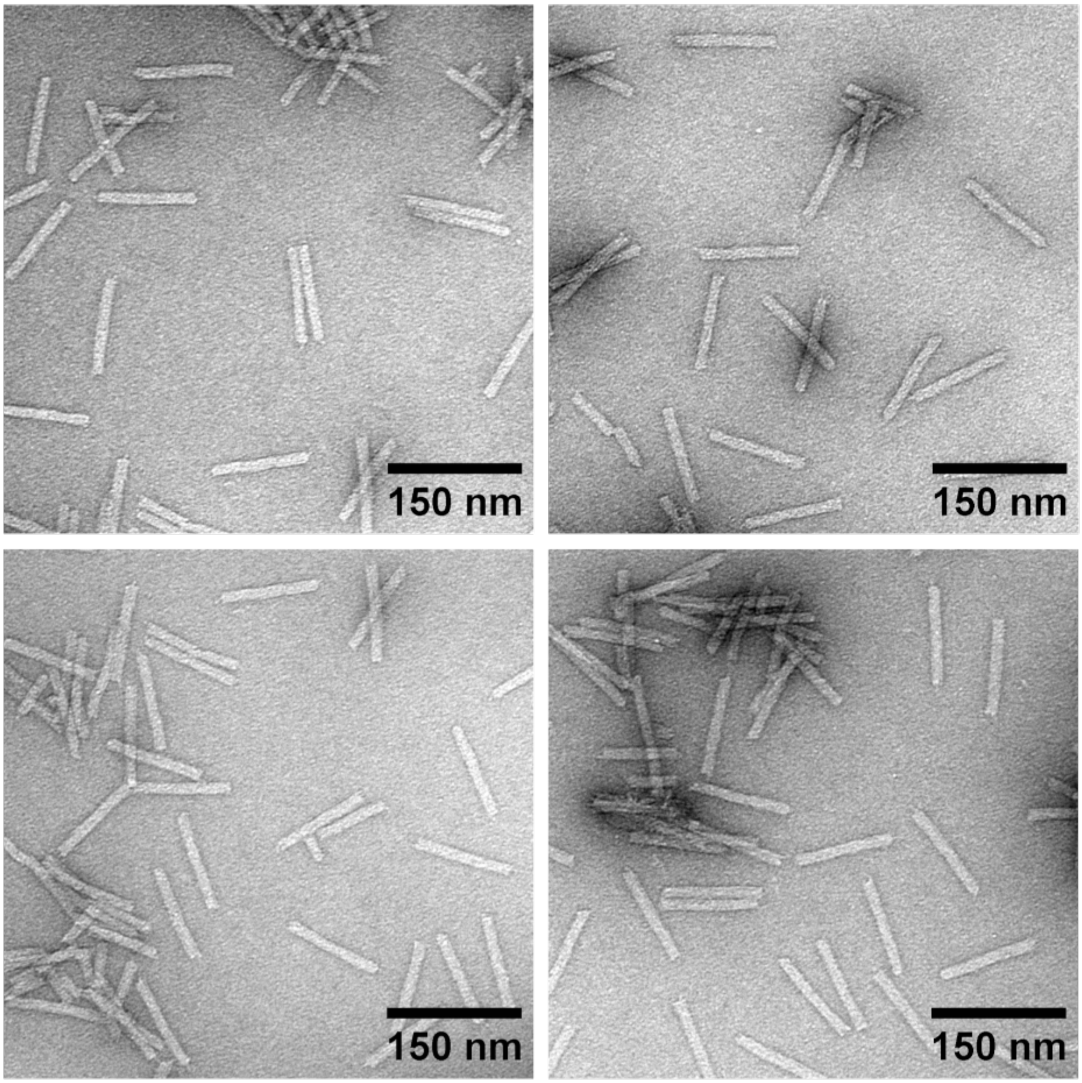

Figure S101. TEM images of 24HB in deionized water exposed to 40% (v/v) ethanol for 24 h at room temperature. The TEM samples are negatively stained with uranyl formate (2% (w/v)). Ethanol was removed by PEG precipitation before the TEM sample preparation.)

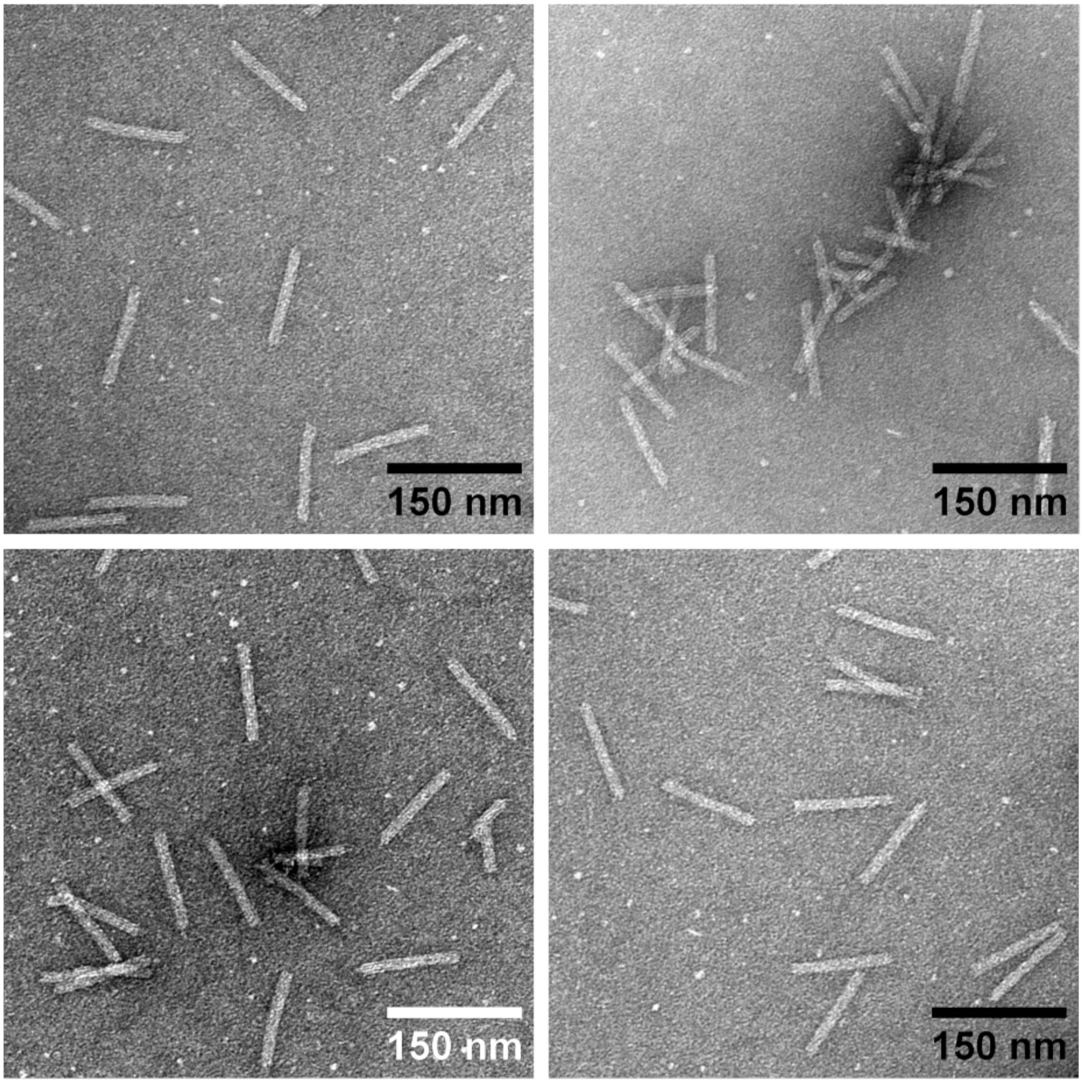

Figure S102. TEM images of 24HB in deionized water exposed to 90% (v/v) ethanol for 24 h at room temperature. The TEM samples are negatively stained with uranyl formate (2% (w/v)). Ethanol was removed by PEG precipitation before the TEM sample preparation.

7.8. 24HB in deionized water exposed to acetone

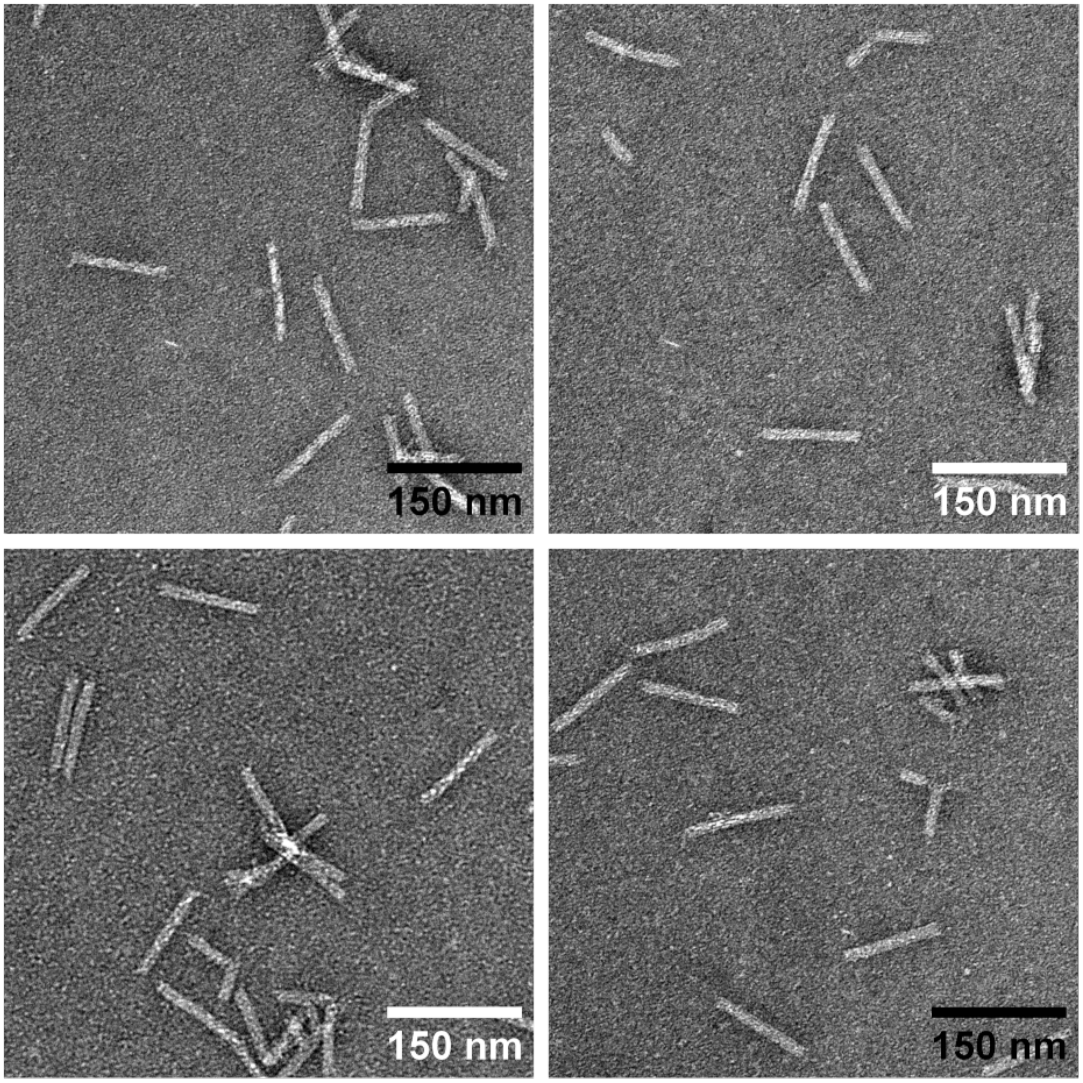

Figure S103. TEM images of 24HB in deionized water exposed to 10% (v/v) acetone for 24 h at room temperature. The TEM samples are negatively stained with uranyl formate (2% (w/v)). Acetone was removed by PEG precipitation before the TEM sample preparation.

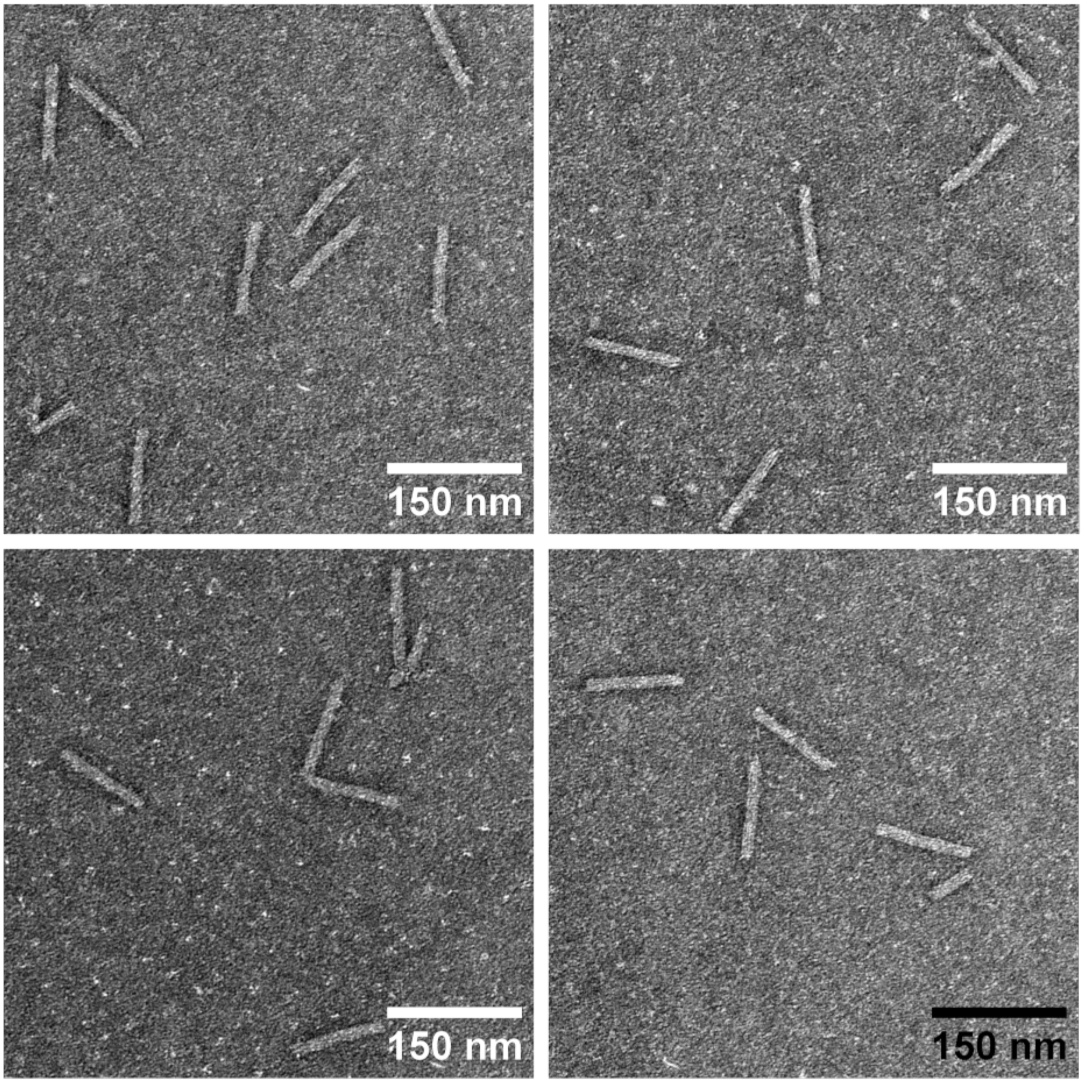

Figure S104. TEM images of 24HB in deionized water exposed to 40% (v/v) acetone for 24 h at room temperature. The TEM samples are negatively stained with uranyl formate (2% (w/v)). Acetone was removed by PEG precipitation before the TEM sample preparation.

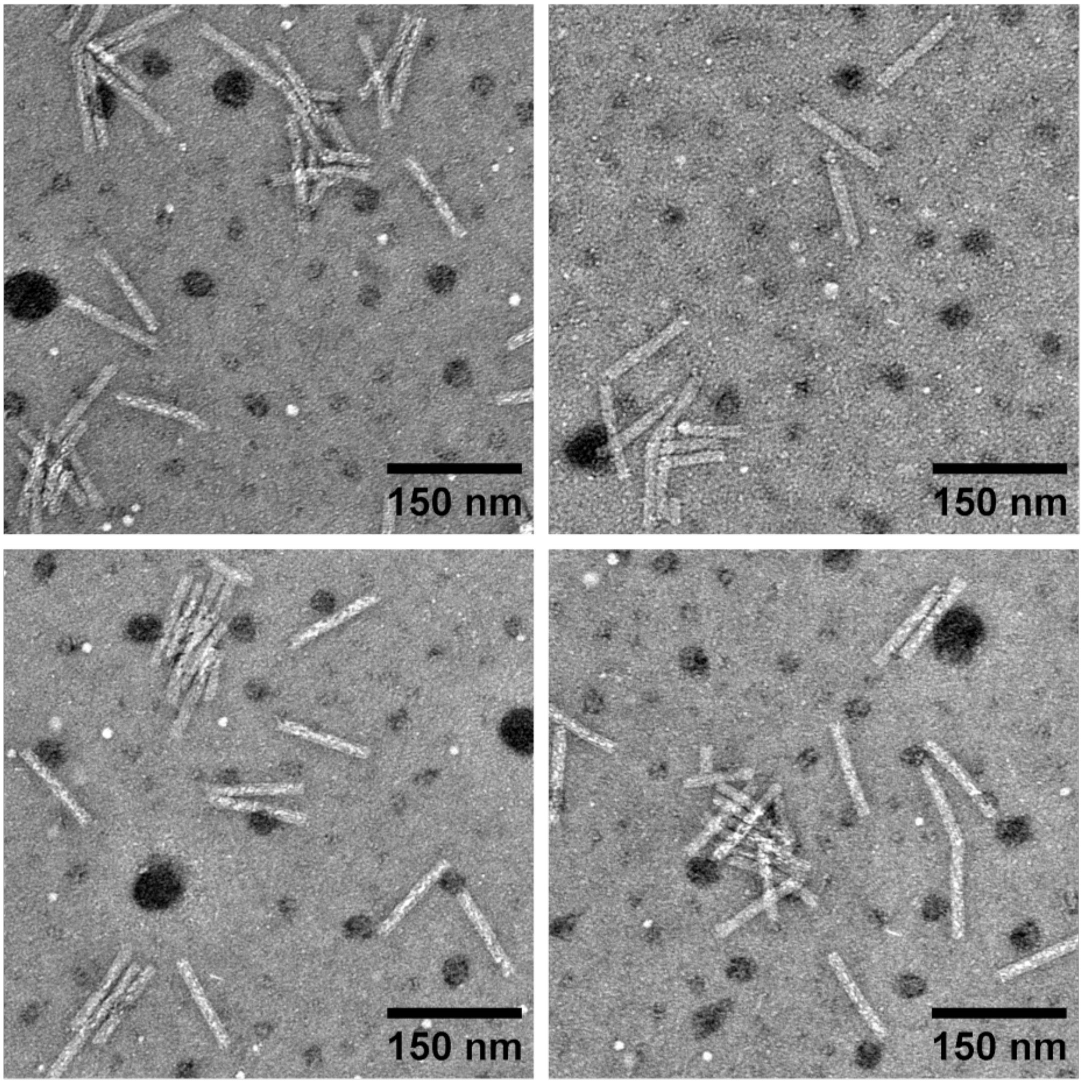

Figure S105. TEM images of 24HB in deionized water exposed to 90% (v/v) acetone for 24 h at room temperature. The TEM samples are negatively stained with uranyl formate (2% (w/v)). Acetone was removed by PEG precipitation before the TEM sample preparation.
